# The gut microbiota is associated with the small intestinal paracellular permeability and the development of the immune system in healthy children during the first two years of life

**DOI:** 10.1186/s12967-021-02839-w

**Published:** 2021-04-28

**Authors:** Mariusz Kaczmarczyk, Ulrike Löber, Karolina Adamek, Dagmara Węgrzyn, Karolina Skonieczna-Żydecka, Damian Malinowski, Igor Łoniewski, Lajos Markó, Thomas Ulas, Sofia K. Forslund, Beata Łoniewska

**Affiliations:** 1 Department of Clinical Biochemistry, Pomeranian Medical University in Szczecin, 70-111 Szczecin, Poland; 2 Experimental and Clinical Research Center, A Cooperation of Charité - Universitätsmedizin Berlin and Max Delbrück Center for Molecular Medicine, 13125 Berlin, Germany; 3 Charité-Universitätsmedizin Berlin, Corporate Member of Freie Universität Berlin, Humboldt-Universität Zu Berlin, and Berlin Institute of Health, 14195 Berlin, Germany; 4 Max Delbrück Center for Molecular Medicine in the Helmholtz Association, 13125 Berlin, Germany; 5 DZHK (German Centre for Cardiovascular Research), partner site Berlin, Berlin, Germany; 6 Department of Neonatal Diseases, Pomeranian Medical University in Szczecin, 70-111 Szczecin, Poland; 7 Department of Biochemical Sciences, Pomeranian Medical University in Szczecin, 71-460 Szczecin, Poland; 8 Department of Pharmacology, Pomeranian Medical University in Szczecin, 70-111 Szczecin, Poland; 9 Department of Human Nutrition and Metabolomics, Broniewskiego 24, 71-460 Szczecin, Poland; 10 Berlin Institute of Health (BIH), 10178 Berlin, Germany; 11 Systems Medicine, German Center for Neurodegenerative Diseases (DZNE), 53127 Bonn, Germany; 12 PRECISE Platform for Single Cell Genomics and Epigenomics at the German Center for Neurodegenerative Diseases and the University of Bonn, 53127 Bonn, Germany; 13 European Molecular Biology Laboratory, Structural and Computational Biology Unit, 69117 Heidelberg, Germany

**Keywords:** Zonulin, Calprotectin, Gut microbiota, Gut permeability, Newborn

## Abstract

**Background:**

The intestinal barrier plays an important role in the defense against infections, and nutritional, endocrine, and immune functions. The gut microbiota playing an important role in development of the gastrointestinal tract can impact intestinal permeability and immunity during early life, but data concerning this problem are scarce.

**Methods:**

We analyzed the microbiota in fecal samples (101 samples in total) collected longitudinally over 24 months from 21 newborns to investigate whether the markers of small intestinal paracellular permeability (zonulin) and immune system development (calprotectin) are linked to the gut microbiota. The results were validated using data from an independent cohort that included the calprotectin and gut microbiota in children during the first year of life.

**Results:**

Zonulin levels tended to increase for up to 6 months after childbirth and stabilize thereafter remaining at a high level while calprotectin concentration was high after childbirth and began to decline from 6 months of life. The gut microbiota composition and the related metabolic potentials changed during the first 2 years of life and were correlated with zonulin and calprotectin levels. Faecal calprotectin correlated inversely with alpha diversity (Shannon index, r = − 0.30, FDR P (Q) = 0.039). It also correlated with seven taxa; i.a. negatively with Ruminococcaceae (r = − 0.34, Q = 0.046), and Clostridiales (r = − 0.34, Q = 0.048) and positively with *Staphylococcus* (r = 0.38, Q = 0.023) and Staphylococcaceae (r = 0.35, Q = 0.04), whereas zonulin correlated with 19 taxa; i.a. with Bacillales (r = − 0.52, Q = 0.0004), Clostridiales (r = 0.48, Q = 0.001) and the *Ruminococcus* (*torques* group) (r = 0.40, Q = 0.026). When time intervals were considered only changes in abundance of the *Ruminococcus* (*torques* group) were associated with changes in calprotectin (β = 2.94, SE = 0.8, Q = 0.015). The dynamics of stool calprotectin was negatively associated with changes in two MetaCyc pathways: pyruvate fermentation to butanoate (β = − 4.54, SE = 1.08, Q = 0.028) and *Clostridium acetobutylicum* fermentation (β = − 4.48, SE = 1.16, Q = 0.026).

**Conclusions:**

The small intestinal paracellular permeability, immune system-related markers and gut microbiota change dynamically during the first 2 years of life. The *Ruminococcus* (*torques* group) seems to be especially involved in controlling paracellular permeability. *Staphylococcus,* Staphylococcaceae, Ruminococcaceae, and Clostridiales, may be potential biomarkers of the immune system. Despite observed correlations their clear causation and health consequences were not proven. Mechanistic studies are required.

**Graphic abstract:**

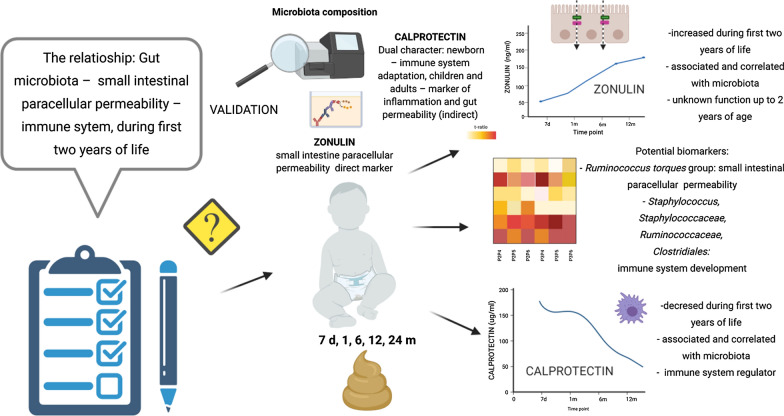

**Supplementary Information:**

The online version contains supplementary material available at 10.1186/s12967-021-02839-w.

## Background

The intestinal barrier plays important role in the defense against infections, apart from its essential nutritional, endocrine, and immune functions [1]. Multiple factors impact intestinal permeability in infants, including gestational age, mode of delivery and feeding, and various diseases [2]. Increased gut barrier permeability allows the optimal nutrient uptake and leads to increased immune tolerance [3]; on the other hand, increased permeability to foreign antigens, including intestinal bacteria, might result in inflammation and systemic hypersensitivity [3, 4]. The gut barrier of a newborn is highly permeable; importantly, the permeability decreases during a process known as “gut closure” [5]. The exact time of this process in humans, regulated by growth factors, hormones and breast milk is unknown but has been proposed to take place around 22nd week of life [6]. It could also be hypothesized that gut microbiota may be involved in this process [6, 7]. Of note, gut permeability can be assessed via the analysis of the absorption of various substances [8], as well as via the measurement of blood and stool markers, including zonulin and calprotectin [9].

Zonulin (ZON), an analog of the cholera comma toxin (ZOT, zonula occludens toxin) [10, 11] shown to regulate paracellular transport within the small bowel [12–14]. This protein is synthesized in the liver and epithelial cells of the intestine and is a component of multi-protein membrane complexes (claudin-occludin-guanylate kinase-like proteins zonula occludens (ZO)-1, ZO-2, and ZO-3) forming tight junctions (TJ) [15]. In fact, zonulin regulates the tightness of TJ, which is an important part of the proper function of the intestinal barrier [16]. Increased zonulin concentrations correlate positively with small intestinal permeability [17], a phenomenon discovered in the context of inflammatory and autoimmune disease [18–21], and could be considered as a biomarker for systemic inflammation [22].

Human calprotectin is a 24 kDa dimer formed by the protein monomers S100A8 (10,835 Da) and S100A9 (13,242 Da), and makes up to 60% of the soluble proteins in the cytosol of human neutrophils [23, 24]. The sources of calprotectin in newborns are breast milk and resident and non-resident myeloid cells [25]. In fact, fecal calprotectin is used in older children and adults as a marker for inflammatory bowel diseases (IBD); there is a body of evidence correlating calprotectin with IBD, cow-milk allergy, atopic disease and other gastrointestinal disorders [26]. Of note, calprotectin has the potential to be used as an indirect marker of gut permeability [27]. However, its role as a marker of inflammation was not confirmed in neonates [25].

In our previous study [28] we found that some maternal–fetal factors are associated with increased concentration of fecal calprotectin and zonulin in children during the first 2 years of life. Additionally, we found that after birth, zonulin levels increased up until 12 months of age, remaining high thereafter, while calprotectin levels decreased until six months of age, stabilizing thereafter [29]. Of note, in this previous study, we did not assess the fecal microbiota, which was a significant limitation; we were not able to trace the relationships between the fecal microbiome and the concentrations of the abovementioned stool markers. Therefore, in this study, we decided to analyze the microbiota in faecal samples collected longitudinally over 24 months from 21 subjects to test the hypothesis that small intestinal barrier permeability is linked to the gut microbiota. Moreover, we validated the observed results via comparison with the data obtained in the study by Willers et al. (Hannover Medical School—HMS cohort) who measured the relationship between calprotectin and gut microbiota in children during the first year of life [25].

## Methods

### Subjects

The present observational prospective cohort study continues research efforts in the context of intestinal barrier function in a cohort of Polish newborns, described in detail in previous publications [28, 29]. Twenty four healthy full-term newborns at the Department of Obstetrics, Gynecology and Neonatology, the Pomeranian Medical University/Independent Public Clinical Hospital No. 2 in Szczecin (PMU cohort) were initially recruited to this study. Longitudinal sampling was performed for over 24 months. Three newborns were excluded due to an inadequate number of samples. In total, 21 newborns (101 samples in total) were included in the study, with at least four longitudinal stool samples available (the sample availability matrix is provided in Additional file 1: Figure S1). Samples were taken at the following points in time—meconium (P1, n = 8), 7 days (P2, n = 20), 1 month (P3, n = 19), 6 months (P4, n = 20), 12 months (P5, n = 21) and 24 months (P6, n = 13) (Additional file 1: Figure S2) after birth. The study was conducted using the results obtained for the time points P2-P6. The measured markers and the meconium microbiota composition (P1) reflect the perinatal period. Therefore the values obtained at this time point were used instead to validate the microbiota analysis (the meconium microbiota is expected to be clearly distinguishable from the microbiota obtained at other time points). The participants’ characteristics are shown in Table 1.

**Table 1:** Characteristics of study participants

Characteristic	P1 (n = 8**)**	P2 (n = 20**)**	P3 (n = 19)	P4 (n = 20)	P5 (n = 21)	P6 (n = 13)
Sex (F/M)	4/4	9/11	10/9	9/11	10/11	8/5
Mode of delivery (C/V)	5/3	11/9	11/8	12/8	12/9	6/7
Birth weight (g)	3472 ± 507	3320 ± 548	3311 ± 518	3322 ± 550	3341 ± 543	3352 ± 582
Body weight (kg)	–	3.36 ± 0.54	4.49 ± 0.74	8.26 ± 1.18	10.40 ± 1.29	13.00 ± 1.68
Feeding method (N/F)	8/0	20/0	15/4	9/11	4/17	0/13
Breastfeeding time (weeks)	36.2 ± 25.6	30.0 ± 26.7	27.1 ± 24.9	27.9 ± 26.9	28.9 ± 26.6	27.1 ± 26.3

C, Cesarean section, V, vaginal delivery, N, natural, F, formula; P1, meconium, P2, 7th day, P3, 1st month, P4, 6th month, P5, 12th month, P6, 24th month

All included newborns were exclusively breastfed during the first week of life. The children were healthy and did not take antibiotics at the time the samples were collected. For validation, we used independent Illumina MiSeq 16S rRNA data of the same hypervariable region (V3–V4) derived from 227 stool samples of healthy term children enrolled in the HMS cohort. They were tested at seven time points (1, 2, 10, 30, 90, 180 and 360 days of age). For the sake of compatibility with the PMU cohort, five time points were selected: 1 day (P1, n = 43), 10 days (P2, n = 42), 30 days (P3, n = 47), 180 days (P4, n = 43) and 360 days (P5, n = 52) of age.

### Determination of the fecal zonulin and calprotectin content

The concentrations of zonulin and calprotectin in the fecal samples were measured as previously described [28, 29].

### Sample collection, DNA extraction and sequencing

Stool samples were collected from diapers using a standardized biological material collection kit (Stool Sample Application System (SAS); Immundiagnostik, Bensheim, Germany). The samples were collected by previously trained hospital staff or by the parents according to an established procedure and stored in a refrigerator (for a maximum of eight hours) before transport. Transport to the laboratory took no longer than 60 min, at 6–8 °C. The stool samples were then frozen at − 20 °C until metagenomic analyses were conducted. Microbiome DNA extraction was performed using the Genomic Mini AX Bacteria + Spin and Genomic Mini AX Soil Spin kits (A&A Biotechnology, Gdynia, Poland) following the manufacturer’s protocol. DNA concentrations were determined by fluorometry (DeNovix DS-11 FX + Spectrophotometer/Fluorometer, Wilmington, DE, USA). Samples were subsequently stored at − 20 °C. Metagenomic libraries of the V3–V4 hypervariable region of the 16S rRNA gene were constructed and further sequenced on the MiSeq platform (paired-end 2 × 250 bp) using V2 chemistry from Illumina (Illumina, San Diego, CA, USA). Next generation sequencing (NGS) was performed by Genomed S.A., Warsaw, Poland.

For validation, we used the independent Illumina MiSeq 16S rRNA data of the same hypervariable region (V3–V4) derived from the 227 stool samples of healthy term children enrolled in the HMS cohort.

### 16S sequencing sample processing

The sequences were processed using LotuS 1.62 [30]. LotuS clusters operational taxonomic units and generates taxonomic-level abundances tables. UPARSE de novo sequence clustering removed chimeric OTUs with 1470 OTUs (PMU cohort) and 923 OTUs (HMS cohort) remaining. OTU seed sequences were classified by BLAST lower ancestor comparison to SILVA (1.32). The Rarefaction Toolkit (RTK) was used to normalize the abundances on all taxonomic levels [31]. Two samples were removed due to their low number of bacterial reads from the PMU cohort (read counts of 246 and 134), resulting in a final rarefaction depth of 1117 16S reads, yielding a count of 594 genera resolved at this sequencing depth and the final sample size of 101 was obtained. Moreover, 108 samples were removed due to their low number of bacterial reads from the HMS cohort (read count less than 1000), resulting in a rarefaction depth of 1000 16S reads, yielding 484 genera and the final sample size of 227.

The alpha diversity (Shannon, Simpson, Chao1, Evenness**)** was computed using RTK taking the genus median alpha diversity over ten rarefaction cycles. The beta diversity was calculated on a rarefied genus-level abundance table using the Bray–Curtis dissimilarity metrics and visualized as principal coordinate analysis (PCoA). Taxa that were present in less than 20% (PMU cohort) and 10% (HMS cohort; a lower taxa prevalence was used to allow the comparison) of samples were removed.

To predict the metabolic profile of gut microbiota, we used the PICRUSt2 tool [32]. The prediction of MetaCyc metabolic pathways was conducted on the non-rarefied OTU abundance table from which rare OTUs (present in less than 20% of samples with a minimum count of 3) were removed. In order to address the issue of compositional data, the taxa and predicted MetaCyc pathway abundances (not rarefied but filtered to match the rarefied data) were transformed by generating 128 Monte Carlo instances of the Dirichlet distribution for each gut sample, followed by center-log transformation of each instance [33]. Analyses were performed for each instance separately and the results were then averaged over 128 instances.

### Power calculation

With N = 21 time series, it is true our statistical power may be limited. While this would not decrease confidence in findings where we conclude significance (type I errors) it may place us at risk for missing associations, and challenges any interpretation of negative findings. As both permeability markers and microbiome features shift over time as the gut matures and diet shifts, their association may be driven by shared dependency on this third factor. First, we consider, as the closest representative test for which power calculations are straightforward, the analysis of coupled changes in e.g. a permeability marker versus microbial taxa through (Pearson) correlation with N = 21 samples each paired across two time points, resulting in N = 21 time point differences. We consider multiple testing considerations here to be reflected as per Bonferroni correction for Nt = 50 gut genera, making for an adjusted alpha = 0.1/50 = 0.002. Under convention of considering r = 0.5 as a "large" correlation, we achieve (running as "pwr.r.test (alternative = "two.sided", n = 21, sig.level = 0.002, r = 0.5)") a statistical power of only ~ 21%, meaning it is likely that we are overly conservative due to limited sample size, and that there may be several more of these associations at least this strong which remains to be shown robust in a larger study. However, this does not detract from our confidence in the associations where we do conclude significance, as it rather is more likely we miss associations between permeability and microbiota. This has no real bearing on whether the associations are direct or indirect via time progressing both as a third factor, however. Here, support of a direct link would come from the association achieving significance not only for the correlation of differences within time courses between time points, but between donors within time points. This corresponding power calculation here is essentially the same (Nt = 21, alpha = 0.002, two-sided, taking r = 0.5 as a "large" correlation) meaning our power is also ~ 21% at concluding an effect of similar size is a direct association. However, as we are similarly underpowered to conclude significance there, we expect to underestimate rather than overestimate inference of direct versus indirect relationships. All in all thereforewe cannot be fully certain either way whether concluded associations are direct or indirect, which concern must be kept in mind in comparison of our findings to previous literature.

### Statistical analysis

All statistical analysis was performed using R (version 4.0.0, R Foundation for Statistical Computing, Vienna, Austria). Changes over time in the context of permeability biomarkers (zonulin and calprotectin), the gut alpha diversity indices, gut composition principal coordinate scores, or gut taxa and pathway abundances were analyzed using mixed-effects models. Time points (P) were treated as the fixed effect and the newborn IDs as the random effect, accounting for repeated sampling of the same individual. The significance of the time point variable was tested via comparison of the likelihoods of the two nested models (likelihood ratio test, LRT): without (null model) and with (full model) the fixed factor in question. Pairwise comparisons between timepoints were performed using the emmeans package [34] and the Benjamini–Hochberg procedure for the control of the false discovery rate (FDR). In the models with biomarkers (which were rank-transformed), taxa and metabolic pathways, the mode of delivery and breastfeeding time were also included as additional covariates and thus controlled for. The LRT p-values for taxa and metabolic pathways were FDR-adjusted as above.

The gut microbial diversity, composition and metabolic pathways were analyzed with respect to zonulin and calprotectin biomarkers using a repeated measures correlation method [35]. The repeated-measures correlation (an equivalent to the linear mixed-effects model with random intercept) was used to assess the association between the levels of the biomarkers and the alpha diversity, PCoA scores, taxa and metabolic pathway abundance, accounting for repeated measures for each participant.

In addition, an association between the differences (changes) among all time points were associated using linear mixed-effect models with random intercepts (newborn ID as a random factor). The extent of the biomarker change was defined as the dependent variable (DV). Then, the time period/interval—a categorical variable with ten levels indicating all possible differences (e.g. P2–P3, P2–P4, etc.) between the five time points (P2, P3, P4, P5 and P6, PMU cohort) or four time points (P2, P3, P4, P5, HMS cohort) and either beta-diversity (Bray–Curtis intersample composition distance) or univariate metrics of changes in microbiota composition or metabolic pathway abundance (between corresponding time points) were defined as fixed effects. To investigate the connection between the change in the overall compositional profile and the change in the levels of biomarkers, we took the within-subject Bray–Curtis distances (computed at the genus level using rarefied abundances). Fine-grained analysis was performed using changes in the taxonomic composition (from genus to phylum level) and metabolic pathways which were computed using the center-log transformed Monte Carlo instances of the Dirichlet distribution. Two sets of likelihood ratio tests were performed (with mode of delivery and breastfeeding time as covariates). In the first LRT, a significance of the Bray–Curtis distance or change in abundance was assessed under the assumption of no interaction between the fixed factors (the common slope model). The second LRT was used to determine whether the interaction was statistically significant. In case of a significant interaction effect, the emmeans package was used (emtrends function) to estimate the individual slopes over levels of the time period/interval. The p-values for the slopes (coefficients) were determined and adjusted for multiple testing to control for the FDR. No imputation was undertaken for missing data so as not to make unwarranted assumptions on the distribution of measured features. Instead these cases were consistently omitted under the settings used for the R functions employed in our analysis. Data was manipulated using Perl. A significance level of 5% was used for raw and FDR adjusted p-values.

## Results

### Stool levels of zonulin and calprotectin increase and decrease over time, respectively

**Fig. 1:**
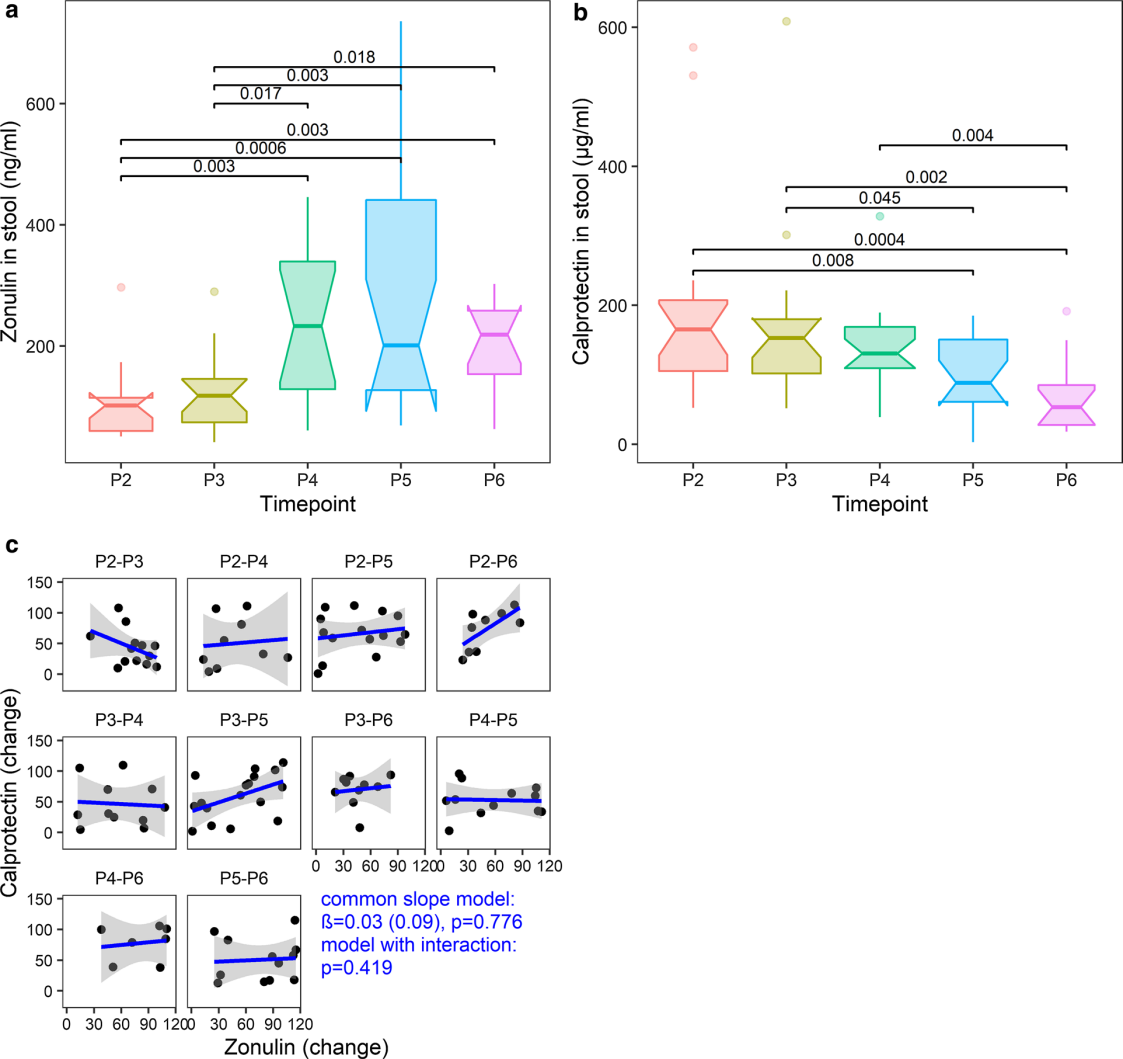
Dynamics of stool zonulin and calprotectin levels. **a** zonulin, **b** calprotectin. Notched boxplots with variable widths proportional to the square-roots of the number of observations in the groups are shown; FDR adjusted p-values < 0.05 are represented. **c** correlation between the stool zonulin and calprotectin changes (rank-transformed, adjusted for the mode of delivery and breastfeeding time); P2-7th day, P3-1st month, P4-6th month, P5-12th month, P6-24th month; PX-PY-difference between the time points X and Y

Both stool zonulin and calprotectin concentrations changed significantly over time in the cohort (Fig. 1, LRT, df = 4, P = 2.6e−05, and df = 4, P = 10.0e−05, respectively; adjusted for the mode of delivery and breastfeeding duration).

Zonulin levels at P2 (7 days after birth) and P3 (1 month after birth) were significantly lower than those at the time points P4, P5, and P6. However, from P4 to P6 (6th and 24th months of life), no significant differences were observed (between any two time points, non-consecutive or consecutive). Thus, zonulin levels tended to increase for up to 6 months after birth (P4) and stabilize thereafter (Fig. 1a). In contrast to zonulin, calprotectin levels did not differ significantly between any consecutive time points (Fig. 1b). Moreover, up to and including P4, none of the differences in calprotectin levels between any two time points (non-consecutive or consecutive) were significant. However, the calprotectin levels at P5 were significantly lower than those at P2, P3; additionally, the calprotectin levels at P6 were also significantly lower than those at P2, P3, and P4 (Fig. 1b). Overall, these results suggest that between about 6 and 12 months of age, the zonulin levels stabilize remaining at a high level, while calprotectin concentration begins to decline. Importantly, the analysis of the HMS validation cohort (Additional file 1: Figure S3) confirmed the declining trend of calprotectin from P3 onwards; of note, a significant difference was determined between the first and sixth months of life, which altogether strongly highlights the sixth month of life as an important time point concerning changes of analyzed markers after childbirth. Interestingly, despite the observed opposite trends in the context of stool zonulin and calprotectin levels, the two markers (its changes) did not appear to correlate with each other (LRT; common slope model: df = 1, P = 0.776, β = 0.03, SE = 0.09; interaction model: df = 9, P = 0.419, Fig. 1c).

### Gut microbiota diversity, taxonomy and metabolic pathways during the first two years of life

#### Gut microbiota diversity

**Fig. 2:**
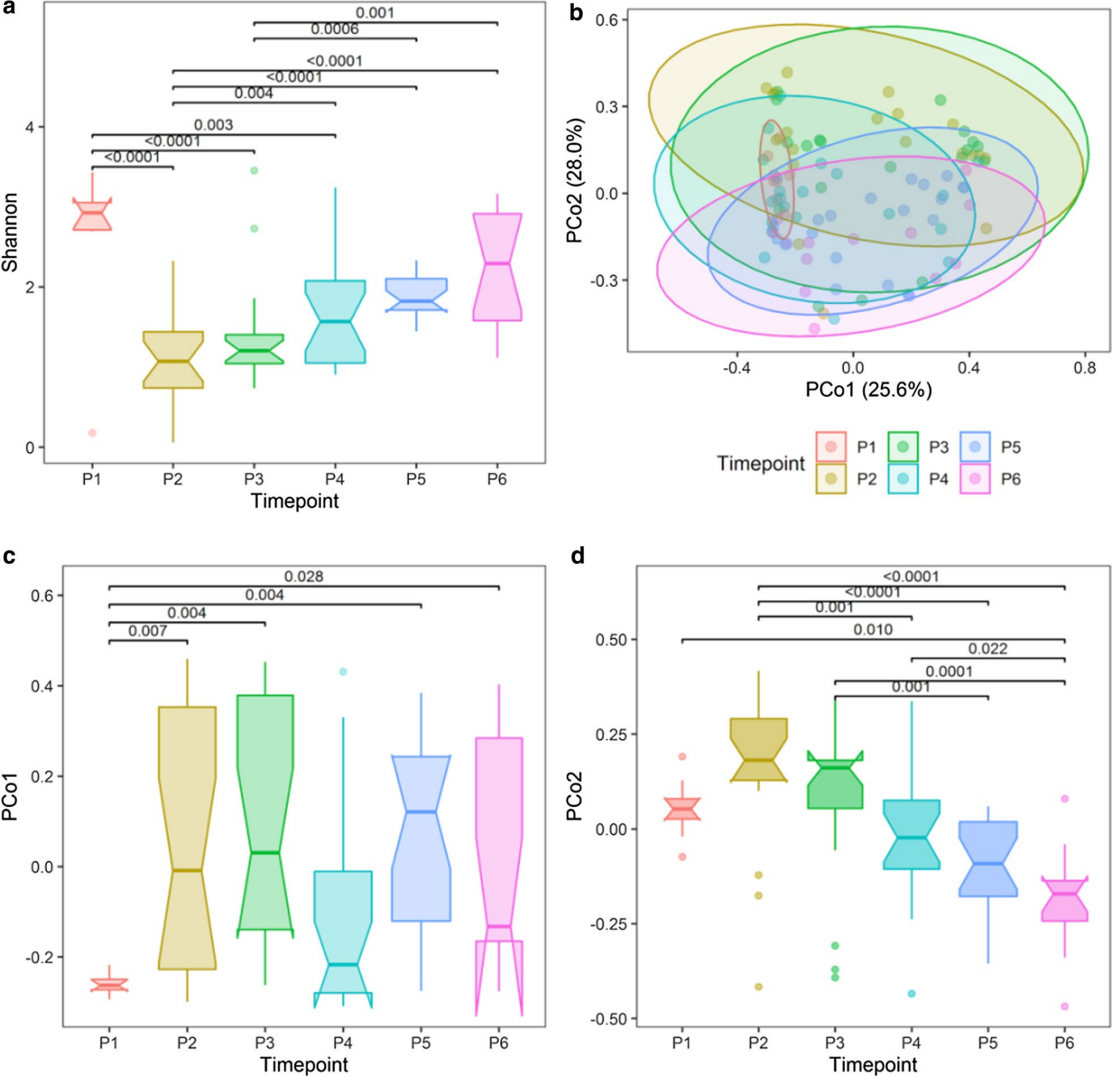
Gut microbiome: alpha and beta diversity over time. **a** Shannon alpha diversity by time, LRT, df = 5, P = 5.25e−09, adjusted for breastfeeding time and mode of delivery; **b** Principal coordinate analysis plot with Bray–Curtis dissimilarity calculated from genus abundances, ellipses were drawn assuming a multivariate t-distribution; **c** PCo1 scores by time, LRT, df = 5, P = 0.004; **d** PCo2 scores by time, LRT, df = 5, P = 5.19e−08; FDR adjusted p values < 0.05 are shown, P1-meconium, P2-7th day, P3-1st month, P4-6th month, P5-12th month, P6-24th month

The alpha diversity was measured using the Shannon index (Fig. 2a); sample diversity differed significantly between time points (LRT, df = 5, P = 6.61e−09). Specifically, P2′s alpha diversity was significantly lower than in P4, P5 and P6, whereas P3′s alpha diversity was significantly lower than in P5 and P6. We have validated those results by comparing diversity in stool samples with that in meconium in the two cohorts of children (PMU and HMS). In the PMU, the Shannon indexes in P2 (Q < 0.0001), P3 (Q = 0.0001), P4 (Q = 0.004) were significantly lower than that in P1; on the other hand, the Shannon indexes in P5 (Q = 0.109) and P6 (Q = 0.201) did not differ significantly from that. Altogether, these results indicate that the meconium samples have the highest alpha diversity; interestingly, our data suggest that the newborn’s gut microbiota diversity first decreases but then is restored over time (Fig. 2a). Importantly, a similar pattern was evident in the HMS cohort data (Additional file 1: Fig. 4a), except for a clear discrepancy between the first samples’ diversity (in the HMS cohort, the lowest diversity was detected at day 1, giving rise to a monotonically increasing trend in this cohort). This can occur if “high diversity” meconium samples (bacteria derived from the mother and environment following birth [36] are combined with early newborns’ stool “low diversity” samples (in the PMU cohort, only meconium stool samples were analyzed while in the HMS cohort also the first stool samples were analyzed in some cases).

The ordination of samples in a two-dimensional space performed as per a principal coordinate (PCo) analysis on Bray–Curtis distances dissimilarity based on genus abundance showed a sequential change in the gut microbial communities (Fig. 2b), especially along the PCo2 axis, where the transition of the gut microbial composition was smooth and gradual so that the differences in the PCo2 scores were significant only between more distant time points, i.e. P2–P4/P5/P6, P3–P5/P6 and P4–P6 (Fig. 2d). The meconium community composition was markedly dissimilar from those in later samples in both cohorts**.** This was especially evident along the PCo1 axis (Fig. 2c, Additional file 1: Figure S4C): in the PMU, the PCo1 scores were significantly different among all time points except for P4. While a global multivariate test for impact of time (partial Mantel test on Bray–Curtis dissimilarities relative to intersample time interval and donor incongruence matrices) does not achieve significance, we see significant (PCo1 scores by time, LRT, df = 5, P = 0.004; PCo2 scores by time, LRT, df = 5, P = 5.19e−08) differences between time points with regards to the first two principal coordinates of the gut taxonomic data, indicating an overall shift in sample composition over time nonetheless takes place (Fig. 2c, d). Altogether, these results align with previous findings regarding taxonomic composition of the meconium and fecal samples [25, 36], validating the microbial analysis methods used.

#### Gut microbiota composition and functional profiles during the first two years of life

**Fig. 3:**
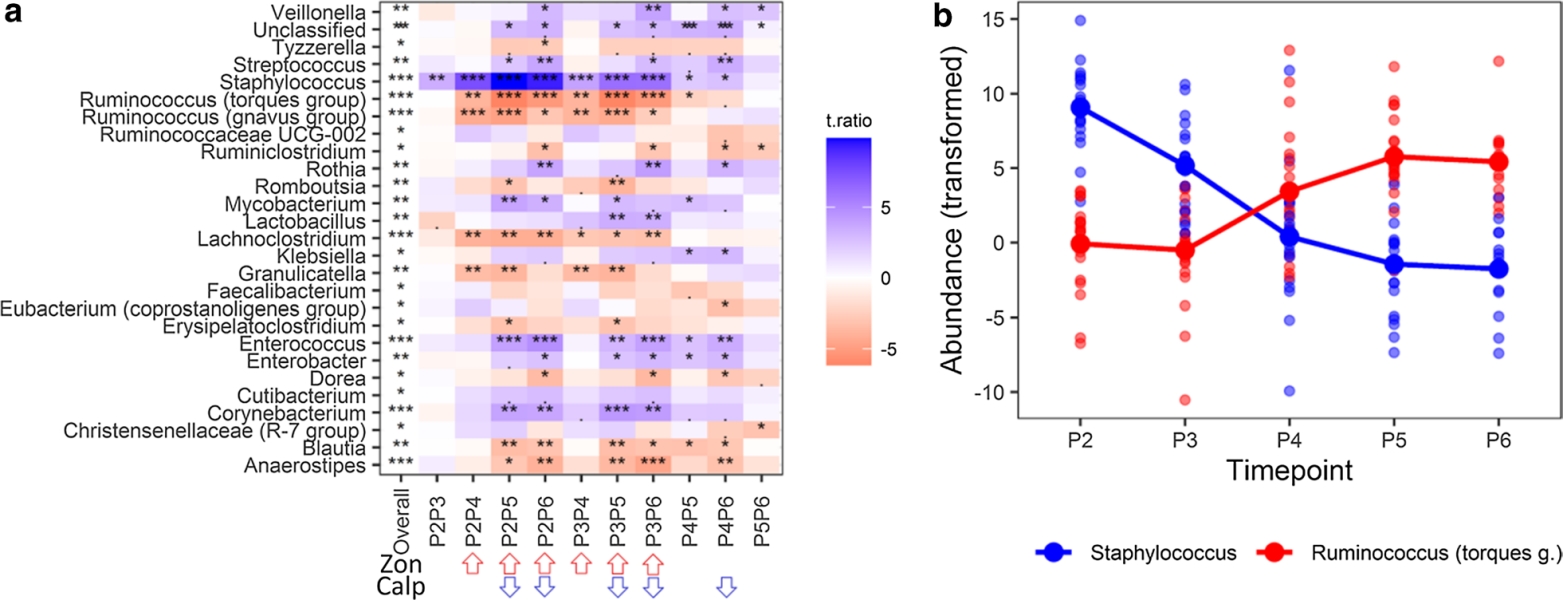
Changes in the gut microbiota composition during first two years of life. **a** linear mixed effects analysis followed by pairwise comparison of time- points (adjusted for mode of delivery and breastfeeding time), **a** Genera (present in at least 20% samples) significantly associated with time, **b** Longitudinal abundance of the *Staphylococcus* and *Ruminococcus* (*torques* group). The overall p-value—a likelihood ratio test (LRT) of nested models (FDR adjusted across genera); PXPY—contrast p values between the two time points (PX and PY), FDR adjusted for all possible contrasts, t.ratio—t statistics for the contrasts estimates (a positive value, colored blue, indicates a decrease in abundance, a negative value, colored red, indicates an increase in abundance). Red arrows indicate time intervals with a significant increase of zonulin, blue arrows indicate time intervals with a significant decrease of calprotectin (see also Fig. 1). Taxa abundances (unrarefied) were transformed by generating 128 Monte Carlo instances of the Dirichlet distribution for each gut sample, followed by center-log transform of each instance. A linear mixed effects analysis was performed for each instance separately and the results were averaged over 128 instances. P2**-**7th day**,** P3**-**1st month, P4-6th month**,** P5-12th month, P6-24th month

The bacterial taxonomic and functional (PICRUSt2-predicted) development during the first 2 years of life was analyzed using linear mixed-effect modeling of the centered log-ratio transformed 128 Monte Carlo instances of the Dirichlet distribution in samples from 7th day (P2) to the 24th month (P6) of age. Twenty-seven out of 47 evaluated genera that met the prevalence criterion either increased or decreased significantly during that period (Fig. 3a). Of note, the changes in the *Staphylococcus* and *Ruminococcus* (*torques* group) abundance were approximately monotonic throughout that time (Fig. 3b). In general, the abundance tended to stabilize around 1 year of life (P5) with only mild alterations thereafter (see the column P5P6 in Fig. 3a). Interestingly, except for *Staphylococcus*, the abundance was relatively constant until the end of the first month of life (P3, see the P2P3 column in Fig. 3a). Longitudinal changes in the bacterial composition at higher taxonomic ranks are shown in Additional file 1: Figure S5.

Remarkably, fifteen genera whose abundance changed significantly over time overlapped between the PMU and HMS cohorts (Fig. 3a, Additional file 1: Figure S6). This overlap accounted for 60.0% and 51.7% of the significantly affected taxa in the PMU (P2 to P6) and HMS (P2 to P5), respectively. Moreover, for overlapping genera, the patterns of temporal changes in abundance were similar (either decreasing or increasing); the degree of changes between time points also exhibited a similar pattern (i.e. P2P3, P2P4, P3P4).

To infer the functional profile of the microbial communities, we used the PICRUSt2 software. We identified 110 MetaCyc pathways (out of 332 predicted) in the PMU cohort that were significantly associated with time. In general, almost all pathways (except PWY-7332) did not change significantly in abundance after P5, and the majority of pathways stabilized even earlier at around the 6th month of age (P4). In the HMS cohort, the analysis of predicted MetaCyc pathways revealed 223 pathways (out of 290 predicted) whose abundance changed significantly over the observation period (P2 to P5). Importantly, there were 76 pathways whose abundance changed over time in the two cohorts (Additional file 1: Figure S7), accounting for 75.2% and 34.1% of the significant pathways in the PMU and HMS cohorts, respectively. Remarkably, there was a very high degree of consistency between the cohorts regarding the direction and dynamics of the pathways’ abundance.

### Gut microbiota diversity, composition and metabolic pathways concerning the stool levels of zonulin and calprotectin

Next, we focused on the relationships between microbiota diversity, composition, predicted function and permeability/inflammatory markers. In a first step, we assessed whether the time intervals with significant changes in bacterial diversity, abundance and predicted metabolic functions overlapped with the time intervals associated with an increase in zonulin (i.e. P2–P4/P5/P6, P3–P4/P5/P6) and decrease in calprotectin (i.e. P2–P5/P6, P3–P5/P6, P4–P6). Then, in a second step, we used a statistical technique (repeated measures correlation) that accounts for non-independence among observations comprising the entire observation period (from P2 to P6), to investigate the associations between the bacterial diversity, abundance and predicted metabolic functions and the concentration of zonulin or calprotectin. In a third step, mixed effects linear models were used to investigate the relationship between changes in bacterial diversity, abundance and metabolic function and the corresponding changes in the concentration of zonulin and calprotectin between all time point pairs.

The compatibility of observations obtained at all stages of the analysis was considered as potential evidence of a causal relationship between the microbiota and the markers analysed. To make this analysis easier accessible, these data are included in summary tables (Additional file 1: Table S5).

### Diversity

**Fig. 4:**
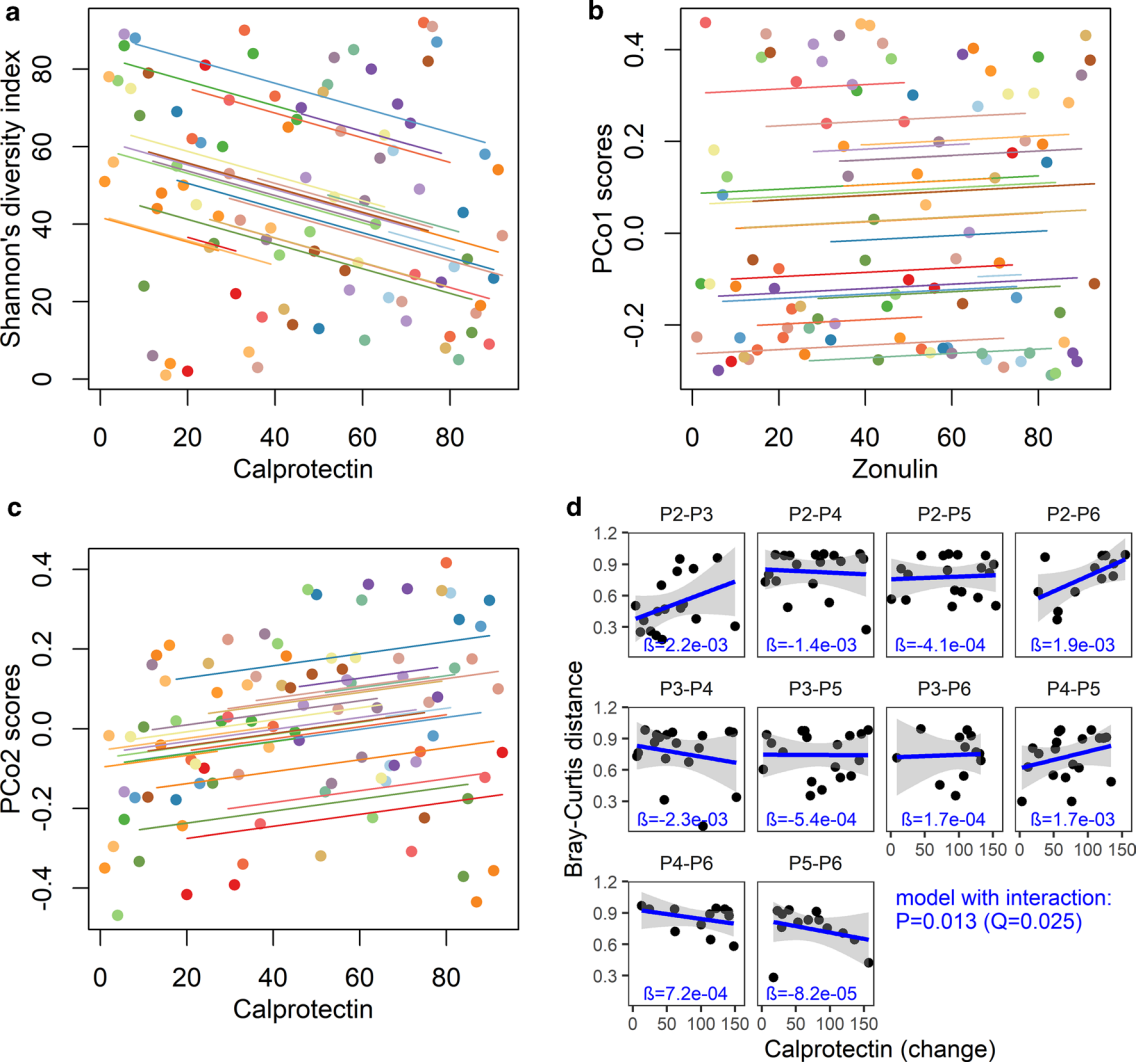
Alpha and beta diversity (Bray–Curtis distance) compared to zonulin and calprotectin. **a** Repeated measures correlation between calprotectin and Shannon diversity index. **b** Repeated measures correlation between zonulin and PCo1 scores, **c** Repeated measures correlation between calprotectin and PCo2 scores, **d** Correlation between Bray–Curtis distance and calprotectin change for all possible time point pairs. Significant likelihood ratio test (LRT) indicating an interaction between time period and calprotectin change (df = 9, P = 0.013, Q = 0.025), post-hoc test revealed significant correlations between Bray–Curtis distance and calprotectin change between P2 and P3 (β = 2.18e−03, P = 0.043) and P3 and P4 (β = − 2.31e−03, P = 0.011); alpha diversity, zonulin calprotectin and calprotectin change were rank transformed, repeated measures and correlation trend lines are coloured by individual child. PCo1 and PCo2 scores were treated here as a dimensionality-reduced measure of sample composition relative to whole-dataset variability

The Shannon index increased significantly in five out of six time intervals associated with a significant increase in zonulin (i.e. P2–P4/P5/P6, P3–P5/P6, Figs. 1a, 2a) and in four out of five time intervals associated with a significant decline in calprotectin (P2–P5/P6, P3–P5/P6, Figs. 1b, 2a). To investigate whether or not a relationship exists between the alpha diversity and stool zonulin/calprotectin levels, we performed correlation analysis between four alpha-diversity indices (Shannon, Simpson, Chao1, Evenness) and the levels of these biomarkers using repeated measures correlation. The zonulin levels did not correlate with any of the alpha diversity indices (after FDR adjustment). In contrast, the levels of calprotectin inversely correlated with all of the alpha diversity indices calculated (Shannon’s-r = − 0.30, Q = 0.039; Simpson’s-r = − 0.23, Q = 0.048, Chao1-r = − 0.24, Q = 0.048, Evenness-r = − 0.24, Q = 0.048); of note, the strongest correlation was found for the Shannon’s diversity (Fig. 4a).

The PCo2 scores decreased significantly in five out of the six time intervals associated with a significant rise in zonulin (P2–P4/P5/P6, P3–P5/P6, Figs. 1a, 2d) and in all five time intervals associated with a significant decrease in calprotectin (P2–P5/P6, P3–P5/P6, P4–P6, Figs. 1b, 2d). Besides, there was some similarity between the longitudinal zonulin and PCo1 scores pattern (Figs. 1a, 2c). Therefore, we explored the correlations between the zonulin and calprotectin levels and the per-sample positions on the first two PCoA axes using repeated measures correlation analysis. We did not find any significant correlation (zonulin and PCo1: r = 0.06, P = 0.637; zonulin and PCo2: r = − 0.23, P = 0.053; calprotectin and PCo1: r = 0.11, P = 0.363; calprotectin and PCo2: r = 0.19, P = 0.109) suggesting that the overall compositional profiles (captured by the first two PCo dimensions) were not associated with the levels of zonulin or calprotectin (Fig. 4b, c). Moreover, using likelihood ratio tests of the nested models, no association the between Bray–Curtis distances and the changes in the zonulin levels was found (non-interaction model: df = 1, P = 0.121, Q = 0.241; interaction model: df = 9, P = 0.965, Q = 0.965). However, there was a significant association between the Bray–Curtis distances and the changes in calprotectin levels (time period x calprotectin change interaction model: df = 9, P = 0.013, Q = 0.025). The post-hoc tests revealed significant correlations between the changes in calprotectin levels and the Bray–Curtis distances for P2–P3 and P3–P4; however, they were not significant after FDR adjustment (Q = 0.216 and Q = 0.110, respectively, Fig. 4d).

#### Microbiota composition and functional profiles concerning stool zonulin and calprotectin

Despite the lack of associations between alpha diversity and the zonulin levels as well as between beta diversity and the levels of both biomarkers (zonulin and calprotectin) (Fig. 4), we sought to examine more particular relationships between the levels of these permeability/inflammation-associated biomarkers and the abundances of specific gut bacterial taxa in the context of five taxonomic ranks (from genus to phylum levels) as well as the PICRUSt2-predicted MetaCyc metabolic pathways.

**Fig. 5:**
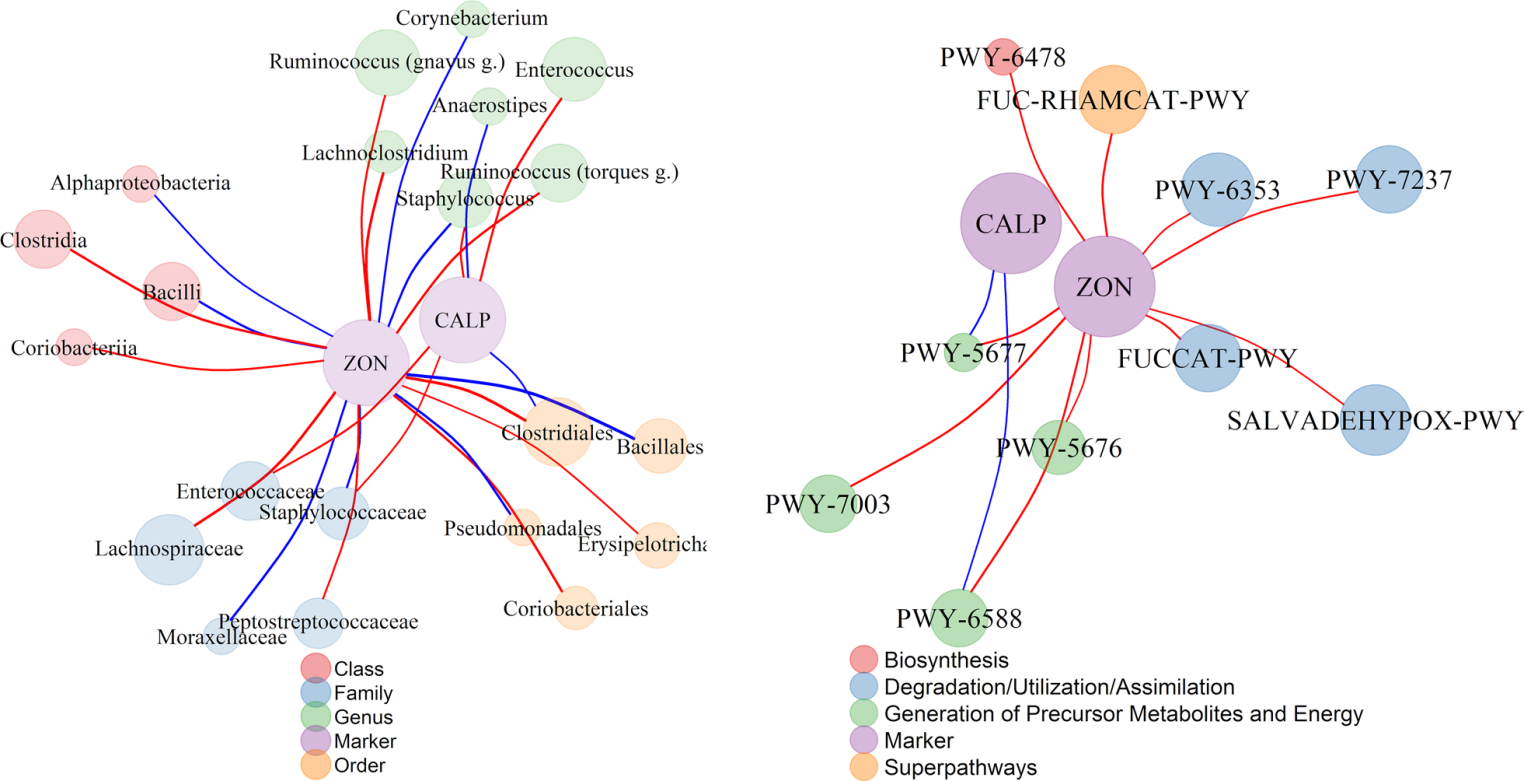
Significant repeated measures correlations of zonulin and calprotectin with microorganisms and PICRUSt2 predicted MetaCyc pathways. **a** taxon correlation network, **b** MetaCyc pathway correlation network node size and edge width are proportional to microorganisms/pathways abundance and repeated measure correlation coefficients, respectively; line color indicates the negative (blue) and positive (red) correlations, node color indicates taxonomic rank or metabolic pathway categories. Taxonomic abundances (unrarefied) were transformed by generating 128 Monte Carlo instances of the Dirichlet distribution for each gut sample, followed by center-log transform of each instance

Interestingly, the changes in abundance of several bacteria coincided with significant time dependent changes in the levels of zonulin or calprotectin (Figs. 1, 3a). For example, there was an increase in the abundance of *Lachnoclostridium*, *Ruminococcus* (*gnavus* group), Carnobacteriaceae, Lachnospiraceae, Peptostreptococcaceae, Coriobacteriales, and a decrease of Corynebacteriales among all time points associated with an increase in the zonulin concentration. Additionally, there was an increase in the abundance of *Anaerostipes* and a decrease in the abundance of Enterococcaceae among all time points associated with a decrease in calprotectin concentration. To investigate this further, we computed the repeated measures correlations (encompassing the whole study period; Fig. 5 and Additional file 1: Table S5) between the levels of both markers and the gut microorganisms.

In the correlations between gut microorganisms and stool calprotectin, we observed significant associations at the genus level (n = 3), the family level (n = 3), and the order level (n = 1). Moreover, the levels of zonulin were correlated with the abundance of 19 microorganisms: at the genus level (n = 5), at the family level (n = 5), at the order level (n = 5) and the class level (n = 4) (Fig. 5a and Additional file 1: Table S1). Specifically, calprotectin correlated positively with *Staphylococcus*, *Enterococcus,* Staphylococcacae, and Enterococcaceae and negatively with *Anaerostipes*, Ruminococcacea and Clostridiales, whereas zonulin correlated positively with *Lachnoclostridium*, the *Ruminococcus (gnavus* group), the *Ruminococcus (torques* group), Lachnospiraceae, Peptostreptococcaceae, Ruminococcacea, Erysipelotrichales, Coriobacteriales, Clostridiales, Clostridia, Coriobacteria and negatively with *Staphylococcus, Corynebacterium*, Moraxellaceae, Staphylococcaceae, Bacillales, Pseudomonadales, Alphaproteobacteria, and Bacilli. Of note, the genus *Staphylococcus*, the families Ruminococcaceae and Staphylococcaceae, and the order Clostridiales were simultaneously and oppositely correlated with both biomarkers in the PMU cohort (Fig. 5a) and with calprotectin in the HMS cohort, exhibiting the same direction and similar magnitudes as those in the PMU cohort (Additional file 1: Table S2). Additionally, zonulin correlated significantly with 10 MetCyc pathways belonging to 4 superclasses (Superpathways: FUC-RHAMCAT-PWY, Degradation/Utilization/Assimilation: FUCCAT-PWY, PWY-6353, PWY-7237, SALVADEHYPOX-PWY, Generation of Precursor Metabolites and Energy: PWY-5676, PWY-5677, PWY-6588, PWY-7003, and Biosynthesis: PWY-6478), of which two pathways, PWY-5677 and PWY-6588, were the only pathways associated with calprotectin (Fig. 5b). The correlation between calprotectin and predicted metabolic pathways from the HMS cohort revealed 15 pathways significantly correlating with calprotectin levels; however, none of them overlapped with the correlations found in the PMU cohort (Additional file 1: Table S2).

**Fig. 6:**
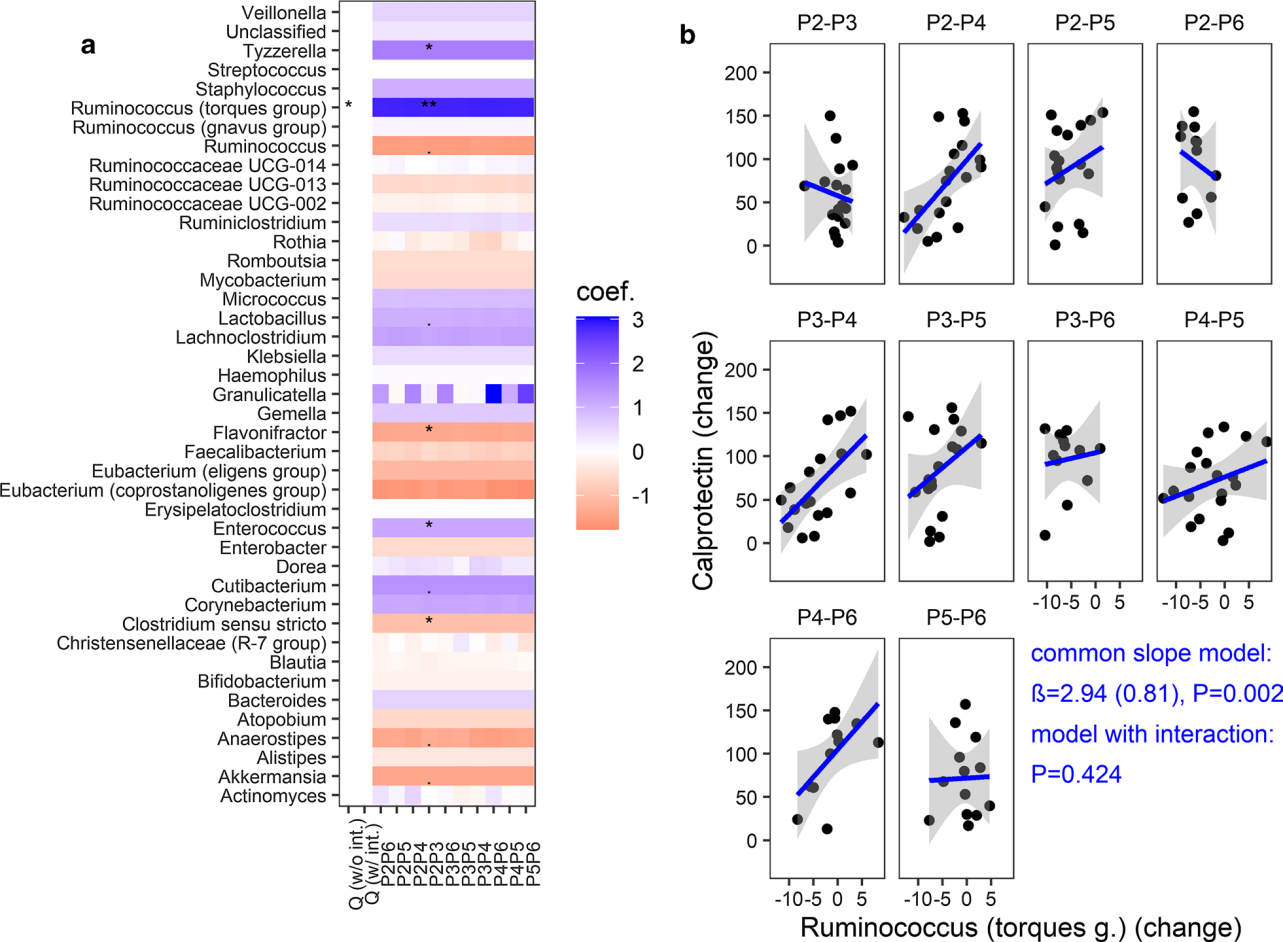
Relationship between gut genus abundance and calprotectin levels (changes per time interval). **a** Linear mixed effects models were used to test for an association between the taxon abundance change and calprotectin change accounting for ten time point pairs (P2–P3, P3–P4, etc.) from the same subject**,** adjusted for mode of delivery and breastfeeding time. Two models were considered: without interaction (w/o int.) and with interaction between time point pair and taxon change (w/ int.). If the interaction term was significant—Q (w/ int.) < 0.05, individual p values (FDR adjusted) were interpreted. If the common slope model was chosen—Q (w/o int.) < 0.05 and Q (w /int) > 0.05, the same common slope coefficient β (coef.) across all time points and one p-value (P2–P3) were shown. Coefficients and Q values were averaged over 128 Monte Carlo instances of the Dirichlet distribution, followed by center-log transform of each instance, **b** the common slope model (LRT, df = 1, P = 0.002, Q = 0.015) illustrating relationships between *Ruminococcus (torques* group) change (centered log-ratio transformed) and calprotectin change (rank transformed)—based on the first Monte Carlo instance

In order to gain more insight into the causal relationship between the microbiota and zonulin/calprotectin, the changes in the gut community features (taxonomic composition and functional profile) and the changes in the levels of zonulin or calprotectin among the time points were correlated using a linear mixed-effects model. We did not find any association between changes in microbiota and changes in markers, except for the genus *Ruminococcus* (*torques* group) which was significantly associated with the changes in levels of calprotectin. he interaction model did not fit significantly better than the common (positive) slope model (Fig. 6a, Table 2). Thus, the abundance of *Ruminococcus* (*torques* group) changes in parallel with the changes in stool calprotectin levels regardless of time interval (which is especially prominent for the P2–P4, P2–P5, P3–P4, P4–P6 time intervals), thereby suggesting that both can be causatively connected. However, this observation was not confirmed in the validation analysis and must be taken with caution. The analysis in the context of higher taxonomic ranks for the PMU and HMS cohorts are shown in the Additional file 1: Figures S8–S10.

**Table 2:** Association of the marker change (zonulin, calprotecin) with taxon/pathway change—a linear mixed model

Taxon/Pathway vs marker (changes)	Common slope (no interaction)			Interaction (taxon/pathway change x time interval)	
	β (SE)	P	Q	P	Q
*Ruminococcus* (*torques* group) vs calprotectin	2.94 (0.81)	0.002	0.015	0.424	0.910
CENTFERM-PWY vs calprotectin	− 4.54 (1.08)	0.0002	0.028	0.412	0.993
PWY-6590 vs calprotectin	− 4.48 (1.16)	0.0002	0.026	0.421	0.993
PENTOSE-P-PWY vs zonulin	9.49 (1.93)	3.98e−6	0.001	0.123	0.356
PWY-6545 vs zonulin	Na	–	–	4.15e-5	0.014
PWY-6901vs zonulin	Na	–	–	0.0005	0.046
PWY0-845 vs zonulin	Na	–	–	0.0008	0.049

β, coefficient estimate; SE, standard error; Q -FDR adjusted P value, Na, not applicable

**Fig. 7:**
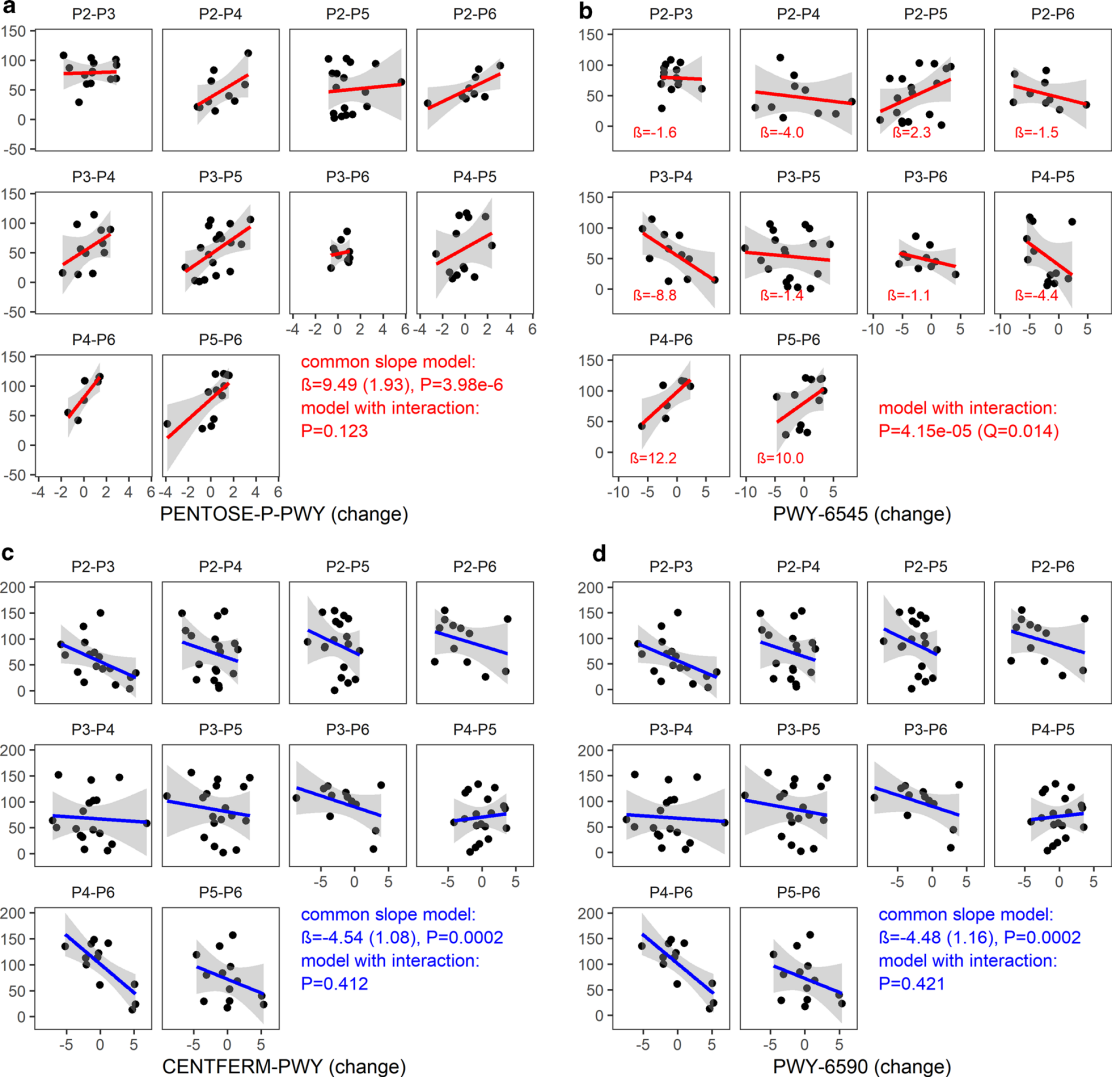
Relationship between (changes in) gut abundance of inferred MetaCyc pathways and (changes in) zonulin/calprotectin levels. **a** The common slope model (LRT, df = 1, P = 3.98e−6, Q = 0.001) illustrating relationships between PENTOSE-P-PWY change (centered log-ratio transformed) and zonulin change (rank transformed), **b** the model with time point pair by PWY-6545 change interaction (LRT, df = 9, P = 4.15e−5, Q = 0.014), **c** common slope model illustrating relationships between CENTFERM-PWY change (centered log-ratio transformed) and calprotectin change (rank transformed), **d** common slope model illustrating relationships between PWY-6590 change (centered log-ratio transformed) and calprotectin change (rank transformed); Likelihood ratio test (LRT) P- and Q-values were computed based on 128 Monte Carlo instances of the Dirichlet distribution, followed by center-log transform of each instance, the plots were based on the first Monte Carlo instance

In addition to the changes in the composition of the human gut microbiota, we also investigated whether the changes in functional profiles of gut microbiota were linked to the dynamics of the zonulin or calprotectin levels. Remarkable, we found that the dynamics of stool calprotectin was negatively associated with changes in two MetaCyc metabolic pathways: pyruvate fermentation to butanoate (CENTFERM-PWY) and the superpathway of *Clostridium acetobutylicum* acidogenic fermentation (PWY-6590) (Fig. 7c, d). The common slope model was the best fit for these pathways (Table 2). For zonulin, the common slope model was the best fit for a change in the pentose phosphate pathway (PENTOSE-P-PWY) only Fig. 7a, Table 2). In addition, the dynamics of zonulin was associated with changes in 3 MetaCyc pathways: pyrimidine deoxyribonucleotides de novo biosynthesis III (PWY-6545), the superpathway of glucose and xylose degradation (PWY-6901), and the super pathway of pyridoxal 5′-phosphate biosynthesis and salvage (PWY0-845). For those 3 pathways, as illustrated in Fig. 7b for PWY-6545, the models with different slopes (i.e. including an interaction between all time point pairs and pathway changes) fitted the data significantly better than the common slope model. For example, the change in zonulin levels was associated negatively with a change in PWY-6545 (Fig. 7b, Table 2) between P3 and P4 (P = 0.001, Q = 0.012), yet positively between P4 and P6 (P = 0.003, Q = 0.012) as well as P5 and P6 (P = 0.004, Q = 0.012). For the other two pathways, PWY-6901 and PWY0-845 (Table 2), significant associations were found between P3 and P4 (β = 23.60, SE = 7.35, P = 0.002, Q = 0.008) as well as P5 and P6 (β = 36.08, SE = 10.56, P = 0.0008, Q = 0.008) for PWY-6901, and between P3 and P4 for the PWY0-845 (β = 13.07, SE = 3.66, P = 0.0005, Q = 0.005). For the full results, see Additional file 1: Tables S3 and S4.

However, it should be noted that the above results (concerning calprotectin) were not confirmed in the HMS cohort: 44 pathways showed a correlation with the dynamics of calprotectin (all under the common slope model), but the CENTFERM-PWY (P = 0.051, Q = 0.131) and PWY-6590 (P = 0.056, Q = 0.140) did not (Additional file 1: Figure S11). The relationships between the changes in abundance in the gut, the MetaCyc pathways, and the changes in calprotectin levels in the HMS cohort are shown in Additional file 1: Figure S12.

## Discussion

This is the first study in which putative associations between the gut microbiota and the concentrations of zonulin and calprotectin in children's stool during the first 2 years of life have been investigated. The study of zonulin allows the non-invasive assessment of the functional state of the small intestinal paracellular permeability; additionally, the calprotectin measurements provide insight into the development of the immune system after birth. The observed changes of both markers concentration indicate that the 6th month of life can be defined as the key point for forming the small intestinal barrier and for the development of postnatal immunity. It should also be stressed that, usually, in the sixth month of life, solid food is introduced to children’s diet, which can significantly impact both gut microbiota and intestinal permeability.

Although zonulin seems to be a valuable marker of small intestinal paracellular permeability, the data in the context of children aged 2 years old or younger are scarce and focused on infections [37] and prematurity [38]. The fecal zonulin concentration in children during the first 2 years of life was not reported and its role during this period is unknown. Its relationship with gut microbiota seems to be interesting [38]. Zonulin is involved in controlling the passage of molecules weighing at least 3.5 kDa [39] through the intestinal barrier via its reverse influence on TJ tightness [40, 41]. After the activation of ZO-1, zonulin controls the low-capacity “leak” type route characterized by low selectivity [42, 43]. Interactions between zonulin and gut microbiota can be bidirectional. Bacterial and gluten exposure was defined as intensive triggers of zonulin release [19, 44]. Of note, the zonulin pathway is an innate defensive mechanism of the host, able to control the gut microbiome composition via the “flushing out” of microorganisms by water secreted into the intestinal lumen following hydrostatic pressure gradients [45]. High stool zonulin levels suggest that the gut barrier allows the free flow exchange of various particles in infants. Moreover, it is likely that the commercially available ELISAs detect one or more members of the zonulin family that have not been discovered yet but play some important role during the first 2 years of life [46].

High levels of calprotectin in feces during the first months of life are associated with the regulation of the development of the immune system in neonates as well as with the adaptation to new environmental conditions [25]. The decrease in the fecal calprotectin concentration implies that inflammatory processes in the gut tend to decrease from the 6th month of age, which, with the accompanying increased paracellular intestinal permeability and bacterial alpha diversity, provides suggestion of immune tolerance. Willers et al. [25] demonstrated that mice exposed to calprotectin immediately after birth induced microbial tolerance against the first wave of microbial colonization; on the other hand, the same exposure after the neonatal period was associated with pro-inflammatory responses. Wood et al. observed an increased proportion of regulatory T cells (Tregs) during the first three weeks of life [47] which is associated with increased immune tolerance after the birth. Our results did not fully corroborate these observations. The intestinal permeability in the first month of life, as per the zonulin concentration, is lower than that in the later period, suggesting that the possibility of antigen translocation early after birth is limited. After the sixth month of age, the intestinal paracellular permeability increases, but this does not increase inflammation, as per the calprotectin concentration, which indicates the development of immune tolerance. The differences between the two studies may be due to the fact that Willers et al. did not directly measure intestinal permeability and additionally used an animal model that does not closely mimic the timing of human intestinal barrier development and Wood et al. measured Treg proportion only during the first three weeks of life however. As such, direct comparison of immune tolerance as reported by them and as per our present study is not possible, and we look forward to future studies bridging this gap. Moreover the role of paracellular way in translocation of bacteria and endotoxin is still the subject of debate [22], what requires that the observed results should be interpreted with caution.

In summary, the increased production of zonulin may be caused by changes in intestinal bacteria and by the introduction of gliadin into the diet. In contrast, the decrease in the calprotectin content in the stool may indicate immunological tolerance development. However, the breast milk is an essential source of calprotectin, which can significantly influence the fecal calprotectin content. This said the observed changes cannot be explained solely by environmental factors as zonulin decreases in the later years of life despite the constant consumption of gliadin. Therefore, in the context of longitudinal analyses of biomarker trajectories, we applied corrections for the time of breastfeeding and the type of delivery to minimize the influence of these confounders. To help resolve the mechanisms underlying our results, we decided to analyze whether a cause-effect relationship between the microbiota and the biomarkers would be observed. Of note, the microbial changes in the first 2 years of life are very dynamic and depend on the age, mode of delivery, feeding, antibiotic treatment, and other environmental factors [28, 29]. However, a comprehensive analysis of all factors influencing the gut microbiota is beyond the scope of this study, which is focused on the relationship between the microbiota and the fecal zonulin and calprotectin content. This said the compositional and functional comparison of the PMU and HMS cohorts' microbiota would be very interesting.

The Shannon index (more sensitive to species richness), shared 5 and 4 significant time intervals with zonulin (increasing) and calprotectin (decreasing), respectively. Similar results were observed for coordinates of the first two axes in the context of a principal coordinate analysis based on the Bray–Curtis distance, one of beta-diversity measures. However, it translated only into a negative correlation between alpha-diversity indices, the Shannon index in particular, and calprotectin. This negative correlation may be associated with the development of immunological tolerance. The microbial colonization of the intestine after birth will generate a diversity of new antigens that will play an important role in the stimulating of epithelial function and the establishing the offspring’s immune system [48]. Of note, weaning (the average breastfeeding time in our study was about 30 weeks), and the resulting loss of milk-borne calprotectin, sIgA and other anti-microbial factors, along with the transition from mothers’ milk to a complex diet (including solid food), can have a major impact on the dynamic microbiota development, and thus in the resulting immune responses during the neonatal period [6].

Willers et al. [25] reported that the gut microbiota's overall diversity significantly increases during the first year of life, similarly to what we observed in this study. Moreover, they observed the increased abundance of the bacterial classes Actinobacteria, Bacteroidia [49–51] and Clostridia, along with the decreased abundance of Bacilli and Gammaproteobacteria over the same time period. Of note, Willers et al. [25] demonstrated that during infancy, high abundance of Actinobacteria and low abundance of Gammaproteobacteria are linked to high fecal calprotectin levels; additionally, at the family level, fecal calprotectin promoted the higher abundance of Bifidobacteriaceae and the reduction of Enterobacteriaceae via the production of acetate [52]. Taxonomic composition observed by Willers et al. [25] might be linked to the elevation in the synthesis and reduction in the degradation of SCFAs and therefore leading to the overrepresentation of health-promoting gut microbiota metabolic functions. Our results confirm the observations obtained by Willers et al. [25] in terms of abundance; however, the correlations in the cited study were not reproduced here. Importantly, the observed differences may depend on the difference between the investigated populations (Polish and German) or/and the analytical methods used. This said the analysis of both data-sets showed similarities with respect to the correlation between the levels of calprotectin and bacteria at the family level.

Interestingly, the bacteria associated with zonulin and calprotectin (Table 3) may be involved in the production of SCFAs, which is a protective factor in the context of intestinal barrier integrity and of inflammatory processes. Particularly, the different taxonomic groups of bacteria within the class Clostridia were associated with increased zonulin and decreased calprotectin concentrations. Within this class, the family Ruminococcaceae was more abundant in women with low zonulin concentrations in a previous study; however, here, we observed the opposite pattern. Importantly, not all bacteria within this class have the same properties. For instance, the genus *Ruminococcus* (*gnavus* group)—positively correlated with fecal zonulin concentration, is increased in patients with IBS-D and Crohn’s disease, which suggests its pro-inflammatory activity. An interesting result was obtained in the case of the *Ruminococcus* (*torques* group). *Ruminococcus torques*, a butyrate producer, have been found to dominate patients' gut milieu with Crohn’s Disease [53, 54]. They were also studied in the context of individuals with increased risk of upper gastrointestinal tract involvement, but with inconclusive results [55–57]. Importantly, *Ruminococcus torques* are able to utilize mucin in the human intestine, sustaining their adaptability to the human intestinal environment [54, 58, 59]. Consequently, increased mucin degradation makes luminal antigens cross the gut barrier and stimulate the immune system, leading to intestinal disorders [60]. Of note, in this study, the abundance of these above bacteria positively correlated with the concentration zonulin and the dynamics of calprotectin concentration. Therefore, bacteria from the *Ruminococcus* (*torques* group) may include species associated with small intestinal permeability and immune responses in the first 2 years of life. For deeper species resolution and assessment of the gut functional potential future shotgun metagenomics, metabolomics.

**Table 3:** Levels of associations between bacteria and markers

Bacteria (taxonomic affiliation)	Change^a^		RMC	LME	Bacteria properties
	ZON	CALP			
Class Alphaproteobacteria			Zon (−)		Exhibited numerous positive co-occurrences with other microbes [71]; enriched in vaginally delivered standard formula-fed infants three months post delivery [72]; high abundance in placentas of women with IBD, but not in stool [73]; high abundance in patients with Crohn's Disease [74]; third major constituent gut microbiome in infants with respiratory and gastrointestinal diseases [75]
Class Clostridia			Zon (+)		Dominates in infants [76]; in the formula-fed infants colonization occurred consistently throughout the 1st year of life, whereas in some breast-fed infants it was inhibited until weaning [77]; anti-inflammatory activity [78–80]
Order Clostridiales (class Clostridia)			Calp (−) Zon ( +)		Butyrate production [81, 82]; suppression of proinflammatory bacteria [83]; protective role of the taxa in IBD pathogenesis [84]; induction an immune response [67]
Family Ruminococcaceae (class Clostridia)			Calp (−) Zon (+)		Ruminococcaceae is more abundant in women with low serum zonulin concentration and can enhance intestinal barrier integrity [85]; involved in modulation of gut barrier function[86]; SCFAs producer [87]; more abundant in people with diets high in carbohydrates [88, 89]
Genus *Ruminococcus (gnavus* group) (family Ruminococcaceae, order Clostridiales, class Clostridia)	Same		Zon (+)		Increased in patients with IBS-D [90] and Crohn’s Disease [91]
Genus *Ruminococcus (torques* group) (family Ruminococcaceae, order Clostridiales, class Clostridia)			Zon (+)	Calp (common slope model, positive β coeff.)	Butyrate production, important role in Crohn’s Disease [91]; mucin degradation [54]
Family Lachnospiraceae (order Clostridiales, class Clostridia)	Same		Zon (+)		Butyrate producer [92] induces the expression of Treg—suppression of the colonic inflammatory response [62]
Genus *Lachnoclostridium* (family Lachnospiraceae, order Clostridiales, class Clostridia)	Same		Zon (+)		Enhanced the utilization of carbohydrates [93] showed a positive association with secondary bile acids in mice [94]; SCFAs producer [95]; correlated with vitamin B6 metabolism and tryptophan metabolism in mice with colitis [96]; lower abundances was observed in neoplasms of gastrointestinal tract [97]; decreased in Autism Spectrum Disorders [98]; overdominant in population of native Alaska with high incidence of sporadic colorectal cancer [99]; positively correlated with body mass index, alanine aminotransferase, aspartate aminotransferase levels in patients with non-alcoholic fatty liver disease (NAFLD) [100]
Genus *Anaerostipes* (family Lachnospiraceae, order Clostridiales, class Clostridia)		Oppo-site	Calp (−)		Butyrate producer [101]
Family Peptostreptococcaceae (order Clostridiales, class Clostridia)	Same		Zon (+)		Overrepresented in gut microbiota of non-breastfed infants [102]; present in infants fed with standard cow’s milk formula [103]; overrepresented in infants living with pets and underrepresented in infants living with older siblings [104]
Class Bacilli			Zon (−)		Dominant in duodenum and in the jejunum; producer of enzymes, bile acid hydrolases, ACE-like inhibitors, SCFAs, hydrogen peroxide, group B vitamins, beneficial for gut health [105]; participate in metabolism of dietary components, xenobiotics and drugs helping to maintain intestinal homeostasis and host health [106, 107]
Order Bacillales (class Bacilli)			Zon (−)		Enriched in preterm infants fed with mom’s own milk [108]; overrepresented in cesarean section delivered neonates [109]; second major microbiome constituent of neonatal intensive care unit rooms [110]
Family Staphylococcaceae (order Bacillales, class Bacilli)			Calp (+) Zon (−)		Present in human milk [111]
Genus *Staphylococcus* (family Staphylococcaceae, order Bacillales, class Bacilli)			Calp (+) Zon (−)		One of the most prominent bacteria of human milk [112]; correlated with higher fecal calprotectin concentration in infants [113]; pathological bacteria decreased over time after delivery [114], super antigen function stimulates the systemic secretion of IgA in neonates, protecting against allergies [115]; promotes the modulation of the infant immune system without causing an adverse inflammatory response, induction of T-reg cells via butyric acid and propionic acid [116]; participate in the saccharolytic fermentation of carbohydrates, which end products that positively affect host cells and gut bacterial community [117]
Family Enterococcaceae (class Bacilli)		Same	Calp (+)		Opportunistic pathogen [118]; potential biomarker for IBD [119]
Genus *Enterococcus* (family Enterococcaceae, class Bacilli)			Calp (+)		Higher counts in premature infants with necrotizing enterocolitis (NEC) [120]; significant positive correlations between fecal calprotectin levels and intestinal colonization levels Enterococcus in Preterm Infants during the Neonatal Period [17]; more abundant in feces of formula fed infants [121]; a leading hospital-associated pathogen [122]
Family Carnobacteriaceae (class Bacilli)	Same				potential biomarker of autoimmunological disease [123]; abundance in oral microbiome was associated with decreased risk of colorectal cancer [124]
Class Coriobacteriia			Zon (+)		Greater prevalence in formula-fed babies [125]; significantly higher in coeliac infants [126]; lower relative abundances in atopic dermatitis children [127]; increased in Crohn’s Disese [128]
Order Coriobacteriales (class Coriobacteria)	Same		Zon (+)		Negatively associated with fecal protease activity associated with various gastrointestinal tract diseases [129]; can be considered as pathobionts, because their occurrence has been associated with a range of pathologies such as bacteremia, periodontitis, and vaginosis [130]; belongs to class Actinobacteria which correlates with fecal calprotectin [25]
Order Corynebacteriales	Oppo-site				Subset is pathogen [131]; belongs to class Actinobacteria which correlates with fecal calprotectin [25]
Genus *Corynebacterium* 1 (order Corynebacteriales)			Zon (−)		Human skin bacteria, dominant in gut microbiome of Cesarean Section (CS) born infants [132]; counts in gut microbiome of CS born children negatively associated with maternal dairy intake [133]; present in Meconium of Preterm Neonates [134]; dominant in oral microbiome of CS born infants [135]
Order Erysipelotrichales			Zon (+)		More abundant in infants at 4 weeks fed with cow-milk based formula, at 26 weeks in breast milk fed children and those receiving fructooligosaccharides (FOS) [103]; decreased in new-onset Crohn’s disease [56]; lower abundance in blood of patients with cirrhosis [136]; increased in HIV infection [137]
Order Pseudomonadales			Zon (−)		Associated with normal calprotectin level in Gambian infants [138]; belongs to Gammaproteobacteria negatively correlates with calprotectin [25]; associated with neutrophilia and lower vaccine responses in infants [139]; pathogenic taxa [140]
Family Moraxellaceae			Zon (−)		Dominant phylum in human breast milk [141]; overrepresented in Crohn’s disease closely associated with microaerobic energy metabolism, amino acid degradation, and energy deficiency characterized by low ATP levels [142]; lowered in colorectal cancer gut microbiome [143]; belongs to class Gammaproteobacteria which is negatively associated with calprotectin concentration [25]

^a^co-occurrence between change in the abundance and change in zonulin (P2–P4/P5/P6, P3–P4/P5/P6) or calprotectin (P2–P5/P6, P3–P5/P6, P4–P6) levels: same—a change in the bacterial abundance and change in marker level between time points occur in the same direction, opposite—a change in the bacterial abundance and change in marker level between time points occur in the opposite direction

RMC (repeated measures correlation)—correlation between the bacterial abundance and concentration of zonulin/calprotectin for paired measures assessed on six occasions (from P1 to P6, i.e., the entire observation period); LME (linear mixed-effects analysis)—an association between the taxon abundance change and marker change accounting for multiple time point pairs from the same subject, including time-point pair-specific relationships with either absence (the common slope model) or presence of the interaction (the different slopes model) between the abundance change and the time point pair); ( +), positive RMC coefficient; (−), negative RMC coefficient

It is not possible to draw definitive conclusions regarding cause-effect relationships in a descriptive study like this, especially not given limited power due to sample size (see Methods). Moreover, we cannot exclude observed relationships could reflect hidden shared relationships with other covarying factors. However, as the association between calprotectin and *Ruminococcus* abundance remained statistically significantly also accounting for age and delivery mode under stringent FDR adjustment, we can likewise conclude it is not reducible to the role of these factors, but rather constitutes an integral part of the system, with findings and implications beyond the age/maturity aspect. Details of the associations between bacteria and markers and bacteria properties are presented in Table 3. Only some of the relationships are discussed below. In this study, the Bacilli class correlated with decreased zonulin and increased calprotectin levels. In general, this class is considered beneficial for gut health, which is in line with decreased zonulin levels. On the other hand, the family Staphylococcaceae and genus *Staphylococcus* from the Bacilli class are the most prominent bacteria in human milk, an important calprotectin source in the infant. Family Enterococcaceae belongs to opportunistic pathogens, considered as potential marker for IBD, what can explain correlation with increased fecal calprotectin concentration. However, our present results do not allow consistent conclusions on the role of bacteria in the small intestinal barrier's permeability or the immune system in the first 2 years of life. For example, the *Lachnoclostridium* genus produces SCFAs and occurs in lower amounts in patients with gastrointestinal cancers; therefore, in theory, it should decrease intestinal permeability; however, it was correlated with increased concentrations of zonulin in the neonatal stool in this study. Alphaproteobacteria occur in high numbers in patients with IBD and are negatively associated with the concentration of zonulin. The order Corynebacteriales is associated with decreased zonulin and belongs to the class Actinobacteria, which was positively correlated with calprotectin in the study by Willers et al. [25]. The order Pseudomonadales and the family Moraxellaceae are negatively correlated with zonulin and belong to Gammaproteobacteria, negatively correlated with calprotectin in the study by Willers et al. Therefore, in no case was there a full consistency of microbiota changes between particular time points and changes in the levels of zonulin and/or calprotectin. This said the validation analysis suggested that the genus *Staphylococcus* and the family Staphylococcaceae (positively correlated with the concentration of calprotectin), as well as the family Ruminococcaceae and the order Clostridiales (negatively correlated with the concentration of calprotectin), are potential candidates as markers of the development of the immune system in children under 2 years of age.

Finally, we analyzed the metabolic pathways linked with bacterial abundance (Table 4). We were able to show that the pathways involved in the production of SCFAs and the metabolism of carbohydrates were positively associated with fecal zonulin and negatively with calprotectin. The latter association suggests the role of SCFAs in the immune response. Indeed, SCFAs regulate the body's immune response through free fatty acid receptors (FFARs) that are expressed throughout the body, including along the gastrointestinal tract [61]. SCFAs inhibit the activity of histone deacetylase (HADC), which activates gene expression in a pro-inflammatory response. Butyric acid plays a special role here via the binding of two butyrate molecules in the hydrophobic part of the enzyme, which is an example of the intracellular action of SCFAs. Moreover, the secretory activity of macrophages for the production of pro-inflammatory cytokines Il-6 and IL-12 is reduced along with the increase of butyrate content. Some studies suggest that SCFAs initiate the transformation of naive T_CD4+_ cells into regulatory cells, while others indicate SCFAs stimulate Treg cells already present in the colon [62–66]. Atarashi et al. [67, 68] demonstrated that colonization of germ free rodents with appropriate strains of Clostridium influenced the multiplication and differentiation of Treg cells and stimulated anti-inflammatory cytokines synthesis, predominantly interleukin 10 (IL-10). Notably, butyric acid of all SCFAs is metabolized to the greatest extent by the colon epithelial cells. Thus, it is the main source of energy for the colonocytes as it is responsible for maintaining the integrity of the intestinal barrier. This is evidenced by the effects of butyric acid supplementation of Caco 2 cells. Butyrate increased the concentration of ZO-1 and activated AMPK (adenosine monophospate activated protein kinase), which resulted in TEER (transepithelial electrical resistance) increase. It is also known that butyric acid regulates cell proliferation and apoptosis, protecting against colorectal cancer [69, 70]. However, the positive associations and correlations between pathways involved in SCFAs production and the zonulin elevation are difficult to explain, suggesting that other mechanisms are also involved in regulating small intestinal barrier permeability in children of up to 2 years old. It should be emphasized that the variations in the concentrations of zonulin and calprotectin overlapped with the introduction of new foods to the child’s diet around the sixth month of life (addition of soup, grated apple), with the consequent alteration in the gut microbiota composition and the related metabolic functions. For example, metabolic pathways involved in the metabolism of carbohydrates, vitamin B6 and nucleotides positively correlated with the levels of zonulin, which could suggest that an “open” small intestinal paracellular permeability could play a role in the transportation of metabolic reaction products during intensive growth and development. However, the paracellular transport of nutrients is known to play a rather minor role. The observed results support the concept that paracellular permeability is only the component of intestinal barrier [22] and that SCFAs affect mostly transcellular route via metabolism of enterocytes. Of note, the analysis of the validation data did not confirm our observations concerning the metabolic pathways. Therefore, shotgun sequencing and metabolomic analyses should be performed to shed more light on these processes. All associations between metabolic pathways and markers were shown in Table 4.

**Table 4:** Levels of associations between MetaCyc pathways and markers

Metabolic pathway	Change^a^		RMC	LME	Pathway
	ZON	CALP			
P-163 PWY		Same			L-lysine fermentation to acetate and butanoate
CENTFERM-PWY	Same			Calp (common slope model, negative β coeff.)	Pyruvate fermentation to butanoate
FUCCAT-PWY	Same		Zon ( +)		Fucose degradation
GLCMANNANAUT-PWY	Same				Superpathway of N-acetylglucosamine, N-acetylmannosamine and N-acetylneuraminate degradation
PWY-4984	Same				Urea cycle
PWY-6588	Same		Zon (+), Calp (−)		Pyruvate fermentation to acetone
PWY-6590	Same			Calp (common slope model, negative β coeff.)	Superpathway of *Clostridium acetobutylicum* acidogenic fermentation
PWY-6608	Same				Guanosine nucleotides degradation III
PWY-7003	Same		Zon (+)		Glycerol degradation to butanol
PWY-7013	Same				(S)-propane-1,2-diol degradation
PWY-7184	Opposite				Pyrimidine deoxyribonucleotides de novo biosynthesis I
PWY-7237	Same		Zon (+)		myo-, chiro- and scyllo-inositol degradation
FUC-RHAMCAT-PWY			Zon (+)		Superpathway of fucose and rhamnose degradation
PWY-5676			Zon (+)		Acetyl-CoA fermentation to butanoate II
PWY-5677			Zon (+), Calp (−)		Succinate fermentation to butanoate
PWY-6353			Zon (+)		Purine nucleotides degradation II (aerobic)
PWY-6478			Zon (+)		GDP-D-glycero-alpha-D-manno-heptose biosynthesis
PENTOSE-P-PWY				Calp (common slope model, positive β coeff.)	Pentose phosphate pathway
PWY-6545				Zon (different slopes model) negative β coeff.: P3-P4; positive β coeff.: P4–P6, P5–P6	Pyrimidine deoxyribonucleotides de novo biosynthesis III
PWY0-845				Zon (different slopes model) positive β coeff.: P3–P4	Superpathway of pyridoxal 5′-phosphate biosynthesis and salvage
PWY-6901				Zon (different slopes model) positive β coeff.: P3–P4, P5–P6	Superpathway of glucose and xylose degradation
SALVADEHYPOX-PWY			Zon (+)		Adenosine nucleotides degradation II

^a^co-occurrence between change in the pathway abundance and change in zonulin (P2-P4/P5/P6, P3-P4/P5/P6) or calprotectin (P2-P5/P6, P3-P5/P6, P4-P6) levels: same—a change in the pathway abundance and change in marker level between time points occur in the same direction, opposite—a change in the pathway abundance and change in marker level between time points occur in the opposite direction. RMC (repeated measures correlation)—correlation between the pathway abundance and concentration of zonulin/calprotectin for paired measures assessed on six occasions (from P1 to P6, i.e., the entire observation period); LME (linear mixed-effects analysis)—an association between the pathway abundance change and marker change accounting for multiple time point pairs from the same subject, including time-point pair-specific relationships with either absence (the common slope model) or presence of the interaction (the different slopes model) between the abundance change and the time point pair); (+), positive RMC coefficient; (−), negative RMC coefficient

Our study is not without limitations. Our cohort was relatively small, though enough to observe changes in zonulin and calprotectin levels as well as in gut microbiota composition during the first 2 years of life with appropriate power. The relatively small size of our dataset (see power calculation section) does mean it is likely we may underestimate the relationship between permeability markers and the microbiota resulting in false negative findings, though we anticipate no increased risk of false positive such. However, the dataset size was at least sufficient to observe changes in zonulin and calprotectin levels as well as in gut microbiota compositions, even at this reduced power. Moreover, our results were validated using the HMS cohort as well as via the comparison between the microbiota of meconium and stool samples collected at other time points. We therefore consider our findings reliable although likely still incomplete. Moreover, our samples were somewhat heterogeneous (with respect to the delivery mode, the use of antibiotics, nutrition, and other environmental factors). Of note, all of these factors and age development can affect the gut microbiota; however, in term infants, the mode of delivery seems to impact fecal calprotectin levels only in the first week of life [25]. Moreover, in our study, we did not use direct methods to measure the gut permeability; we used biomarkers, for which data in healthy children < 2 years old are limited. In fact, there are studies reported on the fecal levels of zonulin in adults but not in children. It is known that in children, the levels of zonulin and calprotectin are incomparably higher than those in adults, changing over time, and could be influenced by e.g., breastfeeding, the dietary patterns, the mode of delivery, and the consumption of gliadin. In the current study, however, due to the small sample size, we did not analyze these biomarkers separately in the context of different delivery modes. Also, we did not collect detailed data on antibiotic exposure. Further, we did not measure these factors in blood (ethical reason—healthy children); only in stool. Moreover, since no metabolomic, lipidomic, immunological, or shotgun metagenomic analyses were performed, the results we obtained must be treated with caution. Mechanistic studies are still needed to investigate the relationship between microbiota and gut permeability and the immune system.

## Conclusions

Overall, based on the results, we can conclude that the gut microbiota composition, the small intestinal paracellular permeability, as well as immune system-related markers change dynamically during the first 2 years of life. Although the gut microbiota composition and the related metabolic functions were correlated with zonulin and calprotectin levels, neither clear causation nor obvious health consequences can be proven in this study. However, our data may suggest that the *Ruminococcus* (*torques* group) might be more involved in controlling paracellular permeability during the first 2 years of life than previously considered. Additionally, our data indicate that the genus *Staphylococcus* and the family Staphylococcaceae (positively correlated with the fecal calprotectin concentration) and the family Ruminococcaceae, as well as the order Clostridiales (negatively correlated with the fecal calprotectin concentration), may be potential biomarkers of the development of the immune system development or inflammatory reactions in children younger than 2 years old. It must be emphasized that further longitudinal studies are required to investigate the mechanism behind gut permeability in children and its influence on health. The establishment of gut permeability markers optimal for children as well as germ-free animal models, together with immunological, metabolomic and lipidomic studies (including the analysis of SCFAs), are essential. This study's translational significance may follow from our observation may be that due to the of persistence of increased intercellular permeability, at least until the age of two. If these observations can be robustly replicated, it may indicate health benefits of restricting exposure of very young children the child's exposure to potential antigens, both food and environmental, should be limited, balanced against the relative benefit of such exposure under the “hygiene hypothesis” of autoimmune aetiology, at least in particular subsets of vulnerable children. Bacterial and metabolic pathways associated with permeability markers may provide a basis for the search for pro- and postbiotics, and possibly serving as markers of intestinal permeability and gut immunological status in young children that may help stratify recommendations.

## Supplementary Information

**Additional file 1: Figure S1.** Sample selection and availability (PMU cohort). From 100 healthy, full-term newborns during the period from March 2015 to April 2016, 18 mother + child pairs were initially selected with the highest number of samples available. Since only in two cases the delivery was natural and neither the mother nor the child was treated with antibiotics, it was decided to supplement this cohort with six pairs of mother + child who were not given antibiotics and the delivery was natural. Out of the 24 newborns that were selected in this way, three newborns (F19, F22, F24) were excluded due to inadequate number of samples. Twenty-one newborns (101 samples in total, green tiles) were included, in whom at least four longitudinal stool samples were available. **Figure S2**. Study flow chart, including zonulin and calprotectin (PMU cohort). The number of samples for downstream analyses might differ due to results out of determination limits (zonulin 800 ng/mL and calprotectin 2100 ug/mL), technical problems (small volume of collected stool specimen, inadequate amount of DNA, sequencing depth), participant attrition. **Figure S3.** Stool calprotectin level by time in the HMS cohort. Likelihood ratio test, df = 3, P = 2.56e−10, adjusted for mode of delivery Notched boxplot with variable widths proportional to the square-roots of the number of observations in the groups; FDR adjusted p-values < 0.05 are shown, P2—10th day, P3—1st month, P4—6th month, P5**—**12th month. **Figure S4.** Alpha and beta diversity over time in the HMS cohort. **A**—Shannon alpha diversity by time, LRT, df = 4, P < 2.2e−16, adjusted for mode of delivery, notched boxplot with variable widths proportional to the square-roots of the number of observations in the groups, **B**—Principal coordinate analysis plot with Bray–Curtis dissimilarity calculated from genus abundances, ellipses were drawn assuming a multivariate t-distribution, **C**—PCo1 scores by time, LRT, df = 4, P = 2.97e−14, adjusted for mode of delivery; **D**—PCo2 scores by time, LRT, df = 4, P = 7.31e−11; FDR adjusted p values < 0.05 are shown P1—1st day (meconium/the first stool), P2—10th day, P3—1st month, P4—6th month, P5**—**12th month. **Figure S5**. Gut microbiota composition change over time (PMU cohort). A linear mixed effects analysis followed by pairwise comparison of time points (adjusted for mode of delivery and breastfeeding time). The overall p-value—a likelihood ratio test (LRT) of nested models (FDR adjusted across genera); PXPY—contrast p values between the two time points (PX and PY), FDR adjusted for all possible contrasts, t.ratio—t statistics for the contrasts estimates (a positive value, colored blue, indicates a decrease abundance, a negative value, colored red, indicates increase in abundance). Taxa abundances (unrarefied) were transformed by generating 128 Monte Carlo instances of the Dirichlet distribution for each gut sample, followed by center-logtransform of each instance. A linear mixed effects analysis was performed for each instance separately and the results were averaged over 128 instances. P2—7th day, P3—1st month, P4—6th month, P5—12th month, P6—24th month. **Figure S6.** Gut microbiota composition change over time (HMS cohort). A linear mixed effects analysis followed by pairwise comparison of time points (adjusted for mode of delivery) for five taxonomic ranks (present in at least 10% samples), only taxons significantly associated with time are shown. The overall p-value—a likelihood ratio test (LRT) of nested models (FDR adjusted across taxons); PXPY—contrast p-values between the two time points (PX and PY), FDR adjusted for all possible contrasts, t.ratio—t statistics for the contrasts estimates (a positive value, colored blue, indicates a decrease in abundance, a negative value, colored red, indicates an increase in abundance). Taxa abundances (unrarefied) were transformed by generating 128 Monte Carlo instances of the Dirichlet distribution for each gut sample, followed by center-log transform of each instance. A linear mixed effects analysis was performed for each instance separately and the results were averaged over 128 instances. P2**—**10th day**,** P3**—**1st month**,** P4**—**6th month**,** P5**—**12th month. **Figure S7.** Predicted MetaCyc pathways that change significantly over time in both cohorts (PMU and HMS). Linear mixed effects analysis followed by pairwise comparison of time points (adjusted for mode of delivery and breastfeeding time (PMU) and mode of delivery only in the HMS cohort). The overall p-value—a likelihood ratio test (LRT) of nested models (FDR adjusted across pathways); PXPY—contrast p values between the two time points (X and Y), FDR adjusted for all possible contrasts, t.ratio—t statistics for the contrasts estimates. Pathway abundances (unrarefied) were transformed by generating 128 Monte Carlo instances of the Dirichlet distribution for each gut sample, followed by center-log transform of each instance. A linear mixed effects analysis was performed for each instance separately and the results were averaged over 128 instances. P2**—**7th day (PMU) or 10th day (HMS)**,** P3**—**1st month**,** P4**—**6th month**,** P5**—**12th month, P6—24th month (PMU only). **Figure S8**. Taxon change versus zonulin change (PMU cohort). Linear mixed effects models were used to test for an association between the taxon abundance change and zonulin change accounting for ten time point pairs (P2-P3, P3-P4, etc.) from the same subject**,** adjusted for mode of delivery and breastfeeding time. Two models were considered: without interaction (w/o int.) and with interaction between time point pair and taxon change (w/ int.). If the interaction term was significant—Q (w/ int.) < 0.05, individual p values (FDR adjusted) were interpreted. If the common slope model was chosen—Q (w/o int.) < 0.05 and Q (w /int) > 0.05, the same common slope coefficient β (coef.) across all time points and one p-value (P2–P3) were shown. Coefficients and Q values were averaged over 128 Monte Carlo instances of the Dirichlet distribution, followed by center-log transform of each instance. **Figure S9.** Taxon change versus calprotectin change (PMU cohort). Linear mixed effects models were used to test for an association between the taxon abundance change and calprotectin change accounting for ten time point pairs (P2-P3, P3-P4, etc.) from the same subject**,** adjusted for mode of delivery and breastfeeding time. Two models were considered: without interaction (w/o int.) and with interaction between time point pair and taxon change (w/ int.). If the interaction term was significant—Q (w/ int.) < 0.05, individual p values (FDR adjusted) were interpreted. If the common slope model was chosen—Q (w/o int.) < 0.05 and Q (w /int) > 0.05, the same common slope coefficient β (coef.) across all time points and one p-value (P2-P3) were shown. Coefficients and Q values were averaged over 128 Monte Carlo instances of the Dirichlet distribution, followed by center-log transform of each instance. **Figure S10.** Taxon change versus calprotectin change (HMS cohort). Linear mixed effects models were used to test for an association between the taxon abundance change and calprotectin change accounting for six time point pairs (P2–P3, P3–P4, etc.) from the same subject**,** adjusted for mode of delivery. Two models were considered: without interaction (w/o int.) and with interaction between time point pair and taxon change (w/ int.). If the interaction term was significant—Q (w/ int.) < 0.05, individual p values (FDR adjusted) were interpreted. If the common slope model was chosen—Q (w/o int.) < 0.05 and Q (w /int) > 0.05, the same common slope coefficient β (coef.) across all time points and one p-value (P2-P3) were shown. Coefficients and Q values were averaged over 128 Monte Carlo instances of the Dirichlet distribution, followed by center-log transform of each instance. **Figure S11.** Pathway change vs calprotectin change (HMS cohort). Linear mixed effects models were used to test for an association between the pathway abundance change and calprotectin change accounting for six time point pairs (P2–P3, P3–P4, etc.) from the same subject**,** adjusted for mode of delivery. Two models were considered: without interaction (w/o int.) and with interaction between time point pair and taxon change (w/ int.). If the interaction term was significant—Q (w/int.) < 0.05, individual p values (FDR adjusted) were interpreted. If the common slope model was chosen—Q (w/o int.) < 0.05 and Q (w /int) > 0.05, the same common slope coefficient β (coef.) across all time points and one p-value (P2-P3) were shown. Coefficients and Q values were averaged over 128 Monte Carlo instances of the Dirichlet distribution, followed by center-log transform of each instance. **Figure S12.** Relationship between (changes in) gut abundance of inferred MetaCyc pathways and (changes in) calprotectin levels (HMS cohort). **A—**common slope model illustrating relationships between CENTFERM-PWY change (centered log-ratio transformed) and calprotectin change (rank transformed), **B—**common slope model illustrating relationships between PWY-6590 change (centered log-ratio transformed) and calprotectin change (rank transformed); Likelihood ratio test (LRT) p- and Q-values were computed based on 128 Monte Carlo instances of the Dirichlet distribution, followed by center-log transform of each instance, the plots were based on the first Monte Carlo instance. **Table S1.** Repeated measures correlation of zonulin and calprotectin with microorganisms and predicted MetaCyc pathways (PMU cohort). **Table S2.** Repeated measures correlation of calprotectin with bacteria and MetaCyc pathway abundance in the PMU and HMS cohorts. **Table S3.** Pathway change vs Zonulin change (PMU cohort). **Table S4.** Pathway change vs Calprotectin change (PMU cohort).

## Data Availability

The datasets used and/or analysed during the current study are available from the corresponding author on reasonable request. Data for validation were downloaded from www.ncbi.nlm.nih.gov/sra (BioProject accession number PRJNA514340).

## Abbreviations

DV: Dependent Variable
FDR: False Discovery Rate
HMS: Hannover Medical School
IBD: Inflammatory Bowel Diseases
IBS-D: Irritable Bowel Syndrome (Diarrhea)
LME: Linear Mixed-Effects Analysis
LRT: Likelihood Ratio Test
NGS: Next Generation Sequencing
OUT: Operational Taxonomic Unit
P: Time Point
PICRUSt: Phylogenetic Investigation Of Communities By Reconstruction Of Unobserved States
PMU: Pomeranian Medical University
RMC: Repeated Measures Correlation
RTK: Rarefaction Toolkit
SAS: Stool Sample Application System
SCFAs: Short Chain Fatty Acids
TJ: Tight Junctions
ZON: Zonulin
ZOT: Zonula Ocludens Toxin
