# Supplementary material for: The gut microbiota is associated with the small intestinal paracellular permeability and the development of the immune system in healthy children during the first two years of life
Source: J Transl Med. 2021 Apr 28;19:177. doi: 10.1186/s12967-021-02839-w (PMC8082808; doi:10.1186/s12967-021-02839-w)
Supplement: Supplementary file 1 — Additional file 1: Figure S1. Sample selection and availability (PMU cohort). From 100 healthy, full-term newborns during the period from March 2015 to April 2016, 18 mother + child pairs were initially selected with the highest number of samples available. Since only in two cases the delivery was natural and neither the mother nor the child was treated with antibiotics, it was decided to supplement this cohort with six pairs of mother + child who were not given antibiotics and the delivery was natural. Out of the 24 newborns that were selected in this way, three newborns (F19, F22, F24) were excluded due to inadequate number of samples. Twenty-one newborns (101 samples in total, green tiles) were included, in whom at least four longitudinal stool samples were available. Figure S2. Study flow chart, including zonulin and calprotectin (PMU cohort). The number of samples for downstream analyses might differ due to results out of determination limits (zonulin 800 ng/mL and calprotectin 2100 ug/mL), technical problems (small volume of collected stool specimen, inadequate amount of DNA, sequencing depth), participant attrition. Figure S3. Stool calprotectin level by time in the HMS cohort. Likelihood ratio test, df = 3, P = 2.56e−10, adjusted for mode of delivery Notched boxplot with variable widths proportional to the square-roots of the number of observations in the groups; FDR adjusted p-values < 0.05 are shown, P2—10th day, P3—1st month, P4—6th month, P5—12th month. Figure S4. Alpha and beta diversity over time in the HMS cohort. A—Shannon alpha diversity by time, LRT, df = 4, P < 2.2e−16, adjusted for mode of delivery, notched boxplot with variable widths proportional to the square-roots of the number of observations in the groups, B—Principal coordinate analysis plot with Bray–Curtis dissimilarity calculated from genus abundances, ellipses were drawn assuming a multivariate t-distribution, C—PCo1 scores by time, LRT, df = 4, P = 2.97e−14, adjusted for mode of deliv [file 12967_2021_2839_MOESM1_ESM.docx]

## The gut microbiota is associated with the small intestinal paracellular permeability and the development of the immune system in healthy children during the first two years of life

## Mariusz Kaczmarczyk, Ulrike Löber, Karolina Adamek, Dagmara Węgrzyn, Karolina Skonieczna-Żydecka, Damian Malinowski, Igor Łoniewski, Lajos Markó, Thomas Ulas, Sofia K. Forslund, Beata Łoniewska

## Supplementary materials


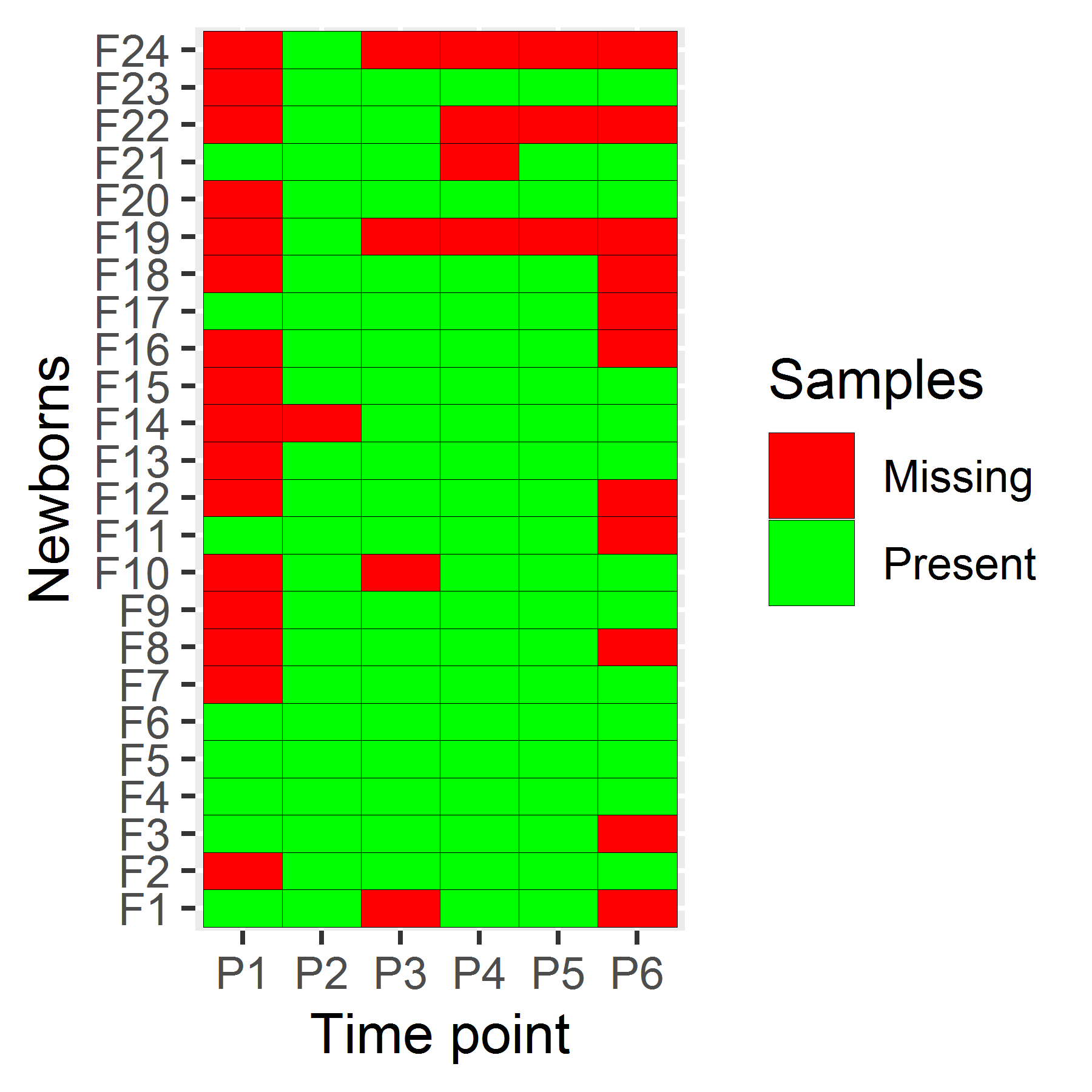


**Figure S1. Sample selection and availability (PMU cohort)**

From 100 healthy, full-term newborns during the period from March 2015 to April 2016, 18 mother + child pairs were initially selected with the highest number of samples available. Since only in two cases the delivery was natural and neither the mother nor the child was treated with antibiotics, it was decided to supplement this cohort with six pairs of mother + child who were not given antibiotics and the delivery was natural. Out of the 24 newborns that were selected in this way, three newborns (F19, F22, F24) were excluded due to inadequate number of samples. Twenty-one newborns (101 samples in total, green tiles) were included, in whom at least four longitudinal stool samples were available.

**
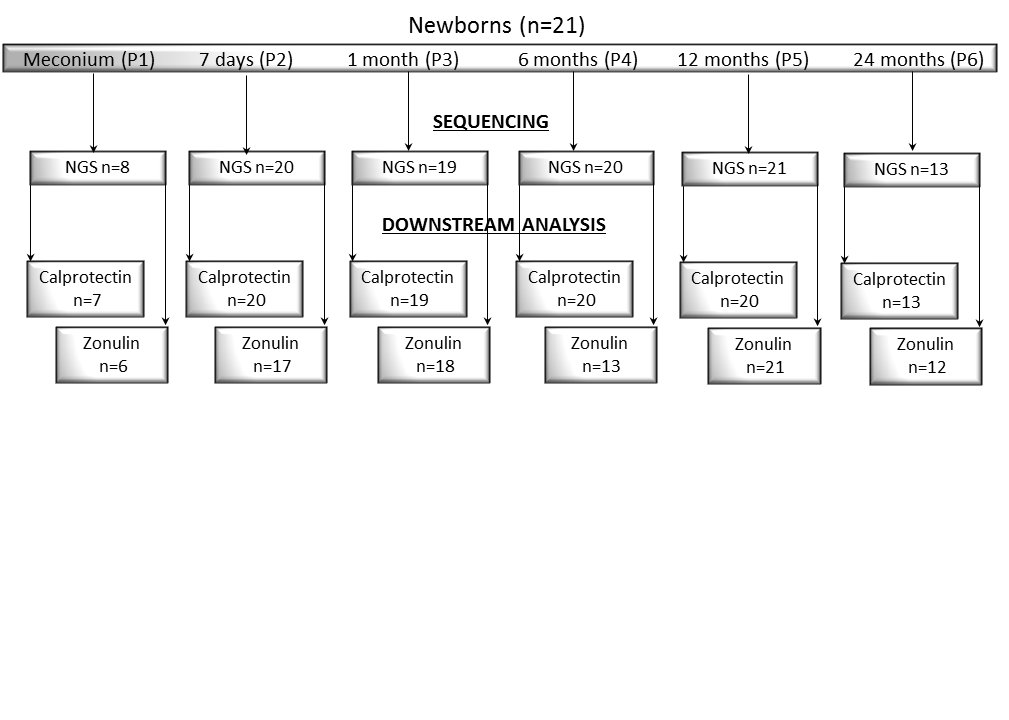
**

### Figure S2. Study flow chart, including zonulin and calprotectin (PMU cohort)

The number of samples for downstream analyses might differ due to results out of determination limits (zonulin 800 ng/mL and calprotectin 2100 ug/mL), technical problems (small volume of collected stool specimen, inadequate amount of DNA, sequencing depth), participant attrition.


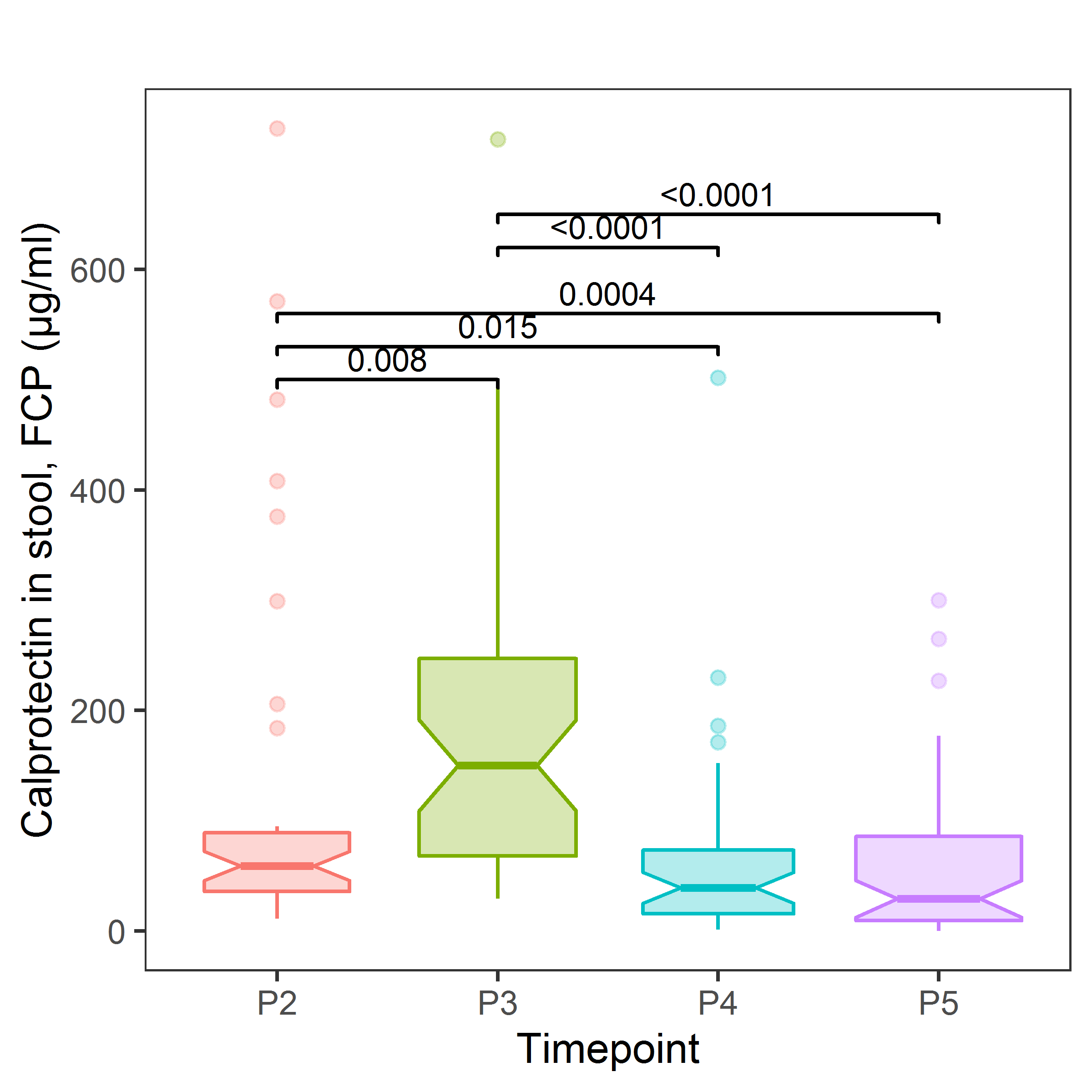


### Figure S3. Stool calprotectin level by time in the HMS cohort

Likelihood ratio test, df=3, P=2.56e-10, adjusted for mode of delivery Notched boxplot with variable widths proportional to the square-roots of the number of observations in the groups; FDR adjusted p-values < 0.05 are shown, P2 - 10^th^ day, P3 - 1^st^ month, P4 - 6^th^ month, P5 **-** 12^th^ month.

###

###
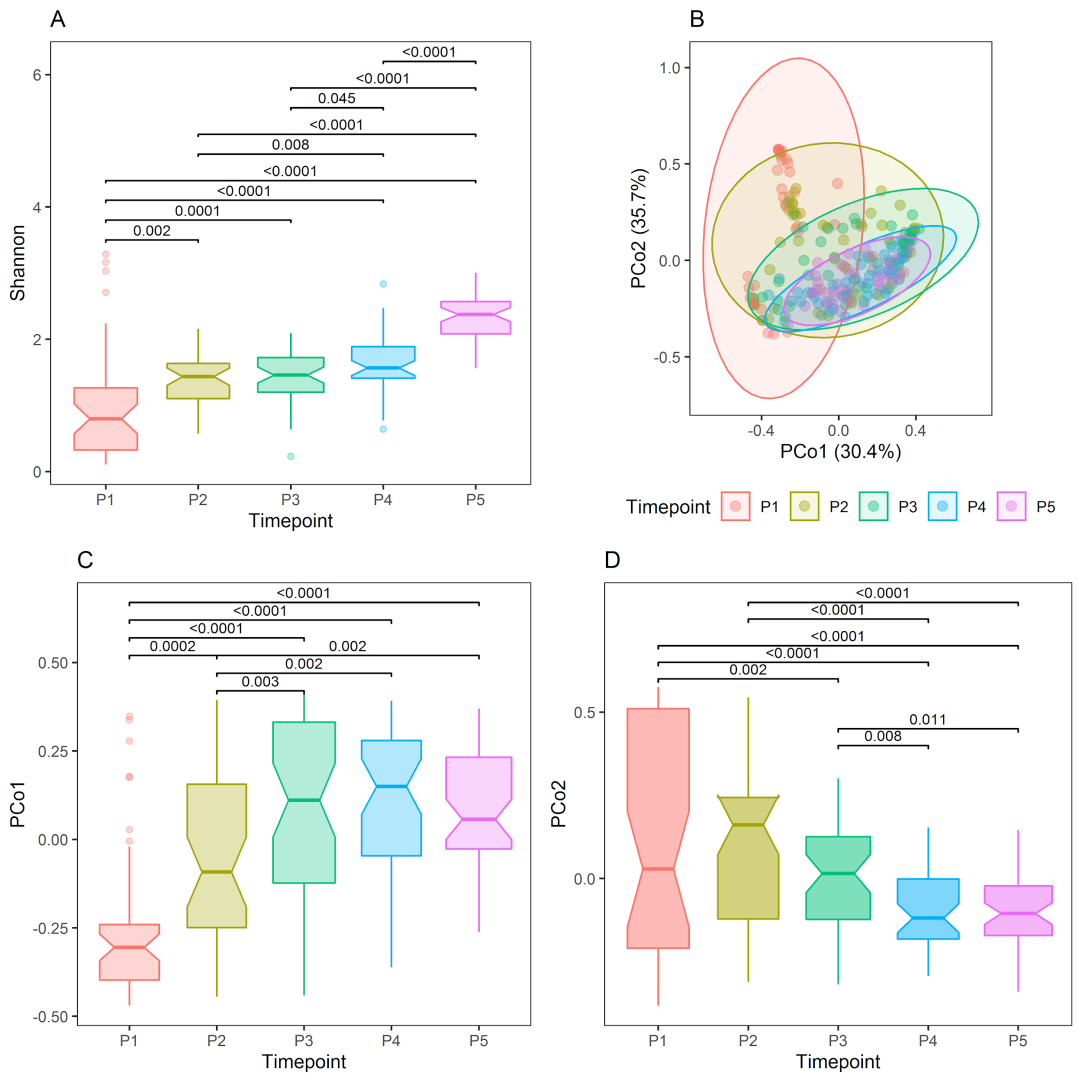


**Figure S4. Alpha and beta diversity over time in the HMS cohort**

**A** - Shannon alpha diversity by time, LRT, df=4, P< 2.2e-16, adjusted for mode of delivery, notched boxplot with variable widths proportional to the square-roots of the number of observations in the groups, **B** - Principal coordinate analysis plot with Bray-Curtis dissimilarity calculated from genus abundances, ellipses were drawn assuming a multivariate t-distribution, **C** - PCo1 scores by time, LRT, df = 4, P = 2.97e-14, adjusted for mode of delivery; **D** - PCo2 scores by time, LRT, df = 4, P = 7.31e-11; FDR adjusted p values < 0.05 are shown P1 - 1^st^ day (meconium/the first stool), P2 - 10^th^ day, P3 - 1^st^ month, P4 - 6^th^ month, P5 **-** 12^th^ month


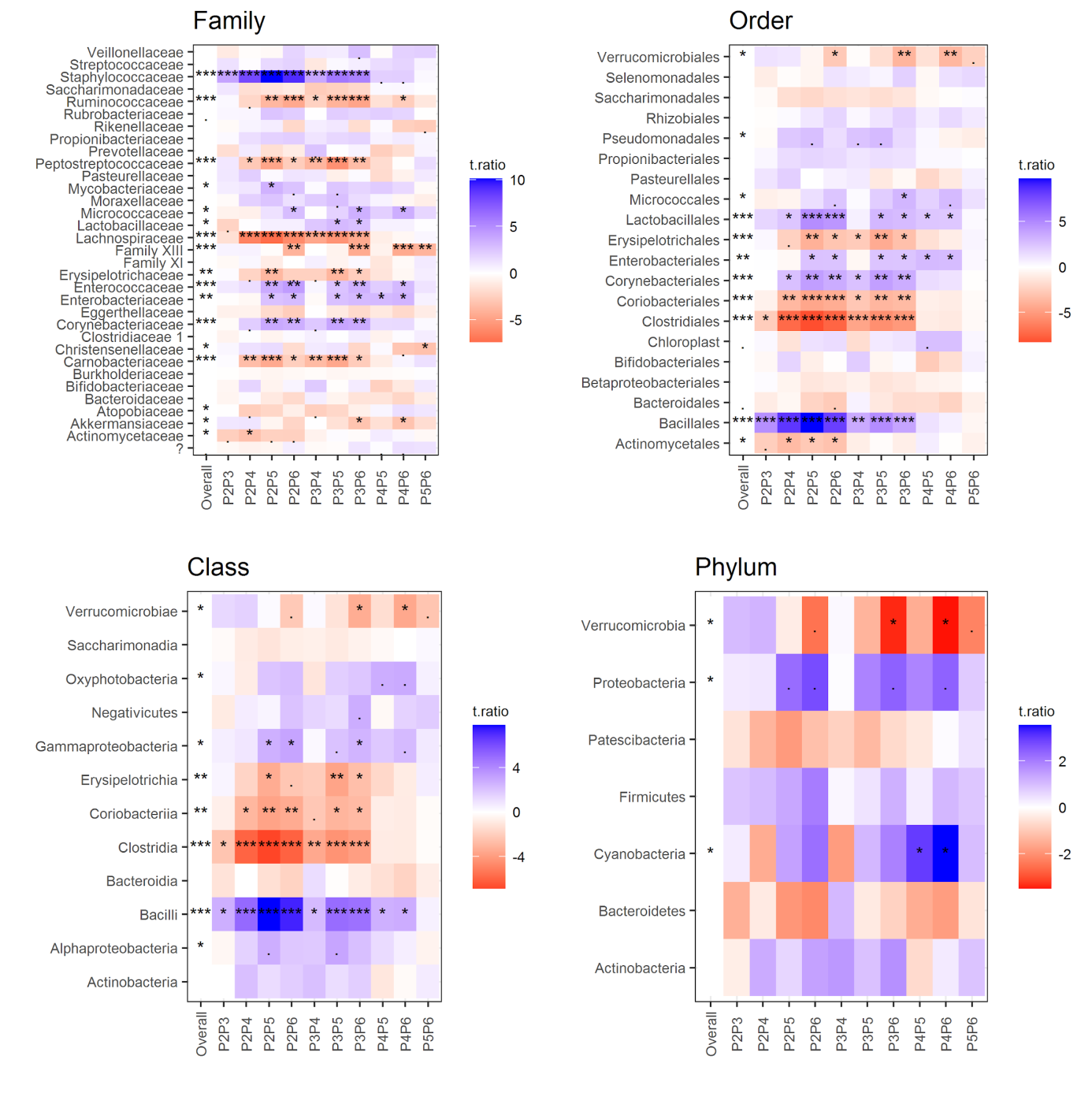


### Figure S5. Gut microbiota composition change over time (PMU cohort)

A linear mixed effects analysis followed by pairwise comparison of time points (adjusted for mode of delivery and breastfeeding time). The overall p-value - a likelihood ratio test (LRT) of nested models (FDR adjusted across genera); PXPY - contrast p values between the two time points (PX and PY), FDR adjusted for all possible contrasts, t.ratio - t statistics for the contrasts estimates (a positive value, colored blue, indicates a decrease abundance, a negative value, colored red, indicates increase in abundance). Taxa abundances (unrarefied) were transformed by generating 128 Monte Carlo instances of the Dirichlet distribution for each gut sample, followed by center-logtransform of each instance. A linear mixed effects analysis was performed for each instance separately and the results were averaged over 128 instances. P2 - 7^th^ day, P3 - 1^st^ month, P4 - 6^th^ month, P5 - 12^th^ month, P6 - 24^th^ month.


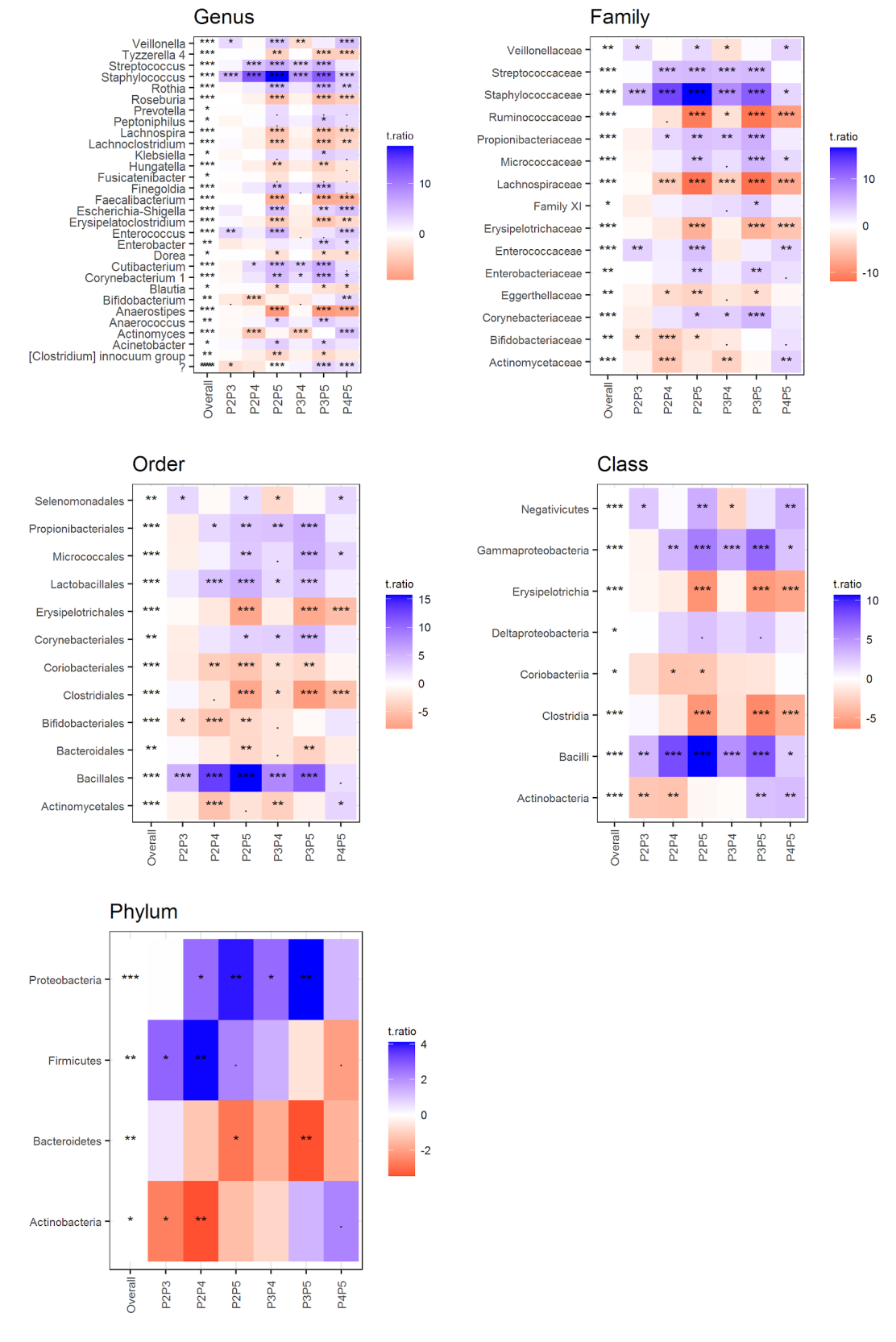


### Figure S6. Gut microbiota composition change over time (HMS cohort)

A linear mixed effects analysis followed by pairwise comparison of time points (adjusted for mode of delivery) for five taxonomic ranks (present in at least 10% samples), only taxons significantly associated with time are shown. The overall p-value - a likelihood ratio test (LRT) of nested models (FDR adjusted across taxons); PXPY - contrast p-values between the two time points (PX and PY), FDR adjusted for all possible contrasts, t.ratio - t statistics for the contrasts estimates (a positive value, colored blue, indicates a decrease in abundance, a negative value, colored red, indicates an increase in abundance). Taxa abundances (unrarefied) were transformed by generating 128 Monte Carlo instances of the Dirichlet distribution for each gut sample, followed by center-log transform of each instance. A linear mixed effects analysis was performed for each instance separately and the results were averaged over 128 instances. P2 **-** 10^th^ day**,** P3 **-** 1^st^ month**,** P4 **-** 6^th^ month**,** P5 **-** 12^th^ month.


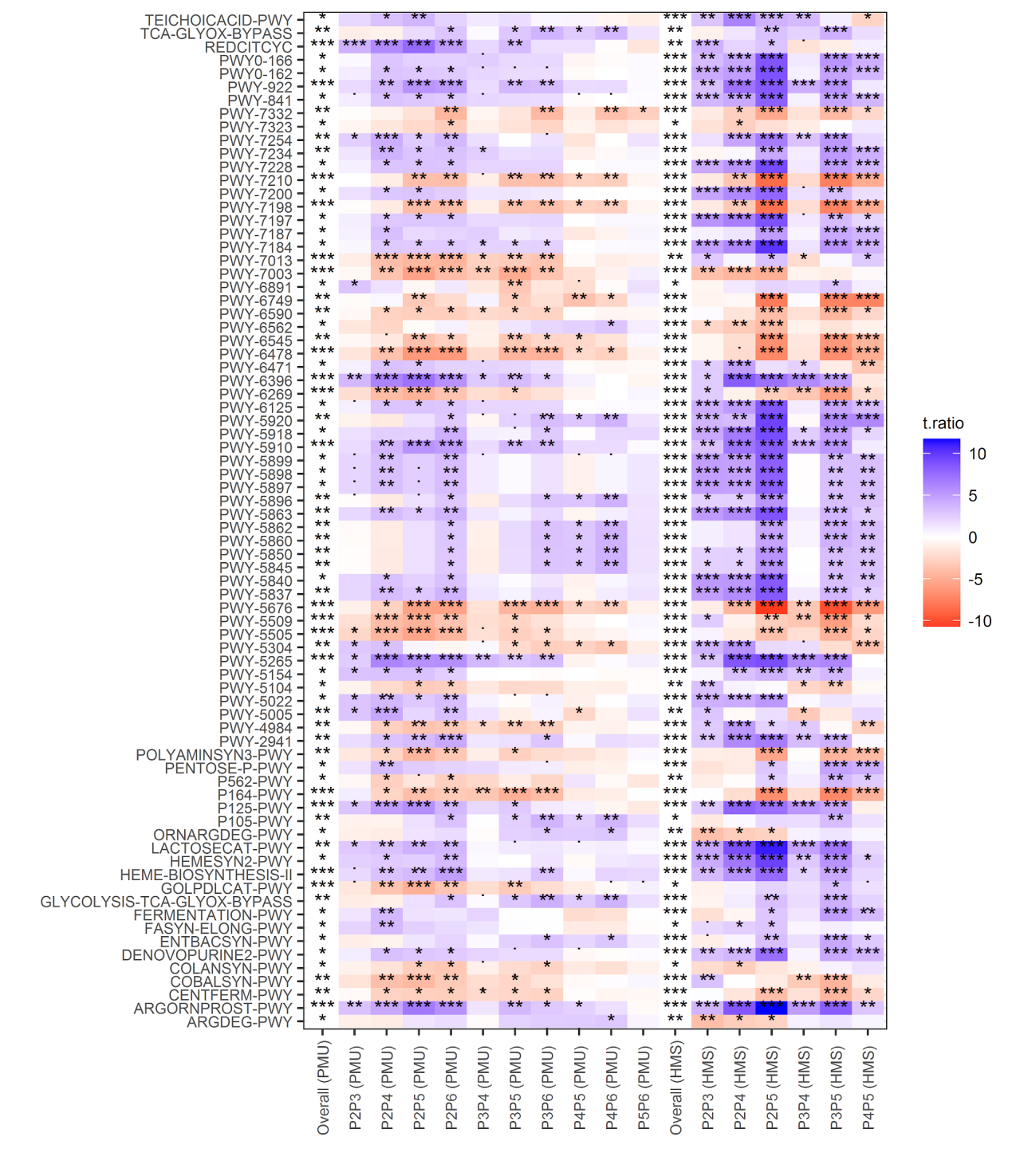


### Figure S7. Predicted MetaCyc pathways that change significantly over time in both cohorts (PMU and HMS)

Linear mixed effects analysis followed by pairwise comparison of time points (adjusted for mode of delivery and breastfeeding time (PMU) and mode of delivery only in the HMS cohort). The overall p-value - a likelihood ratio test (LRT) of nested models (FDR adjusted across pathways); PXPY - contrast p values between the two time points (X and Y), FDR adjusted for all possible contrasts, t.ratio - t statistics for the contrasts estimates. Pathway abundances (unrarefied) were transformed by generating 128 Monte Carlo instances of the Dirichlet distribution for each gut sample, followed by center-log transform of each instance. A linear mixed effects analysis was performed for each instance separately and the results were averaged over 128 instances. P2 **-** 7^th^ day (PMU) or 10^th^ day (HMS)**,** P3 **-** 1^st^ month**,** P4 **-** 6^th^ month**,** P5 **-** 12^th^ month, P6 - 24^th^ month (PMU only).


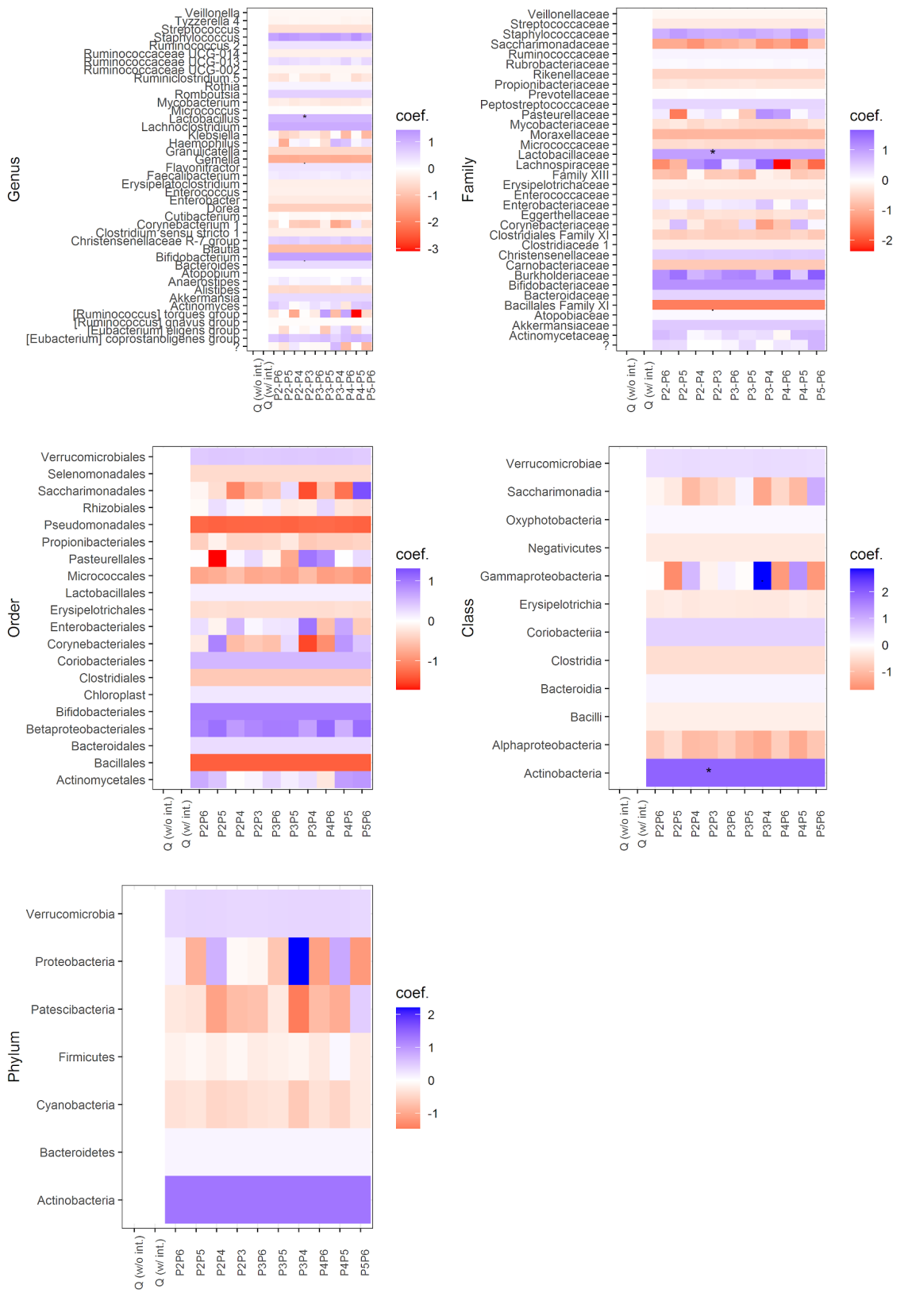


### Figure S8. Taxon change versus zonulin change (PMU cohort)

Linear mixed effects models were used to test for an association between the taxon abundance change and zonulin change accounting for ten time point pairs (P2-P3, P3-P4, etc.) from the same subject**,** adjusted for mode of delivery and breastfeeding time. Two models were considered: without interaction (w/o int.) and with interaction between time point pair and taxon change (w/ int.). If the interaction term was significant - Q (w/ int.) < 0.05, individual p values (FDR adjusted) were interpreted. If the common slope model was chosen - Q (w/o int.) < 0.05 and Q (w /int) > 0.05, the same common slope coefficient β (coef.) across all time points and one p-value (P2-P3) were shown. Coefficients and Q values were averaged over 128 Monte Carlo instances of the Dirichlet distribution, followed by center-log transform of each instance.

###
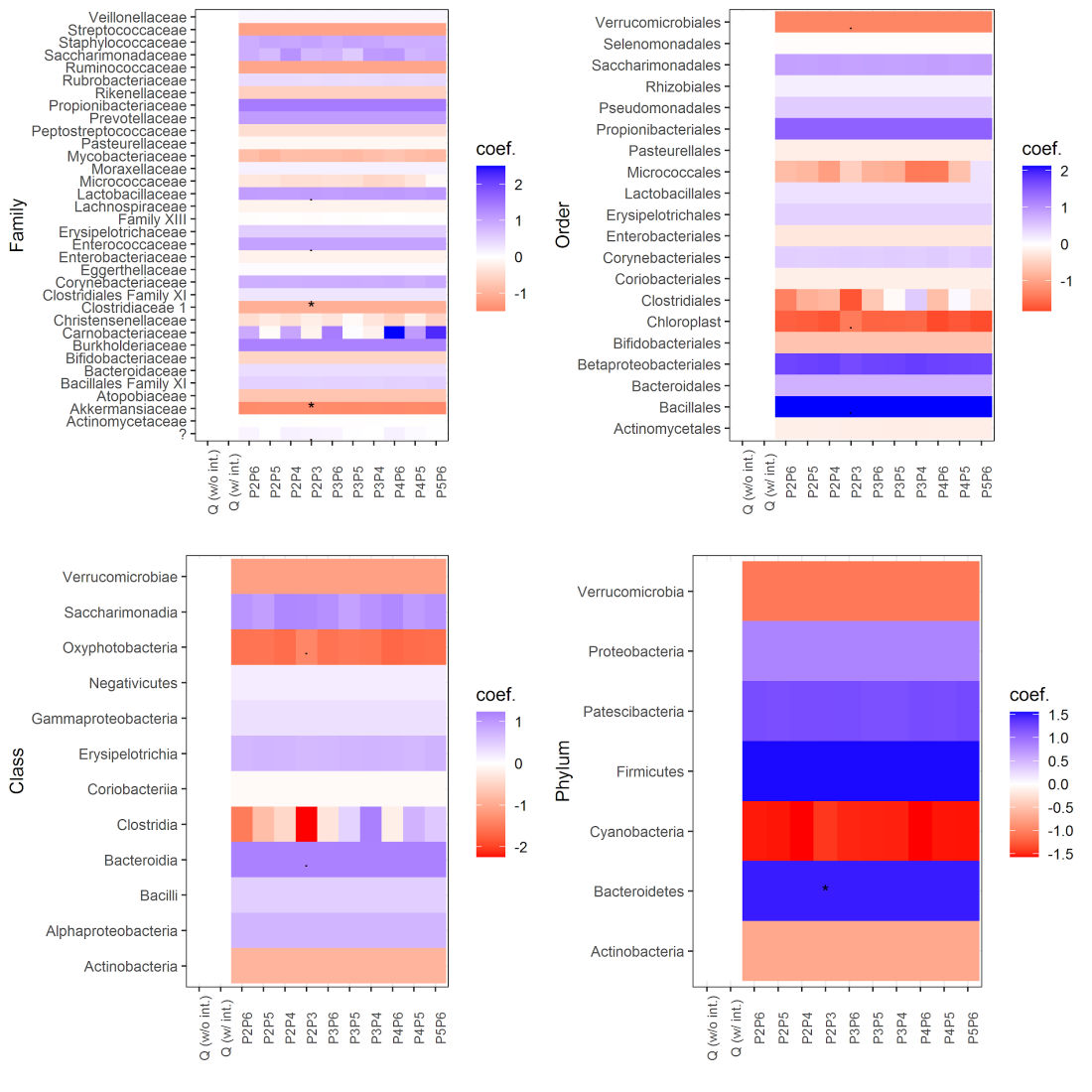


### Figure S9. Taxon change versus calprotectin change (PMU cohort)

Linear mixed effects models were used to test for an association between the taxon abundance change and calprotectin change accounting for ten time point pairs (P2-P3, P3-P4, etc.) from the same subject**,** adjusted for mode of delivery and breastfeeding time. Two models were considered: without interaction (w/o int.) and with interaction between time point pair and taxon change (w/ int.). If the interaction term was significant - Q (w/ int.) < 0.05, individual p values (FDR adjusted) were interpreted. If the common slope model was chosen - Q (w/o int.) < 0.05 and Q (w /int) > 0.05, the same common slope coefficient β (coef.) across all time points and one p-value (P2-P3) were shown. Coefficients and Q values were averaged over 128 Monte Carlo instances of the Dirichlet distribution, followed by center-log transform of each instance.


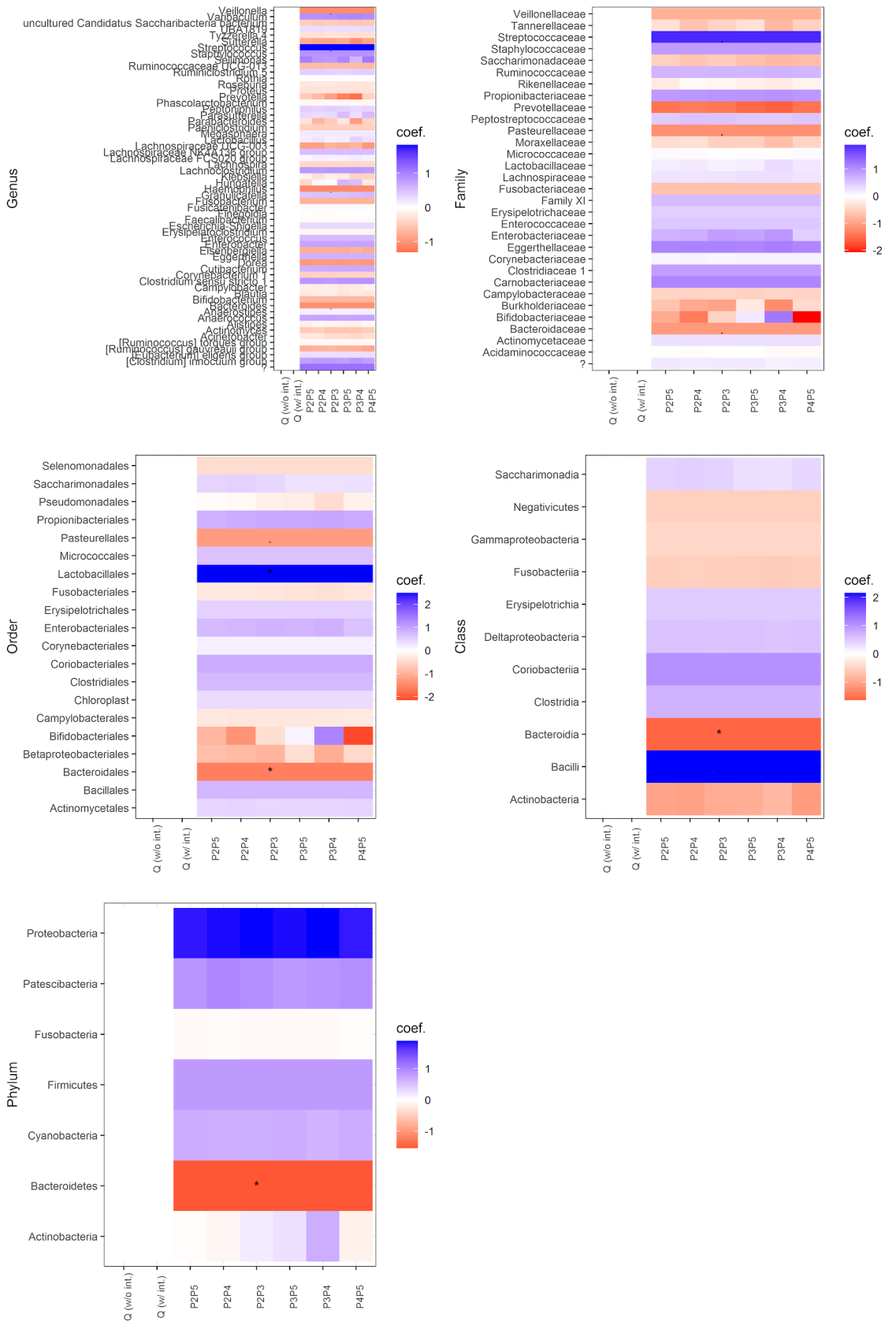


### Figure S10. Taxon change versus calprotectin change (HMS cohort)

Linear mixed effects models were used to test for an association between the taxon abundance change and calprotectin change accounting for six time point pairs (P2-P3, P3-P4, etc.) from the same subject**,** adjusted for mode of delivery. Two models were considered: without interaction (w/o int.) and with interaction between time point pair and taxon change (w/ int.). If the interaction term was significant - Q (w/ int.) < 0.05, individual p values (FDR adjusted) were interpreted. If the common slope model was chosen - Q (w/o int.) < 0.05 and Q (w /int) > 0.05, the same common slope coefficient β (coef.) across all time points and one p-value (P2-P3) were shown. Coefficients and Q values were averaged over 128 Monte Carlo instances of the Dirichlet distribution, followed by center-log transform of each instance.


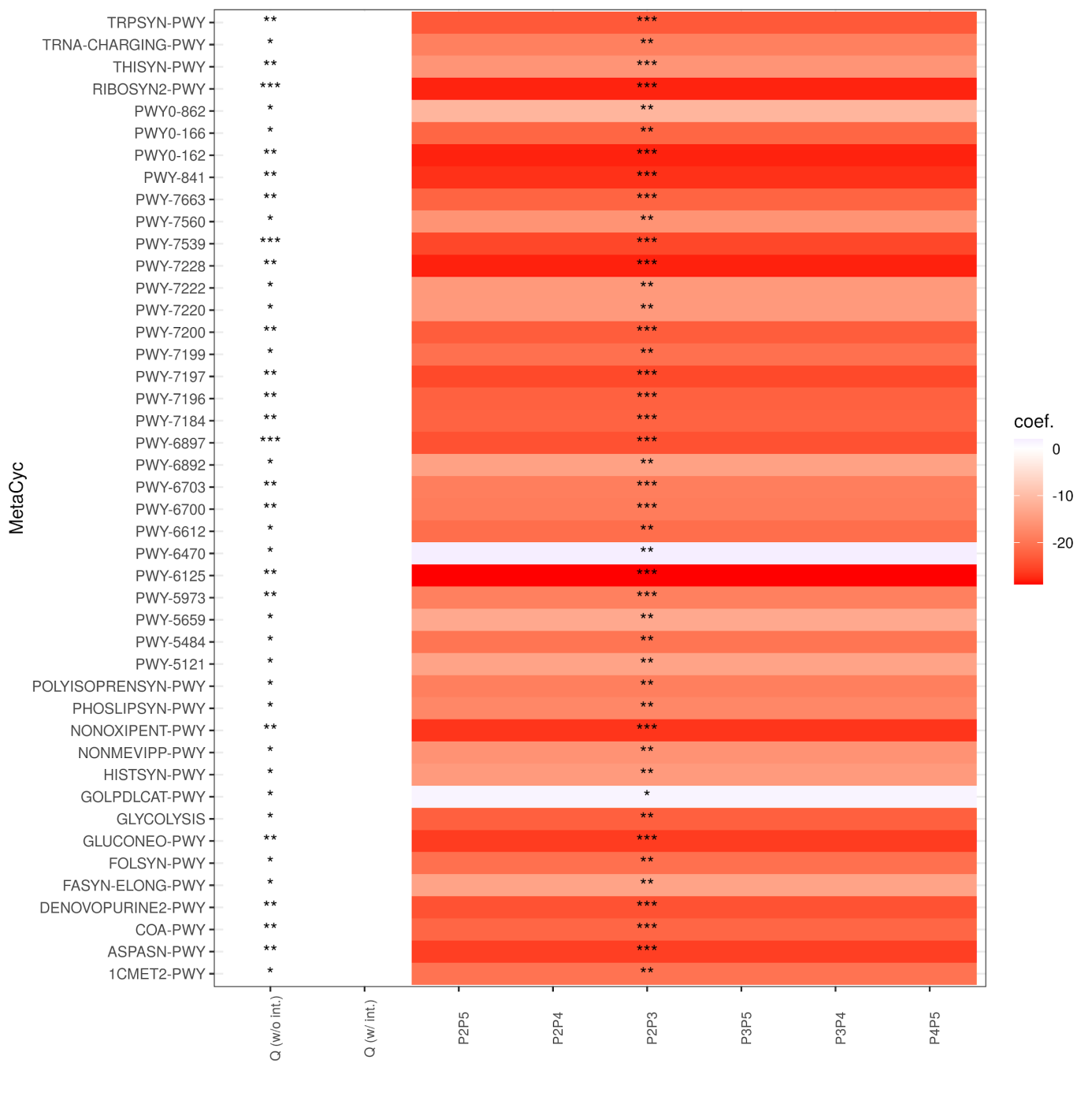


### Figure S11. Pathway change vs calprotectin change (HMS cohort)

Linear mixed effects models were used to test for an association between the pathway abundance change and calprotectin change accounting for six time point pairs (P2-P3, P3-P4, etc.) from the same subject**,** adjusted for mode of delivery. Two models were considered: without interaction (w/o int.) and with interaction between time point pair and taxon change (w/ int.). If the interaction term was significant - Q (w/ int.) < 0.05, individual p values (FDR adjusted) were interpreted. If the common slope model was chosen - Q (w/o int.) < 0.05 and Q (w /int) > 0.05, the same common slope coefficient β (coef.) across all time points and one p-value (P2-P3) were shown. Coefficients and Q values were averaged over 128 Monte Carlo instances of the Dirichlet distribution, followed by center-log transform of each instance.

**
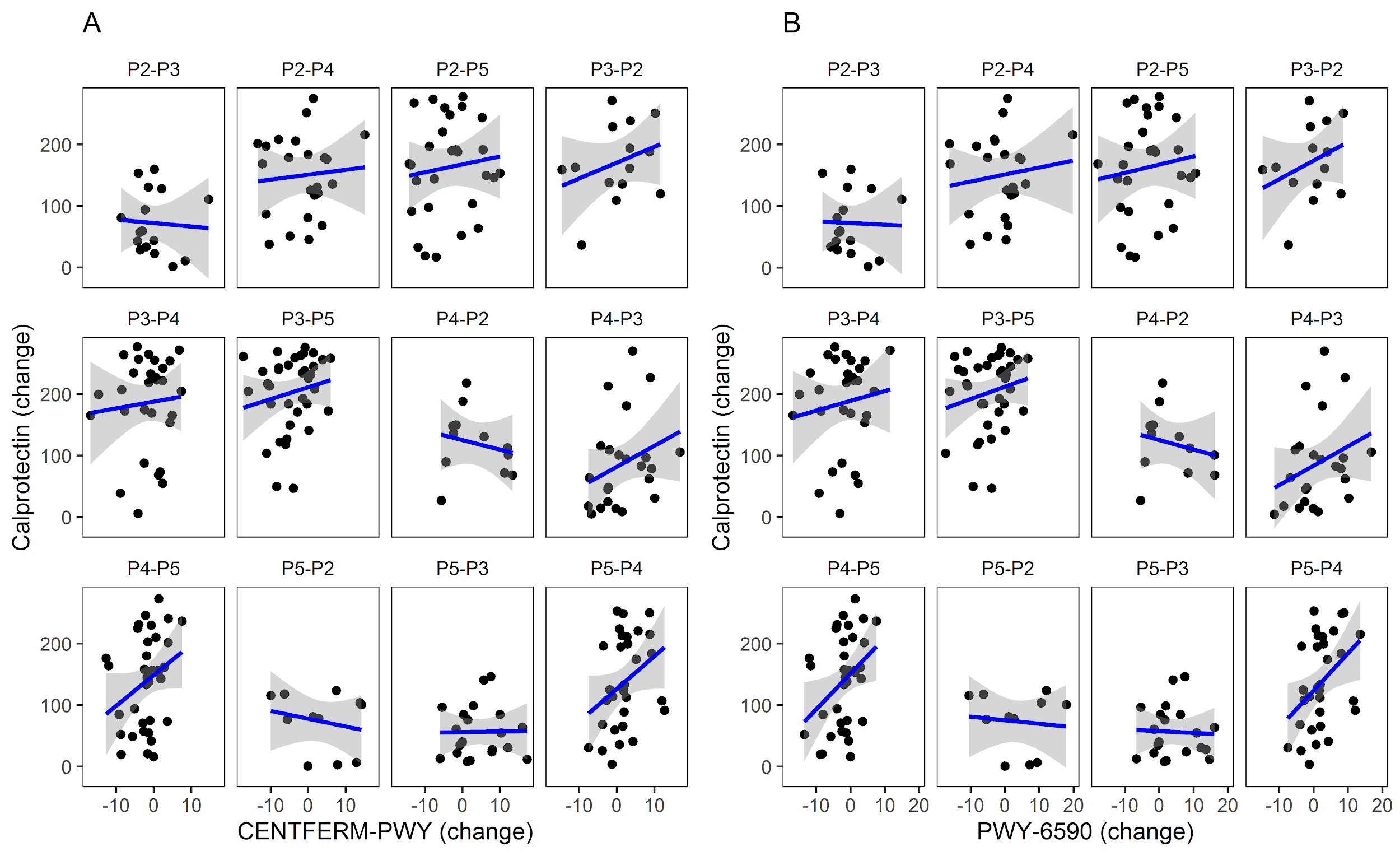
**

### Figure S12. Relationship between (changes in) gut abundance of inferred MetaCyc pathways and (changes in) calprotectin levels (HMS cohort)

**A** - common slope model illustrating relationships between CENTFERM-PWY change (centered log-ratio transformed) and calprotectin change (rank transformed), **B** - common slope model illustrating relationships between PWY-6590 change (centered log-ratio transformed) and calprotectin change (rank transformed); Likelihood ratio test (LRT) p- and Q-values were computed based on 128 Monte Carlo instances of the Dirichlet distribution, followed by center-log transform of each instance, the plots were based on the first Monte Carlo instance.

### Table S1. Repeated measures correlation of zonulin and calprotectin with microorganisms and predicted MetaCyc pathways (PMU cohort)

| **Biomarker** | **Bacterial/Pathway name** | **r** | **Taxonomic rank/MetaCyc pathway category** | **Q** |
| --- | --- | --- | --- | --- |
| Zonulin | Corynebacterium 1 | -0.38 | Genus | 0.033 |
|  | Staphylococcus | -0.39 |  | 0.028 |
|  | Lachnoclostridium | 0.45 |  | 0.014 |
|  | [Ruminococcus] gnavus group | 0.36 |  | 0.046 |
|  | [Ruminococcus] torques group | 0.40 |  | 0.026 |
| Calprotectin | Staphylococcus | 0.38 |  | 0.023 |
|  | Enterococcus | 0.40 |  | 0.019 |
|  | Anaerostipes | -0.35 |  | 0.043 |
| Zonulin | Staphylococcaceae | -0.41 | Family | 0.014 |
|  | Lachnospiraceae | 0.47 |  | 0.004 |
|  | Peptostreptococcaceae | 0.38 |  | 0.030 |
|  | Ruminococcaceae | 0.36 |  | 0.041 |
|  | Moraxellaceae | -0.41 |  | 0.022 |
| Calprotectin | Staphylococcaceae | 0.35 |  | 0.040 |
|  | Enterococcaceae | 0.36 |  | 0.038 |
|  | Ruminococcaceae | -0.34 |  | 0.046 |
| Zonulin | Coriobacteriales | 0.42 | Order | 0.006 |
|  | Bacillales | -0.52 |  | 0.0004 |
|  | Clostridiales | 0.48 |  | 0.001 |
|  | Erysipelotrichales | 0.34 |  | 0.035 |
|  | Pseudomonadales | -0.41 |  | 0.012 |
| Calprotectin | Clostridiales | -0.34 |  | 0.048 |
| Zonulin | Coriobacteriia | 0.38 | Class | 0.012 |
|  | Bacilli | -0.41 |  | 0.006 |
|  | Clostridia | 0.44 |  | 0.004 |
|  | Alphaproteobacteria | -0.33 |  | 0.036 |
| Zonulin | FUC-RHAMCAT-PWY | 0.51 | Superpathways | 0.004 |
| Zonulin | FUCCAT-PWY | 0.48 | Degradation/Utilization/Assimilation | 0.007 |
|  | PWY-6353 | 0.42 |  | 0.025 |
|  | PWY-7237 | 0.46 |  | 0.011 |
|  | SALVADEHYPOX-PWY | 0.42 |  | 0.026 |
| Zonulin | PWY-5676 | 0.40 | Generation of Precursor Metabolites and Energy | 0.044 |
|  | PWY-5677 | 0.52 |  | 0.004 |
|  | PWY-6588 | 0.49 |  | 0.006 |
|  | PWY-7003 | 0.50 |  | 0.006 |
| Calprotectin | PWY-5677 | -0.45 |  | 0.026 |
|  | PWY-6588 | -0.42 |  | 0.041 |
| Zonulin | PWY-6478 | 0.47 | Biosynthesis | 0.009 |

*Note:* r- repeated measures correlation coefficient, Q - FDR adjusted p-value

### Table S2. Repeated measures correlation of calprotectin with bacteria and MetaCyc pathway abundance in the PMU and HMS cohorts

| **PMU cohort (P2-P6)** | **HMS cohort (P2-P5)** |
| --- | --- |
| **Genus**  **Staphylococcus (0.38)**, Enterococcus (0.40), Anaerostipes (-0.36) | **Genus**  **Staphylococcus (0.34)**, Lachnospira (-0.22), Streptococcus (0.30), Rothia (0.25), Cutibacterium (0.25) |
| **Family**  **Staphylococcaceae (0.35)**, Enterococcaceae (0.36), **Ruminococcaceae (-0.34)** | **Family**  **Staphylococcaceae (0.34),**  Streptococcaceae (0.29),  **Ruminococcaceae (-0.29)**,  Lachnospiraceae (-0.34),  Micrococcaceae (0.23),  Propionibacteriaceae (0.24) |
| **Order**  **Clostridiales (-0.34)** | **Order**  **Clostridiales (-0.23)**,  Micrococcales (0.26),  Propionibacteriales (0.23),  Bacteroidales (-0.22),  Bacillales (0.32),  Lactobacillales (0.25),  Erysipelotrichales (-0.19),  Pasteurellales (-0.20) |
| **Class**  No significant correlations | **Class**  Bacilli (0.30) |
| **Phylum**  No significant correlations | **Phylum**  No significant correlations |
| **MetaCyc pathways**  PWY-5677 (-0.45), PWY-6588 (-0.42) | **MetaCyc pathways**  ARGORNPROST-PWY (0.30), GOLPDLCAT-PWY (0.32), LACTOSECAT-PWY (0.33), P125-PWY (0.33), P562-PWY (0.30), PWY-2941 (0.33), PWY-5265 (0.38), PWY-5509 (-0.30), PWY-5676 (-0.35), PWY-5910 (0.33), PWY-6269 (-0.30), PWY-6396 (0.29), PWY-7198 (-0.29), PWY-7210 (-0.30), PWY-922 (0.33) |

### Table S3. Pathway change vs Zonulin change (PMU cohort)

| No | Pathway | P (w/o int.) | P (w/ int.) | Q (w/o int.) | Q (w/ int.) |
| --- | --- | --- | --- | --- | --- |
| 1 | 1CMET2-PWY | 0.181 | 0.097 | 0.564 | 0.349 |
| 2 | 3-HYDROXYPHENYLACETATE-DEGRADATION-PWY | 0.694 | 0.292 | 0.881 | 0.523 |
| 3 | AEROBACTINSYN-PWY | 0.273 | 0.462 | 0.635 | 0.643 |
| 4 | ALL-CHORISMATE-PWY | 0.869 | 0.336 | 0.962 | 0.562 |
| 5 | ANAEROFRUCAT-PWY | 0.357 | 0.098 | 0.701 | 0.349 |
| 6 | ANAGLYCOLYSIS-PWY | 0.116 | 0.307 | 0.543 | 0.535 |
| 7 | ARG+POLYAMINE-SYN | 0.890 | 0.233 | 0.968 | 0.494 |
| 8 | ARGDEG-PWY | 0.694 | 0.408 | 0.883 | 0.605 |
| 9 | ARGORNPROST-PWY | 0.945 | 0.228 | 0.983 | 0.490 |
| 10 | ARGSYN-PWY | 0.753 | 0.045 | 0.931 | 0.240 |
| 11 | ARGSYNBSUB-PWY | 0.884 | 0.014 | 0.970 | 0.185 |
| 12 | ARO-PWY | 0.378 | 0.179 | 0.707 | 0.463 |
| 13 | ASPASN-PWY | 0.230 | 0.703 | 0.619 | 0.803 |
| 14 | AST-PWY | 0.051 | 0.036 | 0.494 | 0.221 |
| 15 | BIOTIN-BIOSYNTHESIS-PWY | 0.145 | 0.029 | 0.553 | 0.220 |
| 16 | BRANCHED-CHAIN-AA-SYN-PWY | 0.372 | 0.025 | 0.705 | 0.212 |
| 17 | CALVIN-PWY | 0.279 | 0.724 | 0.664 | 0.816 |
| 18 | CATECHOL-ORTHO-CLEAVAGE-PWY | 0.376 | 0.022 | 0.707 | 0.180 |
| 19 | CENTFERM-PWY | 0.600 | 0.228 | 0.844 | 0.488 |
| 20 | CHLOROPHYLL-SYN | 0.551 | 0.696 | 0.800 | 0.799 |
| 21 | COA-PWY | 0.185 | 0.282 | 0.566 | 0.522 |
| 22 | COBALSYN-PWY | 0.950 | 0.261 | 0.984 | 0.508 |
| 23 | COLANSYN-PWY | 0.563 | 0.195 | 0.823 | 0.474 |
| 24 | COMPLETE-ARO-PWY | 0.411 | 0.182 | 0.725 | 0.466 |
| 25 | DAPLYSINESYN-PWY | 0.220 | 0.032 | 0.607 | 0.225 |
| 26 | DENOVOPURINE2-PWY | 0.215 | 0.411 | 0.601 | 0.616 |
| 27 | DTDPRHAMSYN-PWY | 0.025 | 0.050 | 0.498 | 0.249 |
| 28 | ECASYN-PWY | 0.710 | 0.330 | 0.889 | 0.555 |
| 29 | ENTBACSYN-PWY | 0.861 | 0.534 | 0.959 | 0.694 |
| 30 | FAO-PWY | 0.305 | 0.831 | 0.674 | 0.886 |
| 31 | FASYN-ELONG-PWY | 0.758 | 0.398 | 0.934 | 0.608 |
| 32 | FASYN-INITIAL-PWY | 0.070 | 0.105 | 0.534 | 0.360 |
| 33 | FERMENTATION-PWY | 0.339 | 0.020 | 0.695 | 0.201 |
| 34 | FOLSYN-PWY | 0.085 | 0.010 | 0.538 | 0.175 |
| 35 | FUC-RHAMCAT-PWY | 0.620 | 0.887 | 0.855 | 0.923 |
| 36 | FUCCAT-PWY | 0.889 | 0.952 | 0.969 | 0.968 |
| 37 | GALACT-GLUCUROCAT-PWY | 0.168 | 0.286 | 0.562 | 0.525 |
| 38 | GALACTARDEG-PWY | 0.133 | 0.153 | 0.558 | 0.414 |
| 39 | GALACTUROCAT-PWY | 0.086 | 0.038 | 0.537 | 0.231 |
| 40 | GALLATE-DEGRADATION-I-PWY | 0.426 | 0.078 | 0.730 | 0.284 |
| 41 | GALLATE-DEGRADATION-II-PWY | 0.422 | 0.081 | 0.728 | 0.284 |
| 42 | GLCMANNANAUT-PWY | 0.729 | 0.643 | 0.919 | 0.764 |
| 43 | GLUCARDEG-PWY | 0.144 | 0.168 | 0.562 | 0.433 |
| 44 | GLUCARGALACTSUPER-PWY | 0.136 | 0.159 | 0.559 | 0.422 |
| 45 | GLUCONEO-PWY | 0.841 | 0.416 | 0.955 | 0.619 |
| 46 | GLUCOSE1PMETAB-PWY | 0.898 | 0.711 | 0.971 | 0.807 |
| 47 | GLUCUROCAT-PWY | 0.138 | 0.565 | 0.548 | 0.713 |
| 48 | GLUTORN-PWY | 0.547 | 0.008 | 0.813 | 0.162 |
| 49 | GLYCOCAT-PWY | 0.540 | 0.030 | 0.808 | 0.222 |
| 50 | GLYCOGENSYNTH-PWY | 0.876 | 0.052 | 0.968 | 0.252 |
| 51 | GLYCOL-GLYOXDEG-PWY | 0.553 | 0.006 | 0.813 | 0.124 |
| 52 | GLYCOLYSIS | 0.292 | 0.370 | 0.672 | 0.586 |
| 53 | GLYCOLYSIS-E-D | 0.363 | 0.890 | 0.703 | 0.923 |
| 54 | GLYCOLYSIS-TCA-GLYOX-BYPASS | 0.855 | 0.035 | 0.958 | 0.227 |
| 55 | GLYOXYLATE-BYPASS | 0.760 | 0.043 | 0.920 | 0.237 |
| 56 | GOLPDLCAT-PWY | 0.636 | 0.647 | 0.852 | 0.766 |
| 57 | HCAMHPDEG-PWY | 0.559 | 0.121 | 0.806 | 0.339 |
| 58 | HEME-BIOSYNTHESIS-II | 0.617 | 0.810 | 0.856 | 0.872 |
| 59 | HEMESYN2-PWY | 0.589 | 0.427 | 0.839 | 0.628 |
| 60 | HEXITOLDEGSUPER-PWY | 0.072 | 0.157 | 0.533 | 0.433 |
| 61 | HISDEG-PWY | 0.027 | 0.053 | 0.499 | 0.259 |
| 62 | HISTSYN-PWY | 0.700 | 0.040 | 0.907 | 0.235 |
| 63 | HOMOSER-METSYN-PWY | 0.107 | 0.042 | 0.541 | 0.238 |
| 64 | HSERMETANA-PWY | 0.917 | 0.001 | 0.976 | 0.099 |
| 65 | ILEUSYN-PWY | 0.378 | 0.019 | 0.707 | 0.198 |
| 66 | KDO-NAGLIPASYN-PWY | 0.033 | 0.521 | 0.491 | 0.686 |
| 67 | LACTOSECAT-PWY | 0.032 | 0.391 | 0.508 | 0.603 |
| 68 | LEU-DEG2-PWY | 0.349 | 0.774 | 0.679 | 0.850 |
| 69 | LPSSYN-PWY | 0.274 | 0.228 | 0.610 | 0.444 |
| 70 | MET-SAM-PWY | 0.101 | 0.031 | 0.540 | 0.224 |
| 71 | METH-ACETATE-PWY | 0.334 | 0.113 | 0.682 | 0.368 |
| 72 | METHGLYUT-PWY | 0.510 | 0.394 | 0.774 | 0.579 |
| 73 | METHYLGALLATE-DEGRADATION-PWY | 0.494 | 0.077 | 0.773 | 0.285 |
| 74 | NAD-BIOSYNTHESIS-II | 0.335 | 0.138 | 0.686 | 0.405 |
| 75 | NAGLIPASYN-PWY | 0.414 | 0.191 | 0.729 | 0.472 |
| 76 | NONMEVIPP-PWY | 0.112 | 0.045 | 0.542 | 0.240 |
| 77 | NONOXIPENT-PWY | 0.121 | 0.533 | 0.544 | 0.690 |
| 78 | OANTIGEN-PWY | 0.064 | 0.508 | 0.534 | 0.677 |
| 79 | ORNARGDEG-PWY | 0.683 | 0.392 | 0.878 | 0.596 |
| 80 | ORNDEG-PWY | 0.703 | 0.463 | 0.886 | 0.646 |
| 81 | P101-PWY | 0.440 | 0.099 | 0.734 | 0.315 |
| 82 | P105-PWY | 0.854 | 0.092 | 0.959 | 0.336 |
| 83 | P108-PWY | 0.601 | 0.086 | 0.845 | 0.335 |
| 84 | P122-PWY | 0.143 | 0.118 | 0.548 | 0.380 |
| 85 | P124-PWY | 0.070 | 0.123 | 0.535 | 0.387 |
| 86 | P125-PWY | 0.196 | 0.689 | 0.588 | 0.793 |
| 87 | P161-PWY | 0.202 | 0.045 | 0.585 | 0.242 |
| 88 | P162-PWY | 0.716 | 0.174 | 0.892 | 0.402 |
| 89 | P163-PWY | 0.565 | 0.889 | 0.811 | 0.925 |
| 90 | P164-PWY | 0.222 | 0.225 | 0.613 | 0.480 |
| 91 | P221-PWY | 0.009 | 0.065 | 0.380 | 0.282 |
| 92 | P23-PWY | 0.504 | 0.624 | 0.788 | 0.751 |
| 93 | P281-PWY | 0.696 | 0.319 | 0.881 | 0.531 |
| 94 | P381-PWY | 0.238 | 0.174 | 0.602 | 0.399 |
| 95 | P4-PWY | 0.305 | 0.004 | 0.678 | 0.121 |
| 96 | P42-PWY | 0.267 | 0.280 | 0.656 | 0.520 |
| 97 | P441-PWY | 0.530 | 0.739 | 0.803 | 0.825 |
| 98 | P461-PWY | 0.045 | 0.393 | 0.517 | 0.604 |
| 99 | P562-PWY | 0.031 | 0.762 | 0.476 | 0.841 |
| 100 | PANTO-PWY | 0.245 | 0.327 | 0.629 | 0.554 |
| 101 | PANTOSYN-PWY | 0.247 | 0.305 | 0.631 | 0.538 |
| **102** | **PENTOSE-P-PWY** | **0.0000091** | **0.134** | **0.003** | **0.406** |
| 103 | PEPTIDOGLYCANSYN-PWY | 0.165 | 0.294 | 0.553 | 0.529 |
| 104 | PHOSLIPSYN-PWY | 0.671 | 0.504 | 0.890 | 0.675 |
| 105 | POLYAMINSYN3-PWY | 0.783 | 0.376 | 0.927 | 0.591 |
| 106 | POLYAMSYN-PWY | 0.841 | 0.332 | 0.952 | 0.560 |
| 107 | POLYISOPRENSYN-PWY | 0.866 | 0.483 | 0.963 | 0.663 |
| 108 | PPGPPMET-PWY | 0.062 | 0.216 | 0.500 | 0.473 |
| 109 | PROTOCATECHUATE-ORTHO-CLEAVAGE-PWY | 0.712 | 0.014 | 0.891 | 0.162 |
| 110 | PRPP-PWY | 0.937 | 0.826 | 0.981 | 0.883 |
| 111 | PWY-1269 | 0.461 | 0.341 | 0.757 | 0.567 |
| 112 | PWY-1861 | 0.099 | 0.402 | 0.540 | 0.610 |
| 113 | PWY-2941 | 0.543 | 0.227 | 0.811 | 0.489 |
| 114 | PWY-2942 | 0.147 | 0.254 | 0.548 | 0.504 |
| 115 | PWY-3001 | 0.302 | 0.034 | 0.677 | 0.227 |
| 116 | PWY-3781 | 0.727 | 0.766 | 0.898 | 0.844 |
| 117 | PWY-4984 | 0.838 | 0.636 | 0.955 | 0.759 |
| 118 | PWY-5005 | 0.746 | 0.464 | 0.929 | 0.653 |
| 119 | PWY-5022 | 0.179 | 0.045 | 0.566 | 0.243 |
| 120 | PWY-5028 | 0.202 | 0.414 | 0.575 | 0.606 |
| 121 | PWY-5088 | 0.342 | 0.846 | 0.674 | 0.897 |
| 122 | PWY-5097 | 0.056 | 0.199 | 0.533 | 0.475 |
| 123 | PWY-5100 | 0.310 | 0.651 | 0.681 | 0.770 |
| 124 | PWY-5101 | 0.569 | 0.033 | 0.824 | 0.226 |
| 125 | PWY-5103 | 0.376 | 0.023 | 0.707 | 0.207 |
| 126 | PWY-5104 | 0.919 | 0.225 | 0.977 | 0.488 |
| 127 | PWY-5121 | 0.084 | 0.052 | 0.537 | 0.251 |
| 128 | PWY-5154 | 0.141 | 0.036 | 0.547 | 0.228 |
| 129 | PWY-5177 | 0.840 | 0.819 | 0.957 | 0.879 |
| 130 | PWY-5178 | 0.207 | 0.117 | 0.598 | 0.333 |
| 131 | PWY-5180 | 0.131 | 0.329 | 0.555 | 0.559 |
| 132 | PWY-5181 | 0.119 | 0.595 | 0.542 | 0.733 |
| 133 | PWY-5182 | 0.123 | 0.337 | 0.552 | 0.563 |
| 134 | PWY-5188 | 0.143 | 0.085 | 0.552 | 0.331 |
| 135 | PWY-5189 | 0.155 | 0.087 | 0.557 | 0.334 |
| 136 | PWY-5198 | 0.401 | 0.300 | 0.710 | 0.510 |
| 137 | PWY-5265 | 0.373 | 0.084 | 0.706 | 0.314 |
| 138 | PWY-5304 | 0.036 | 0.081 | 0.513 | 0.320 |
| 139 | PWY-5345 | 0.830 | 0.479 | 0.958 | 0.662 |
| 140 | PWY-5347 | 0.065 | 0.046 | 0.534 | 0.241 |
| 141 | PWY-5384 | 0.176 | 0.602 | 0.561 | 0.736 |
| 142 | PWY-5415 | 0.447 | 0.278 | 0.745 | 0.523 |
| 143 | PWY-5417 | 0.243 | 0.021 | 0.625 | 0.176 |
| 144 | PWY-5431 | 0.237 | 0.018 | 0.621 | 0.170 |
| 145 | PWY-5484 | 0.162 | 0.205 | 0.552 | 0.478 |
| 146 | PWY-5505 | 0.221 | 0.343 | 0.607 | 0.566 |
| 147 | PWY-5507 | 0.241 | 0.172 | 0.606 | 0.396 |
| 148 | PWY-5509 | 0.875 | 0.396 | 0.968 | 0.606 |
| 149 | PWY-5531 | 0.544 | 0.643 | 0.799 | 0.764 |
| 150 | PWY-5532 | 0.209 | 0.276 | 0.566 | 0.495 |
| 151 | PWY-5659 | 0.955 | 0.525 | 0.986 | 0.688 |
| 152 | PWY-5667 | 0.351 | 0.527 | 0.700 | 0.687 |
| 153 | PWY-5676 | 0.460 | 0.946 | 0.751 | 0.963 |
| 154 | PWY-5677 | 0.086 | 0.769 | 0.512 | 0.846 |
| 155 | PWY-5686 | 0.142 | 0.252 | 0.547 | 0.503 |
| 156 | PWY-5695 | 0.045 | 0.574 | 0.529 | 0.718 |
| 157 | PWY-5705 | 0.026 | 0.588 | 0.455 | 0.729 |
| 158 | PWY-5747 | 0.502 | 0.327 | 0.773 | 0.549 |
| 159 | PWY-5837 | 0.170 | 0.393 | 0.563 | 0.604 |
| 160 | PWY-5838 | 0.421 | 0.429 | 0.737 | 0.629 |
| 161 | PWY-5840 | 0.378 | 0.212 | 0.714 | 0.482 |
| 162 | PWY-5845 | 0.146 | 0.685 | 0.556 | 0.791 |
| 163 | PWY-5850 | 0.153 | 0.692 | 0.558 | 0.795 |
| 164 | PWY-5855 | 0.544 | 0.024 | 0.808 | 0.190 |
| 165 | PWY-5856 | 0.557 | 0.023 | 0.816 | 0.186 |
| 166 | PWY-5857 | 0.565 | 0.026 | 0.821 | 0.191 |
| 167 | PWY-5860 | 0.153 | 0.598 | 0.562 | 0.736 |
| 168 | PWY-5861 | 0.470 | 0.530 | 0.767 | 0.690 |
| 169 | PWY-5862 | 0.151 | 0.599 | 0.560 | 0.736 |
| 170 | PWY-5863 | 0.181 | 0.391 | 0.571 | 0.603 |
| 171 | PWY-5896 | 0.156 | 0.691 | 0.561 | 0.795 |
| 172 | PWY-5897 | 0.358 | 0.232 | 0.708 | 0.491 |
| 173 | PWY-5898 | 0.358 | 0.230 | 0.707 | 0.490 |
| 174 | PWY-5899 | 0.359 | 0.232 | 0.708 | 0.491 |
| 175 | PWY-5910 | 0.374 | 0.712 | 0.713 | 0.808 |
| 176 | PWY-5913 | 0.143 | 0.012 | 0.548 | 0.179 |
| 177 | PWY-5918 | 0.503 | 0.632 | 0.787 | 0.756 |
| 178 | PWY-5920 | 0.722 | 0.488 | 0.909 | 0.665 |
| 179 | PWY-5971 | 0.024 | 0.204 | 0.494 | 0.477 |
| 180 | PWY-5973 | 0.848 | 0.790 | 0.964 | 0.861 |
| 181 | PWY-5989 | 0.071 | 0.097 | 0.534 | 0.350 |
| 182 | PWY-6071 | 0.393 | 0.257 | 0.717 | 0.500 |
| 183 | PWY-6121 | 0.138 | 0.300 | 0.547 | 0.531 |
| 184 | PWY-6122 | 0.147 | 0.308 | 0.548 | 0.536 |
| 185 | PWY-6123 | 0.117 | 0.271 | 0.543 | 0.514 |
| 186 | PWY-6125 | 0.300 | 0.694 | 0.677 | 0.798 |
| 187 | PWY-6126 | 0.057 | 0.197 | 0.533 | 0.474 |
| 188 | PWY-6147 | 0.233 | 0.003 | 0.623 | 0.116 |
| 189 | PWY-6151 | 0.089 | 0.476 | 0.538 | 0.660 |
| 190 | PWY-6163 | 0.265 | 0.197 | 0.654 | 0.474 |
| 191 | PWY-6165 | 0.295 | 0.536 | 0.607 | 0.685 |
| 192 | PWY-6182 | 0.520 | 0.123 | 0.786 | 0.325 |
| 193 | PWY-6185 | 0.548 | 0.348 | 0.801 | 0.551 |
| 194 | PWY-621 | 0.605 | 0.487 | 0.846 | 0.667 |
| 195 | PWY-6269 | 0.873 | 0.394 | 0.967 | 0.604 |
| 196 | PWY-6277 | 0.148 | 0.308 | 0.548 | 0.536 |
| 197 | PWY-6282 | 0.072 | 0.103 | 0.534 | 0.357 |
| 198 | PWY-6317 | 0.367 | 0.157 | 0.705 | 0.442 |
| 199 | PWY-6353 | 0.780 | 0.776 | 0.941 | 0.851 |
| 200 | PWY-6383 | 0.676 | 0.193 | 0.875 | 0.447 |
| 201 | PWY-6385 | 0.134 | 0.243 | 0.547 | 0.499 |
| 202 | PWY-6386 | 0.178 | 0.258 | 0.560 | 0.506 |
| 203 | PWY-6387 | 0.166 | 0.300 | 0.554 | 0.532 |
| 204 | PWY-6396 | 0.464 | 0.832 | 0.762 | 0.886 |
| 205 | PWY-6397 | 0.504 | 0.204 | 0.772 | 0.424 |
| 206 | PWY-6467 | 0.849 | 0.475 | 0.962 | 0.660 |
| 207 | PWY-6470 | 0.877 | 0.358 | 0.968 | 0.579 |
| 208 | PWY-6471 | 0.325 | 0.036 | 0.689 | 0.229 |
| 209 | PWY-6478 | 0.136 | 0.922 | 0.557 | 0.948 |
| 210 | PWY-6507 | 0.455 | 0.200 | 0.753 | 0.475 |
| 211 | PWY-6519 | 0.162 | 0.026 | 0.559 | 0.214 |
| **212** | **PWY-6545** | **0.653** | **0.000041** | **0.878** | **0.014** |
| 213 | PWY-6562 | 0.780 | 0.808 | 0.925 | 0.872 |
| 214 | PWY-6572 | 0.600 | 0.728 | 0.829 | 0.819 |
| 215 | PWY-6588 | 0.150 | 0.081 | 0.565 | 0.314 |
| 216 | PWY-6590 | 0.558 | 0.234 | 0.820 | 0.492 |
| 217 | PWY-6608 | 0.938 | 0.516 | 0.981 | 0.683 |
| 218 | PWY-6609 | 0.778 | 0.854 | 0.939 | 0.900 |
| 219 | PWY-6612 | 0.087 | 0.005 | 0.538 | 0.136 |
| 220 | PWY-6628 | 0.427 | 0.483 | 0.735 | 0.664 |
| 221 | PWY-6629 | 0.262 | 0.145 | 0.606 | 0.358 |
| 222 | PWY-6630 | 0.436 | 0.490 | 0.741 | 0.667 |
| 223 | PWY-6690 | 0.539 | 0.117 | 0.796 | 0.333 |
| 224 | PWY-6700 | 0.851 | 0.621 | 0.959 | 0.749 |
| 225 | PWY-6703 | 0.215 | 0.200 | 0.601 | 0.477 |
| 226 | PWY-6708 | 0.563 | 0.025 | 0.819 | 0.191 |
| 227 | PWY-6737 | 0.300 | 0.017 | 0.676 | 0.194 |
| 228 | PWY-6749 | 0.778 | 0.058 | 0.923 | 0.271 |
| 229 | PWY-6876 | 0.434 | 0.370 | 0.737 | 0.582 |
| 230 | PWY-6891 | 0.197 | 0.130 | 0.586 | 0.399 |
| 231 | PWY-6892 | 0.909 | 0.030 | 0.975 | 0.222 |
| 232 | PWY-6895 | 0.818 | 0.224 | 0.950 | 0.487 |
| 233 | PWY-6897 | 0.797 | 0.329 | 0.944 | 0.554 |
| **234** | **PWY-6901** | **0.003** | **0.001** | **0.290** | **0.087** |
| 235 | PWY-6906 | 0.294 | 0.569 | 0.607 | 0.708 |
| 236 | PWY-6969 | 0.687 | 0.120 | 0.898 | 0.380 |
| 237 | PWY-7003 | 0.038 | 0.133 | 0.496 | 0.396 |
| 238 | PWY-7007 | 0.489 | 0.116 | 0.764 | 0.334 |
| 239 | PWY-7013 | 0.694 | 0.144 | 0.881 | 0.416 |
| 240 | PWY-7094 | 0.211 | 0.473 | 0.597 | 0.650 |
| 241 | PWY-7111 | 0.373 | 0.016 | 0.706 | 0.192 |
| 242 | PWY-7159 | 0.567 | 0.654 | 0.811 | 0.770 |
| 243 | PWY-7184 | 0.562 | 0.327 | 0.821 | 0.554 |
| 244 | PWY-7187 | 0.090 | 0.192 | 0.538 | 0.471 |
| 245 | PWY-7196 | 0.603 | 0.825 | 0.845 | 0.882 |
| 246 | PWY-7197 | 0.518 | 0.787 | 0.797 | 0.858 |
| 247 | PWY-7198 | 0.245 | 0.343 | 0.624 | 0.564 |
| 248 | PWY-7199 | 0.354 | 0.945 | 0.700 | 0.964 |
| 249 | PWY-7200 | 0.697 | 0.511 | 0.905 | 0.680 |
| 250 | PWY-7208 | 0.334 | 0.399 | 0.693 | 0.608 |
| 251 | PWY-7210 | 0.263 | 0.372 | 0.635 | 0.584 |
| 252 | PWY-7211 | 0.474 | 0.577 | 0.771 | 0.719 |
| 253 | PWY-7219 | 0.165 | 0.300 | 0.553 | 0.532 |
| 254 | PWY-7220 | 0.011 | 0.181 | 0.423 | 0.466 |
| 255 | PWY-7221 | 0.102 | 0.270 | 0.541 | 0.514 |
| 256 | PWY-7222 | 0.011 | 0.181 | 0.423 | 0.466 |
| 257 | PWY-7228 | 0.311 | 0.714 | 0.682 | 0.809 |
| 258 | PWY-7229 | 0.069 | 0.230 | 0.535 | 0.492 |
| 259 | PWY-7234 | 0.030 | 0.262 | 0.507 | 0.508 |
| 260 | PWY-7237 | 0.006 | 0.668 | 0.351 | 0.780 |
| 261 | PWY-7242 | 0.084 | 0.078 | 0.536 | 0.316 |
| 262 | PWY-7254 | 0.119 | 0.326 | 0.544 | 0.553 |
| 263 | PWY-7255 | 0.541 | 0.279 | 0.795 | 0.500 |
| 264 | PWY-7295 | 0.427 | 0.625 | 0.715 | 0.749 |
| 265 | PWY-7315 | 0.553 | 0.944 | 0.816 | 0.963 |
| 266 | PWY-7323 | 0.828 | 0.109 | 0.957 | 0.365 |
| 267 | PWY-7328 | 0.709 | 0.428 | 0.909 | 0.628 |
| 268 | PWY-7332 | 0.578 | 0.122 | 0.816 | 0.340 |
| 269 | PWY-7376 | 0.435 | 0.129 | 0.731 | 0.348 |
| 270 | PWY-7377 | 0.624 | 0.872 | 0.859 | 0.912 |
| 271 | PWY-7392 | 0.361 | 0.515 | 0.708 | 0.681 |
| 272 | PWY-7400 | 0.759 | 0.045 | 0.933 | 0.240 |
| 273 | PWY-7431 | 0.560 | 0.398 | 0.805 | 0.596 |
| 274 | PWY-7446 | 0.346 | 0.501 | 0.681 | 0.670 |
| 275 | PWY-7456 | 0.069 | 0.019 | 0.523 | 0.184 |
| 276 | PWY-7539 | 0.520 | 0.099 | 0.798 | 0.351 |
| 277 | PWY-7560 | 0.112 | 0.045 | 0.542 | 0.240 |
| 278 | PWY-7616 | 0.533 | 0.331 | 0.791 | 0.536 |
| 279 | PWY-7663 | 0.923 | 0.879 | 0.977 | 0.916 |
| 280 | PWY-7664 | 0.068 | 0.104 | 0.533 | 0.358 |
| 281 | PWY-841 | 0.285 | 0.521 | 0.668 | 0.685 |
| 282 | PWY-922 | 0.394 | 0.700 | 0.722 | 0.801 |
| 283 | PWY0-1061 | 0.253 | 0.079 | 0.644 | 0.319 |
| 284 | PWY0-1241 | 0.778 | 0.992 | 0.938 | 0.993 |
| 285 | PWY0-1261 | 0.597 | 0.571 | 0.844 | 0.717 |
| 286 | PWY0-1277 | 0.490 | 0.056 | 0.769 | 0.243 |
| 287 | PWY0-1296 | 0.536 | 0.398 | 0.807 | 0.608 |
| 288 | PWY0-1297 | 0.752 | 0.299 | 0.932 | 0.534 |
| 289 | PWY0-1298 | 0.890 | 0.222 | 0.970 | 0.487 |
| 290 | PWY0-1319 | 0.352 | 0.527 | 0.700 | 0.687 |
| 291 | PWY0-1338 | 0.717 | 0.408 | 0.892 | 0.605 |
| 292 | PWY0-1415 | 0.095 | 0.415 | 0.539 | 0.616 |
| 293 | PWY0-1479 | 0.344 | 0.448 | 0.696 | 0.640 |
| 294 | PWY0-1533 | 0.117 | 0.128 | 0.547 | 0.384 |
| 295 | PWY0-1586 | 0.964 | 0.175 | 0.988 | 0.463 |
| 296 | PWY0-162 | 0.418 | 0.573 | 0.729 | 0.715 |
| 297 | PWY0-166 | 0.451 | 0.149 | 0.750 | 0.426 |
| 298 | PWY0-321 | 0.333 | 0.397 | 0.680 | 0.600 |
| 299 | PWY0-41 | 0.051 | 0.587 | 0.465 | 0.726 |
| 300 | PWY0-42 | 0.347 | 0.496 | 0.674 | 0.663 |
| 301 | PWY0-781 | 0.479 | 0.005 | 0.769 | 0.126 |
| **302** | **PWY0-845** | **0.841** | **0.002** | **0.958** | **0.106** |
| 303 | PWY0-862 | 0.940 | 0.230 | 0.982 | 0.491 |
| 304 | PWY1G-0 | 0.471 | 0.735 | 0.754 | 0.824 |
| 305 | PWY490-3 | 0.318 | 0.535 | 0.683 | 0.695 |
| 306 | PWY4FS-7 | 0.801 | 0.223 | 0.944 | 0.487 |
| 307 | PWY4FS-8 | 0.801 | 0.222 | 0.944 | 0.487 |
| 308 | PWYG-321 | 0.070 | 0.104 | 0.534 | 0.359 |
| 309 | PYRIDNUCSAL-PWY | 0.310 | 0.012 | 0.681 | 0.183 |
| 310 | PYRIDNUCSYN-PWY | 0.170 | 0.016 | 0.557 | 0.193 |
| 311 | PYRIDOXSYN-PWY | 0.905 | 0.002 | 0.973 | 0.108 |
| 312 | REDCITCYC | 0.931 | 0.675 | 0.980 | 0.785 |
| 313 | RHAMCAT-PWY | 0.482 | 0.167 | 0.772 | 0.449 |
| 314 | RIBOSYN2-PWY | 0.381 | 0.248 | 0.709 | 0.500 |
| 315 | RUMP-PWY | 0.051 | 0.416 | 0.532 | 0.620 |
| 316 | SALVADEHYPOX-PWY | 0.559 | 0.987 | 0.821 | 0.991 |
| 317 | SER-GLYSYN-PWY | 0.146 | 0.022 | 0.548 | 0.205 |
| 318 | SO4ASSIM-PWY | 0.283 | 0.499 | 0.663 | 0.673 |
| 319 | SULFATE-CYS-PWY | 0.373 | 0.327 | 0.713 | 0.556 |
| 320 | TCA | 0.955 | 0.066 | 0.986 | 0.289 |
| 321 | TCA-GLYOX-BYPASS | 0.827 | 0.035 | 0.947 | 0.225 |
| 322 | TEICHOICACID-PWY | 0.835 | 0.565 | 0.956 | 0.712 |
| 323 | THISYN-PWY | 0.438 | 0.101 | 0.742 | 0.355 |
| 324 | THREOCAT-PWY | 0.802 | 0.213 | 0.935 | 0.471 |
| 325 | THRESYN-PWY | 0.254 | 0.045 | 0.646 | 0.241 |
| 326 | TRNA-CHARGING-PWY | 0.264 | 0.243 | 0.653 | 0.499 |
| 327 | TRPSYN-PWY | 0.100 | 0.189 | 0.541 | 0.472 |
| 328 | TYRFUMCAT-PWY | 0.462 | 0.664 | 0.744 | 0.776 |
| 329 | UBISYN-PWY | 0.633 | 0.019 | 0.856 | 0.171 |
| 330 | UDPNAGSYN-PWY | 0.438 | 0.211 | 0.741 | 0.482 |
| 331 | VALDEG-PWY | 0.589 | 0.414 | 0.821 | 0.599 |
| 332 | VALSYN-PWY | 0.378 | 0.019 | 0.707 | 0.198 |

###

### Table S4. Pathway change vs Calprotectin change (PMU cohort)

| No | Taxon | P (w/o int.) | P (w/ int.) | Q (w/o int.) | Q ( w/ int.) |
| --- | --- | --- | --- | --- | --- |
| 1 | 1CMET2-PWY | 0.125 | 0.439 | 0.484 | 0.993 |
| 2 | 3-HYDROXYPHENYLACETATE-DEGRADATION-PWY | 0.560 | 0.684 | 0.813 | 0.993 |
| 3 | AEROBACTINSYN-PWY | 0.256 | 0.840 | 0.561 | 0.995 |
| 4 | ALL-CHORISMATE-PWY | 0.546 | 0.910 | 0.813 | 0.994 |
| 5 | ANAEROFRUCAT-PWY | 0.036 | 0.594 | 0.299 | 0.993 |
| 6 | ANAGLYCOLYSIS-PWY | 0.466 | 0.831 | 0.779 | 0.993 |
| 7 | ARG+POLYAMINE-SYN | 0.404 | 0.065 | 0.756 | 0.993 |
| 8 | ARGDEG-PWY | 0.532 | 0.681 | 0.800 | 0.993 |
| 9 | ARGORNPROST-PWY | 0.367 | 0.898 | 0.741 | 0.993 |
| 10 | ARGSYN-PWY | 0.249 | 0.842 | 0.648 | 0.993 |
| 11 | ARGSYNBSUB-PWY | 0.334 | 0.829 | 0.724 | 0.993 |
| 12 | ARO-PWY | 0.856 | 0.887 | 0.930 | 0.993 |
| 13 | ASPASN-PWY | 0.237 | 0.533 | 0.636 | 0.993 |
| 14 | AST-PWY | 0.528 | 0.847 | 0.796 | 0.993 |
| 15 | BIOTIN-BIOSYNTHESIS-PWY | 0.739 | 0.919 | 0.894 | 0.993 |
| 16 | BRANCHED-CHAIN-AA-SYN-PWY | 0.905 | 0.252 | 0.951 | 0.993 |
| 17 | CALVIN-PWY | 0.044 | 0.748 | 0.317 | 0.993 |
| 18 | CATECHOL-ORTHO-CLEAVAGE-PWY | 0.436 | 0.884 | 0.747 | 0.993 |
| **19** | **CENTFERM-PWY** | **0.00033** | **0.438** | **0.048** | **0.993** |
| 20 | CHLOROPHYLL-SYN | 0.091 | 0.495 | 0.333 | 0.993 |
| 21 | COA-PWY | 0.216 | 0.464 | 0.608 | 0.993 |
| 22 | COBALSYN-PWY | 0.117 | 0.703 | 0.471 | 0.993 |
| 23 | COLANSYN-PWY | 0.597 | 0.973 | 0.849 | 0.997 |
| 24 | COMPLETE-ARO-PWY | 0.859 | 0.868 | 0.932 | 0.993 |
| 25 | DAPLYSINESYN-PWY | 0.934 | 0.906 | 0.964 | 0.993 |
| 26 | DENOVOPURINE2-PWY | 0.036 | 0.718 | 0.298 | 0.993 |
| 27 | DTDPRHAMSYN-PWY | 0.504 | 0.912 | 0.806 | 0.993 |
| 28 | ECASYN-PWY | 0.525 | 0.741 | 0.795 | 0.993 |
| 29 | ENTBACSYN-PWY | 0.788 | 0.346 | 0.911 | 0.993 |
| 30 | FAO-PWY | 0.033 | 0.310 | 0.281 | 0.993 |
| 31 | FASYN-ELONG-PWY | 0.003 | 0.638 | 0.146 | 0.993 |
| 32 | FASYN-INITIAL-PWY | 0.583 | 0.812 | 0.842 | 0.993 |
| 33 | FERMENTATION-PWY | 0.072 | 0.817 | 0.391 | 0.993 |
| 34 | FOLSYN-PWY | 0.673 | 0.407 | 0.875 | 0.993 |
| 35 | FUC-RHAMCAT-PWY | 0.134 | 0.558 | 0.495 | 0.993 |
| 36 | FUCCAT-PWY | 0.315 | 0.742 | 0.707 | 0.993 |
| 37 | GALACT-GLUCUROCAT-PWY | 0.014 | 0.844 | 0.209 | 0.993 |
| 38 | GALACTARDEG-PWY | 0.226 | 0.045 | 0.600 | 0.993 |
| 39 | GALACTUROCAT-PWY | 0.010 | 0.792 | 0.192 | 0.993 |
| 40 | GALLATE-DEGRADATION-I-PWY | 0.395 | 0.439 | 0.692 | 0.993 |
| 41 | GALLATE-DEGRADATION-II-PWY | 0.377 | 0.441 | 0.679 | 0.993 |
| 42 | GLCMANNANAUT-PWY | 0.399 | 0.684 | 0.752 | 0.993 |
| 43 | GLUCARDEG-PWY | 0.360 | 0.044 | 0.715 | 0.993 |
| 44 | GLUCARGALACTSUPER-PWY | 0.227 | 0.046 | 0.603 | 0.993 |
| 45 | GLUCONEO-PWY | 0.015 | 0.463 | 0.216 | 0.993 |
| 46 | GLUCOSE1PMETAB-PWY | 0.008 | 0.871 | 0.182 | 0.993 |
| 47 | GLUCUROCAT-PWY | 0.007 | 0.843 | 0.180 | 0.993 |
| 48 | GLUTORN-PWY | 0.281 | 0.345 | 0.681 | 0.993 |
| 49 | GLYCOCAT-PWY | 0.539 | 0.252 | 0.825 | 0.993 |
| 50 | GLYCOGENSYNTH-PWY | 0.610 | 0.827 | 0.853 | 0.993 |
| 51 | GLYCOL-GLYOXDEG-PWY | 0.686 | 0.481 | 0.873 | 0.993 |
| 52 | GLYCOLYSIS | 0.038 | 0.352 | 0.301 | 0.993 |
| 53 | GLYCOLYSIS-E-D | 0.009 | 0.938 | 0.189 | 0.994 |
| 54 | GLYCOLYSIS-TCA-GLYOX-BYPASS | 0.334 | 0.917 | 0.716 | 0.993 |
| 55 | GLYOXYLATE-BYPASS | 0.511 | 0.881 | 0.801 | 0.993 |
| 56 | GOLPDLCAT-PWY | 0.601 | 0.550 | 0.833 | 0.993 |
| 57 | HCAMHPDEG-PWY | 0.668 | 0.668 | 0.861 | 0.993 |
| 58 | HEME-BIOSYNTHESIS-II | 0.073 | 0.364 | 0.392 | 0.993 |
| 59 | HEMESYN2-PWY | 0.009 | 0.237 | 0.192 | 0.993 |
| 60 | HEXITOLDEGSUPER-PWY | 0.868 | 0.278 | 0.938 | 0.993 |
| 61 | HISDEG-PWY | 0.212 | 0.792 | 0.604 | 0.993 |
| 62 | HISTSYN-PWY | 0.724 | 0.212 | 0.889 | 0.993 |
| 63 | HOMOSER-METSYN-PWY | 0.377 | 0.284 | 0.742 | 0.993 |
| 64 | HSERMETANA-PWY | 0.459 | 0.734 | 0.781 | 0.993 |
| 65 | ILEUSYN-PWY | 0.822 | 0.221 | 0.920 | 0.993 |
| 66 | KDO-NAGLIPASYN-PWY | 0.805 | 0.842 | 0.917 | 0.993 |
| 67 | LACTOSECAT-PWY | 0.890 | 0.865 | 0.949 | 0.993 |
| 68 | LEU-DEG2-PWY | 0.485 | 0.360 | 0.734 | 0.985 |
| 69 | LPSSYN-PWY | 0.313 | 0.433 | 0.584 | 0.989 |
| 70 | MET-SAM-PWY | 0.572 | 0.286 | 0.839 | 0.993 |
| 71 | METH-ACETATE-PWY | 0.623 | 0.827 | 0.837 | 0.994 |
| 72 | METHGLYUT-PWY | 0.387 | 0.473 | 0.653 | 0.987 |
| 73 | METHYLGALLATE-DEGRADATION-PWY | 0.314 | 0.496 | 0.631 | 0.993 |
| 74 | NAD-BIOSYNTHESIS-II | 0.540 | 0.506 | 0.818 | 0.993 |
| 75 | NAGLIPASYN-PWY | 0.226 | 0.712 | 0.620 | 0.993 |
| 76 | NONMEVIPP-PWY | 0.628 | 0.232 | 0.857 | 0.993 |
| 77 | NONOXIPENT-PWY | 0.660 | 0.682 | 0.865 | 0.993 |
| 78 | OANTIGEN-PWY | 0.350 | 0.779 | 0.731 | 0.993 |
| 79 | ORNARGDEG-PWY | 0.548 | 0.685 | 0.809 | 0.993 |
| 80 | ORNDEG-PWY | 0.394 | 0.655 | 0.723 | 0.993 |
| 81 | P101-PWY | 0.524 | 0.432 | 0.771 | 0.993 |
| 82 | P105-PWY | 0.356 | 0.839 | 0.732 | 0.993 |
| 83 | P108-PWY | 0.049 | 0.817 | 0.333 | 0.993 |
| 84 | P122-PWY | 0.824 | 0.362 | 0.921 | 0.993 |
| 85 | P124-PWY | 0.577 | 0.444 | 0.843 | 0.993 |
| 86 | P125-PWY | 0.179 | 0.904 | 0.559 | 0.993 |
| 87 | P161-PWY | 0.958 | 1.000 | 0.978 | 1.000 |
| 88 | P162-PWY | 0.171 | 0.344 | 0.483 | 0.993 |
| 89 | P163-PWY | 0.018 | 0.533 | 0.179 | 0.993 |
| 90 | P164-PWY | 0.456 | 0.872 | 0.780 | 0.993 |
| 91 | P221-PWY | 0.693 | 0.838 | 0.877 | 0.993 |
| 92 | P23-PWY | 0.072 | 0.959 | 0.389 | 0.995 |
| 93 | P281-PWY | 0.355 | 0.763 | 0.660 | 0.994 |
| 94 | P381-PWY | 0.550 | 0.466 | 0.790 | 0.993 |
| 95 | P4-PWY | 0.741 | 0.489 | 0.895 | 0.993 |
| 96 | P42-PWY | 0.142 | 0.578 | 0.511 | 0.993 |
| 97 | P441-PWY | 0.789 | 0.987 | 0.911 | 0.999 |
| 98 | P461-PWY | 0.730 | 0.029 | 0.892 | 0.993 |
| 99 | P562-PWY | 0.155 | 0.261 | 0.512 | 0.993 |
| 100 | PANTO-PWY | 0.131 | 0.731 | 0.494 | 0.993 |
| 101 | PANTOSYN-PWY | 0.099 | 0.757 | 0.440 | 0.993 |
| 102 | PENTOSE-P-PWY | 0.115 | 0.197 | 0.468 | 0.993 |
| 103 | PEPTIDOGLYCANSYN-PWY | 0.446 | 0.623 | 0.768 | 0.993 |
| 104 | PHOSLIPSYN-PWY | 0.229 | 0.831 | 0.621 | 0.993 |
| 105 | POLYAMINSYN3-PWY | 0.321 | 0.379 | 0.697 | 0.993 |
| 106 | POLYAMSYN-PWY | 0.191 | 0.132 | 0.573 | 0.993 |
| 107 | POLYISOPRENSYN-PWY | 0.485 | 0.244 | 0.791 | 0.993 |
| 108 | PPGPPMET-PWY | 0.690 | 0.738 | 0.874 | 0.993 |
| 109 | PROTOCATECHUATE-ORTHO-CLEAVAGE-PWY | 0.331 | 0.691 | 0.686 | 0.993 |
| 110 | PRPP-PWY | 0.111 | 0.145 | 0.458 | 0.993 |
| 111 | PWY-1269 | 0.257 | 0.715 | 0.657 | 0.993 |
| 112 | PWY-1861 | 0.236 | 0.150 | 0.630 | 0.993 |
| 113 | PWY-2941 | 0.662 | 0.749 | 0.868 | 0.993 |
| 114 | PWY-2942 | 0.477 | 0.565 | 0.785 | 0.993 |
| 115 | PWY-3001 | 0.933 | 0.251 | 0.964 | 0.993 |
| 116 | PWY-3781 | 0.707 | 0.742 | 0.877 | 0.993 |
| 117 | PWY-4984 | 0.006 | 0.392 | 0.171 | 0.993 |
| 118 | PWY-5005 | 0.399 | 0.848 | 0.753 | 0.993 |
| 119 | PWY-5022 | 0.131 | 0.080 | 0.491 | 0.993 |
| 120 | PWY-5028 | 0.228 | 0.517 | 0.521 | 0.993 |
| 121 | PWY-5088 | 0.064 | 0.714 | 0.279 | 0.994 |
| 122 | PWY-5097 | 0.227 | 0.535 | 0.619 | 0.993 |
| 123 | PWY-5100 | 0.673 | 0.876 | 0.870 | 0.993 |
| 124 | PWY-5101 | 0.814 | 0.255 | 0.918 | 0.993 |
| 125 | PWY-5103 | 0.875 | 0.234 | 0.937 | 0.993 |
| 126 | PWY-5104 | 0.010 | 0.680 | 0.195 | 0.993 |
| 127 | PWY-5121 | 0.647 | 0.248 | 0.862 | 0.993 |
| 128 | PWY-5154 | 0.770 | 0.209 | 0.904 | 0.993 |
| 129 | PWY-5177 | 0.044 | 0.343 | 0.314 | 0.993 |
| 130 | PWY-5178 | 0.631 | 0.894 | 0.839 | 0.994 |
| 131 | PWY-5180 | 0.123 | 0.230 | 0.467 | 0.993 |
| 132 | PWY-5181 | 0.289 | 0.547 | 0.608 | 0.993 |
| 133 | PWY-5182 | 0.129 | 0.234 | 0.474 | 0.993 |
| 134 | PWY-5188 | 0.046 | 0.181 | 0.331 | 0.993 |
| 135 | PWY-5189 | 0.056 | 0.215 | 0.356 | 0.993 |
| 136 | PWY-5198 | 0.491 | 0.837 | 0.743 | 0.994 |
| 137 | PWY-5265 | 0.025 | 0.346 | 0.241 | 0.993 |
| 138 | PWY-5304 | 0.066 | 0.826 | 0.377 | 0.993 |
| 139 | PWY-5345 | 0.209 | 0.669 | 0.603 | 0.993 |
| 140 | PWY-5347 | 0.373 | 0.148 | 0.741 | 0.993 |
| 141 | PWY-5384 | 0.070 | 0.638 | 0.389 | 0.993 |
| 142 | PWY-5415 | 0.076 | 0.219 | 0.374 | 0.993 |
| 143 | PWY-5417 | 0.729 | 0.813 | 0.887 | 0.993 |
| 144 | PWY-5431 | 0.719 | 0.810 | 0.883 | 0.993 |
| 145 | PWY-5484 | 0.054 | 0.268 | 0.350 | 0.993 |
| 146 | PWY-5505 | 0.302 | 0.959 | 0.709 | 0.995 |
| 147 | PWY-5507 | 0.536 | 0.421 | 0.774 | 0.993 |
| 148 | PWY-5509 | 0.382 | 0.829 | 0.747 | 0.993 |
| 149 | PWY-5531 | 0.098 | 0.505 | 0.345 | 0.993 |
| 150 | PWY-5532 | 0.398 | 0.273 | 0.665 | 0.990 |
| 151 | PWY-5659 | 0.608 | 0.519 | 0.853 | 0.993 |
| 152 | PWY-5667 | 0.574 | 0.797 | 0.842 | 0.993 |
| 153 | PWY-5676 | 0.213 | 0.103 | 0.564 | 0.993 |
| 154 | PWY-5677 | 0.036 | 0.242 | 0.226 | 0.993 |
| 155 | PWY-5686 | 0.459 | 0.618 | 0.774 | 0.993 |
| 156 | PWY-5695 | 0.379 | 0.587 | 0.747 | 0.993 |
| 157 | PWY-5705 | 0.865 | 0.126 | 0.939 | 0.993 |
| 158 | PWY-5747 | 0.139 | 0.338 | 0.411 | 0.992 |
| 159 | PWY-5837 | 0.856 | 0.663 | 0.931 | 0.993 |
| 160 | PWY-5838 | 0.401 | 0.699 | 0.752 | 0.993 |
| 161 | PWY-5840 | 0.380 | 0.680 | 0.746 | 0.993 |
| 162 | PWY-5845 | 0.837 | 0.426 | 0.930 | 0.993 |
| 163 | PWY-5850 | 0.836 | 0.423 | 0.930 | 0.993 |
| 164 | PWY-5855 | 0.228 | 0.804 | 0.602 | 0.993 |
| 165 | PWY-5856 | 0.223 | 0.801 | 0.597 | 0.993 |
| 166 | PWY-5857 | 0.227 | 0.802 | 0.600 | 0.993 |
| 167 | PWY-5860 | 0.779 | 0.507 | 0.909 | 0.993 |
| 168 | PWY-5861 | 0.597 | 0.615 | 0.849 | 0.993 |
| 169 | PWY-5862 | 0.769 | 0.509 | 0.906 | 0.993 |
| 170 | PWY-5863 | 0.840 | 0.658 | 0.926 | 0.993 |
| 171 | PWY-5896 | 0.832 | 0.431 | 0.927 | 0.993 |
| 172 | PWY-5897 | 0.384 | 0.666 | 0.747 | 0.993 |
| 173 | PWY-5898 | 0.385 | 0.666 | 0.748 | 0.993 |
| 174 | PWY-5899 | 0.386 | 0.665 | 0.748 | 0.993 |
| 175 | PWY-5910 | 0.374 | 0.749 | 0.745 | 0.993 |
| 176 | PWY-5913 | 0.101 | 0.506 | 0.445 | 0.993 |
| 177 | PWY-5918 | 0.213 | 0.518 | 0.605 | 0.993 |
| 178 | PWY-5920 | 0.355 | 0.388 | 0.724 | 0.993 |
| 179 | PWY-5971 | 0.788 | 0.660 | 0.910 | 0.993 |
| 180 | PWY-5973 | 0.111 | 0.732 | 0.461 | 0.993 |
| 181 | PWY-5989 | 0.535 | 0.833 | 0.820 | 0.993 |
| 182 | PWY-6071 | 0.602 | 0.923 | 0.832 | 0.994 |
| 183 | PWY-6121 | 0.373 | 0.593 | 0.746 | 0.993 |
| 184 | PWY-6122 | 0.395 | 0.617 | 0.750 | 0.993 |
| 185 | PWY-6123 | 0.329 | 0.566 | 0.730 | 0.993 |
| 186 | PWY-6125 | 0.018 | 0.829 | 0.224 | 0.993 |
| 187 | PWY-6126 | 0.620 | 0.393 | 0.855 | 0.993 |
| 188 | PWY-6147 | 0.577 | 0.220 | 0.844 | 0.993 |
| 189 | PWY-6151 | 0.771 | 0.954 | 0.906 | 0.995 |
| 190 | PWY-6163 | 0.798 | 0.737 | 0.914 | 0.993 |
| 191 | PWY-6165 | 0.311 | 0.438 | 0.559 | 0.988 |
| 192 | PWY-6182 | 0.714 | 0.785 | 0.882 | 0.993 |
| 193 | PWY-6185 | 0.610 | 0.628 | 0.832 | 0.993 |
| 194 | PWY-621 | 0.420 | 0.998 | 0.757 | 1.000 |
| 195 | PWY-6269 | 0.382 | 0.831 | 0.746 | 0.993 |
| 196 | PWY-6277 | 0.396 | 0.618 | 0.750 | 0.993 |
| 197 | PWY-6282 | 0.593 | 0.813 | 0.845 | 0.993 |
| 198 | PWY-6317 | 0.560 | 0.999 | 0.839 | 1.000 |
| 199 | PWY-6353 | 0.796 | 0.231 | 0.913 | 0.993 |
| 200 | PWY-6383 | 0.183 | 0.084 | 0.515 | 0.992 |
| 201 | PWY-6385 | 0.314 | 0.620 | 0.721 | 0.993 |
| 202 | PWY-6386 | 0.537 | 0.631 | 0.828 | 0.993 |
| 203 | PWY-6387 | 0.435 | 0.621 | 0.763 | 0.993 |
| 204 | PWY-6396 | 0.104 | 0.959 | 0.450 | 0.995 |
| 205 | PWY-6397 | 0.449 | 0.554 | 0.715 | 0.993 |
| 206 | PWY-6467 | 0.168 | 0.787 | 0.546 | 0.993 |
| 207 | PWY-6470 | 0.672 | 0.362 | 0.871 | 0.993 |
| 208 | PWY-6471 | 0.376 | 0.960 | 0.742 | 0.996 |
| 209 | PWY-6478 | 0.581 | 0.343 | 0.815 | 0.993 |
| 210 | PWY-6507 | 0.012 | 0.791 | 0.201 | 0.993 |
| 211 | PWY-6519 | 0.708 | 0.918 | 0.884 | 0.993 |
| 212 | PWY-6545 | 0.362 | 0.882 | 0.734 | 0.993 |
| 213 | PWY-6562 | 0.590 | 0.835 | 0.825 | 0.993 |
| 214 | PWY-6572 | 0.597 | 0.673 | 0.819 | 0.993 |
| 215 | PWY-6588 | 0.007 | 0.559 | 0.151 | 0.993 |
| **216** | **PWY-6590** | **0.00030** | **0.439** | **0.047** | **0.993** |
| 217 | PWY-6608 | 0.747 | 0.505 | 0.897 | 0.993 |
| 218 | PWY-6609 | 0.041 | 0.824 | 0.309 | 0.993 |
| 219 | PWY-6612 | 0.526 | 0.421 | 0.820 | 0.993 |
| 220 | PWY-6628 | 0.632 | 0.978 | 0.859 | 0.998 |
| 221 | PWY-6629 | 0.266 | 0.407 | 0.549 | 0.992 |
| 222 | PWY-6630 | 0.636 | 0.978 | 0.860 | 0.998 |
| 223 | PWY-6690 | 0.665 | 0.657 | 0.858 | 0.993 |
| 224 | PWY-6700 | 0.171 | 0.455 | 0.550 | 0.993 |
| 225 | PWY-6703 | 0.227 | 0.392 | 0.621 | 0.993 |
| 226 | PWY-6708 | 0.228 | 0.804 | 0.600 | 0.993 |
| 227 | PWY-6737 | 0.707 | 0.355 | 0.884 | 0.993 |
| 228 | PWY-6749 | 0.770 | 0.713 | 0.904 | 0.993 |
| 229 | PWY-6876 | 0.045 | 0.679 | 0.291 | 0.993 |
| 230 | PWY-6891 | 0.156 | 0.908 | 0.531 | 0.993 |
| 231 | PWY-6892 | 0.680 | 0.555 | 0.873 | 0.993 |
| 232 | PWY-6895 | 0.179 | 0.704 | 0.561 | 0.993 |
| 233 | PWY-6897 | 0.868 | 0.407 | 0.941 | 0.993 |
| 234 | PWY-6901 | 0.695 | 0.373 | 0.880 | 0.993 |
| 235 | PWY-6906 | 0.402 | 0.476 | 0.654 | 0.987 |
| 236 | PWY-6969 | 0.042 | 0.891 | 0.314 | 0.993 |
| 237 | PWY-7003 | 0.074 | 0.372 | 0.344 | 0.993 |
| 238 | PWY-7007 | 0.547 | 0.617 | 0.782 | 0.993 |
| 239 | PWY-7013 | 0.698 | 0.884 | 0.880 | 0.993 |
| 240 | PWY-7094 | 0.535 | 0.126 | 0.785 | 0.987 |
| 241 | PWY-7111 | 0.952 | 0.249 | 0.975 | 0.993 |
| 242 | PWY-7159 | 0.107 | 0.473 | 0.358 | 0.993 |
| 243 | PWY-7184 | 0.020 | 0.714 | 0.232 | 0.993 |
| 244 | PWY-7187 | 0.785 | 0.411 | 0.910 | 0.993 |
| 245 | PWY-7196 | 0.006 | 0.808 | 0.177 | 0.993 |
| 246 | PWY-7197 | 0.033 | 0.805 | 0.293 | 0.993 |
| 247 | PWY-7198 | 0.380 | 0.211 | 0.696 | 0.993 |
| 248 | PWY-7199 | 0.037 | 0.192 | 0.301 | 0.993 |
| 249 | PWY-7200 | 0.014 | 0.905 | 0.212 | 0.993 |
| 250 | PWY-7208 | 0.389 | 0.661 | 0.749 | 0.993 |
| 251 | PWY-7210 | 0.368 | 0.222 | 0.690 | 0.993 |
| 252 | PWY-7211 | 0.026 | 0.360 | 0.260 | 0.993 |
| 253 | PWY-7219 | 0.427 | 0.624 | 0.760 | 0.993 |
| 254 | PWY-7220 | 0.722 | 0.247 | 0.889 | 0.993 |
| 255 | PWY-7221 | 0.443 | 0.593 | 0.767 | 0.993 |
| 256 | PWY-7222 | 0.723 | 0.247 | 0.889 | 0.993 |
| 257 | PWY-7228 | 0.017 | 0.832 | 0.222 | 0.993 |
| 258 | PWY-7229 | 0.559 | 0.420 | 0.838 | 0.993 |
| 259 | PWY-7234 | 0.620 | 0.794 | 0.855 | 0.993 |
| 260 | PWY-7237 | 0.776 | 0.442 | 0.908 | 0.993 |
| 261 | PWY-7242 | 0.003 | 0.904 | 0.148 | 0.993 |
| 262 | PWY-7254 | 0.674 | 0.666 | 0.875 | 0.993 |
| 263 | PWY-7255 | 0.546 | 0.514 | 0.784 | 0.993 |
| 264 | PWY-7295 | 0.391 | 0.398 | 0.649 | 0.989 |
| 265 | PWY-7315 | 0.102 | 0.503 | 0.443 | 0.993 |
| 266 | PWY-7323 | 0.364 | 0.994 | 0.739 | 1.000 |
| 267 | PWY-7328 | 0.081 | 0.921 | 0.409 | 0.993 |
| 268 | PWY-7332 | 0.538 | 0.458 | 0.782 | 0.993 |
| 269 | PWY-7376 | 0.537 | 0.401 | 0.778 | 0.993 |
| 270 | PWY-7377 | 0.416 | 0.387 | 0.761 | 0.993 |
| 271 | PWY-7392 | 0.915 | 0.346 | 0.960 | 0.993 |
| 272 | PWY-7400 | 0.254 | 0.838 | 0.654 | 0.993 |
| 273 | PWY-7431 | 0.152 | 0.622 | 0.432 | 0.993 |
| 274 | PWY-7446 | 0.276 | 0.814 | 0.588 | 0.994 |
| 275 | PWY-7456 | 0.022 | 0.858 | 0.237 | 0.993 |
| 276 | PWY-7539 | 0.769 | 0.316 | 0.905 | 0.993 |
| 277 | PWY-7560 | 0.628 | 0.232 | 0.857 | 0.993 |
| 278 | PWY-7616 | 0.244 | 0.761 | 0.528 | 0.994 |
| 279 | PWY-7663 | 0.068 | 0.680 | 0.382 | 0.993 |
| 280 | PWY-7664 | 0.629 | 0.783 | 0.858 | 0.993 |
| 281 | PWY-841 | 0.025 | 0.778 | 0.261 | 0.993 |
| 282 | PWY-922 | 0.415 | 0.744 | 0.758 | 0.993 |
| 283 | PWY0-1061 | 0.773 | 0.910 | 0.904 | 0.993 |
| 284 | PWY0-1241 | 0.425 | 0.394 | 0.765 | 0.993 |
| 285 | PWY0-1261 | 0.119 | 0.423 | 0.471 | 0.993 |
| 286 | PWY0-1277 | 0.712 | 0.620 | 0.882 | 0.993 |
| 287 | PWY0-1296 | 0.183 | 0.811 | 0.567 | 0.993 |
| 288 | PWY0-1297 | 0.153 | 0.836 | 0.527 | 0.993 |
| 289 | PWY0-1298 | 0.089 | 0.817 | 0.424 | 0.993 |
| 290 | PWY0-1319 | 0.573 | 0.797 | 0.842 | 0.993 |
| 291 | PWY0-1338 | 0.616 | 0.754 | 0.839 | 0.993 |
| 292 | PWY0-1415 | 0.654 | 0.087 | 0.866 | 0.993 |
| 293 | PWY0-1479 | 0.840 | 0.905 | 0.930 | 0.993 |
| 294 | PWY0-1533 | 0.773 | 0.177 | 0.905 | 0.993 |
| 295 | PWY0-1586 | 0.084 | 0.690 | 0.414 | 0.993 |
| 296 | PWY0-162 | 0.021 | 0.822 | 0.241 | 0.993 |
| 297 | PWY0-166 | 0.066 | 0.591 | 0.378 | 0.993 |
| 298 | PWY0-321 | 0.589 | 0.934 | 0.827 | 0.994 |
| 299 | PWY0-41 | 0.200 | 0.798 | 0.554 | 0.994 |
| 300 | PWY0-42 | 0.231 | 0.296 | 0.505 | 0.988 |
| 301 | PWY0-781 | 0.617 | 0.606 | 0.856 | 0.993 |
| 302 | PWY0-845 | 0.383 | 0.922 | 0.744 | 0.994 |
| 303 | PWY0-862 | 0.323 | 0.047 | 0.727 | 0.993 |
| 304 | PWY1G-0 | 0.468 | 0.879 | 0.736 | 0.995 |
| 305 | PWY490-3 | 0.877 | 0.458 | 0.942 | 0.993 |
| 306 | PWY4FS-7 | 0.173 | 0.893 | 0.553 | 0.993 |
| 307 | PWY4FS-8 | 0.174 | 0.892 | 0.554 | 0.993 |
| 308 | PWYG-321 | 0.619 | 0.814 | 0.855 | 0.993 |
| 309 | PYRIDNUCSAL-PWY | 0.887 | 0.149 | 0.948 | 0.993 |
| 310 | PYRIDNUCSYN-PWY | 0.599 | 0.290 | 0.850 | 0.993 |
| 311 | PYRIDOXSYN-PWY | 0.435 | 0.947 | 0.769 | 0.995 |
| 312 | REDCITCYC | 0.435 | 0.075 | 0.769 | 0.993 |
| 313 | RHAMCAT-PWY | 0.075 | 0.548 | 0.396 | 0.993 |
| 314 | RIBOSYN2-PWY | 0.692 | 0.338 | 0.879 | 0.993 |
| 315 | RUMP-PWY | 0.328 | 0.391 | 0.730 | 0.993 |
| 316 | SALVADEHYPOX-PWY | 0.736 | 0.252 | 0.893 | 0.993 |
| 317 | SER-GLYSYN-PWY | 0.830 | 0.378 | 0.927 | 0.993 |
| 318 | SO4ASSIM-PWY | 0.153 | 0.528 | 0.523 | 0.993 |
| 319 | SULFATE-CYS-PWY | 0.229 | 0.519 | 0.627 | 0.993 |
| 320 | TCA | 0.069 | 0.781 | 0.384 | 0.993 |
| 321 | TCA-GLYOX-BYPASS | 0.377 | 0.920 | 0.737 | 0.994 |
| 322 | TEICHOICACID-PWY | 0.114 | 0.210 | 0.463 | 0.993 |
| 323 | THISYN-PWY | 0.029 | 0.402 | 0.279 | 0.993 |
| 324 | THREOCAT-PWY | 0.641 | 0.793 | 0.855 | 0.993 |
| 325 | THRESYN-PWY | 0.951 | 0.262 | 0.975 | 0.993 |
| 326 | TRNA-CHARGING-PWY | 0.392 | 0.459 | 0.751 | 0.993 |
| 327 | TRPSYN-PWY | 0.099 | 0.645 | 0.442 | 0.993 |
| 328 | TYRFUMCAT-PWY | 0.451 | 0.345 | 0.714 | 0.988 |
| 329 | UBISYN-PWY | 0.231 | 0.837 | 0.602 | 0.993 |
| 330 | UDPNAGSYN-PWY | 0.110 | 0.423 | 0.458 | 0.993 |
| 331 | VALDEG-PWY | 0.482 | 0.348 | 0.739 | 0.993 |
| 332 | VALSYN-PWY | 0.824 | 0.221 | 0.920 | 0.993 |
